# Determinants of Task-Based Exposures to Alpha-Diketones in Coffee Roasting and Packaging Facilities Using a Bayesian Model Averaging Approach

**DOI:** 10.3389/fpubh.2022.878907

**Published:** 2022-06-09

**Authors:** Brie Hawley Blackley, Caroline P. Groth, Jean M. Cox-Ganser, Alyson R. Fortner, Ryan F. LeBouf, Xiaoming Liang, Mohammed Abbas Virji

**Affiliations:** ^1^Respiratory Health Division, National Institute for Occupational Safety and Health, Morgantown, WV, United States; ^2^Department of Epidemiology and Biostatistics, School of Public Health, West Virginia University, Morgantown, WV, United States

**Keywords:** task-based, exposure determinants, correlated predictors, coffee, Bayesian model averaging, alpha-diketones, diacetyl, 2,3-pentanedione

## Abstract

Coffee production workers can be exposed to inhalational hazards including alpha-diketones such as diacetyl and 2,3-pentanedione. Exposure to diacetyl is associated with the development of occupational lung disease, including obliterative bronchiolitis, a rare and irreversible lung disease. We aimed to identify determinants contributing to task-based exposures to diacetyl and 2,3-pentanedione at 17 U.S. coffee production facilities. We collected 606 personal short-term task-based samples including roasting (*n* = 189), grinding (*n* = 74), packaging (*n* = 203), quality control (QC, *n* = 44), flavoring (*n* = 15), and miscellaneous production/café tasks (*n* = 81), and analyzed for diacetyl and 2,3-pentanedione in accordance with the modified OSHA Method 1013/1016. We also collected instantaneous activity-based (*n* = 296) and source (*n* = 312) samples using evacuated canisters. Information on sample-level and process-level determinants relating to production scale, sources of alpha-diketones, and engineering controls was collected. Bayesian mixed-effect regression models accounting for censored data were fit for overall data (all tasks) and specific tasks. Notable determinants identified in univariate analyses were used to fit all plausible models in multiple regression analysis which were summarized using a Bayesian model averaging method. Grinding, flavoring, packaging, and production tasks with ground coffee were associated with the highest short-term and instantaneous-activity exposures for both analytes. Highest instantaneous-sources of diacetyl and 2,3-pentanedione included ground coffee, flavored coffee, liquid flavorings, and off-gassing coffee bins or packages. Determinants contributing to higher exposures to both analytes in all task models included sum of all open storage sources and average percent of coffee production as ground coffee. Additionally, flavoring ground coffee and flavoring during survey contributed to notably higher exposures for both analytes in most, but not all task groups. Alternatively, general exhaust ventilation contributed to lower exposures in all but two models. Additionally, among facilities that flavored, local exhaust ventilation during flavoring processes contributed to lower 2,3-pentanedione exposures during grinding and packaging tasks. Coffee production facilities can consider implementing additional exposure controls for processes, sources, and task-based determinants associated with higher exposures to diacetyl and 2,3-pentanedione, such as isolating, enclosing, and directly exhausting grinders, flavoring mixers, and open storage of off-gassing whole bean and ground coffee, to reduce exposures and minimize risks for lung disease among workers.

## Introduction

Coffee production is a global industry and produced an estimated 23.2 billion pounds (lbs) of coffee in 2020/2021, representing an increase of 15.4 million lbs from the previous year (1). The number of workers employed in the coffee industry in the United States has risen to meet increased demand with an estimated 17,704 workers employed in 2019 representing ~0.01% of the U.S. workforce (2), up 11% from 2016 (3). Workers in coffee production can be exposed to multiple inhalational hazards associated with negative health outcomes such as carbon monoxide (4–7), green coffee bean and roasted coffee dust (8–10), and volatile organic compounds including the alpha-diketones diacetyl and 2,3-pentanedione (11–14).

Diacetyl and 2,3-pentanedione are naturally occurring in roasted coffee beans and are also found in some liquid flavorings used to flavor coffee (15). Exposure to diacetyl is associated with the development of occupational respiratory disease, including obliterative bronchiolitis, a rare and irreversible lung disease that results in inflammation and narrowing of the bronchioles and symptoms such as cough, shortness of breath, and wheeze (16–18). 2,3-Pentanedione, a structurally similar chemical to diacetyl and often used as a substitute for diacetyl in flavorings, causes airway fibrosis, including obliterative bronchiolitis-like changes in rodents after repeated inhalation exposure (15, 19). To reduce the risk of respiratory impairment and severe irreversible lung disease, the National Institute for Occupational Safety and Health (NIOSH) developed recommended exposure limits (RELs) of 5 parts per billion (ppb) for diacetyl and 9.3 ppb for 2,3-pentanedione for time-weighted average (TWA) full-shift exposures (20). Additionally, NIOSH recommended short-term exposure limits (STELs) of 25 ppb for diacetyl and 31 ppb for 2,3-pentanedione averaged over a 15-min time period.

A cluster of obliterative bronchiolitis was observed among former workers of a coffee roasting and packaging facility between 2008 and 2015 (21, 22). The cluster of obliterative bronchiolitis was publicized in coffee trade magazines and spurred concerns among coffee companies and employees, prompting some to submit health hazard evaluation (HHE) requests to NIOSH. NIOSH performed HHE investigations at 17 coffee roasting and packaging facilities in response to these requests during 2016 and 2017. A summary of full-shift, short-term task-based, and instantaneous exposures to diacetyl and 2,3-pentanedione at these 17 facilities is reported in LeBouf et al. (13). Lebouf et al. observed that 11–77% of personal full-shift diacetyl samples collected at non-flavoring facilities and 62–95% of personal full-shift 2,3-pentanedione samples collected at flavoring facilities exceeded their respective RELs (13). Personal task exposures were orders of magnitude higher than full-shift exposures and had larger geometric standard deviations (GSDs) than many full-shift exposures. Measurements of elevated exposures to diacetyl and 2,3-pentanedione highlighted a need to understand determinants of exposures to alpha-diketones in coffee production facilities such that exposure mitigation strategies can be designed and implemented accordingly.

Statistical modeling of exposure determinants has been used extensively in the field of industrial hygiene to (1) predict exposures for use in epidemiologic studies or for risk assessment and (2) understand factors affecting exposures such as exposure duration, source strength, proximity to sources, and existing exposure controls, to subsequently identify, prioritize, and implement exposure mitigation strategies (23, 24). New approaches using Bayesian statistics have recently been proposed and used to account for parameter uncertainties, censored data, and to take advantage of the ease of Bayesian inferences (13, 25). Specifically, Bayesian model averaging (BMA) methods not only address the uncertainties in model building and variable selection, but also address limit of detection issues, repeated measurements on individuals and provides posterior distribution of parameters for all variables considered. Further, this modeling approach can be easily implemented in R-software using RJAGS and in other Bayesian programs such as OpenBUGS or Stan (26–28).

Despite the need, no previous studies have evaluated factors contributing to elevated task-based exposures to diacetyl and 2,3-pentanedione in coffee production facilities. Here, we expand upon the summary of exposures and emissions in coffee roasting facilities and cafés, reported in LeBouf et al., by identifying (1) tasks, activities, and sources associated with elevated short-term and instantaneous exposures to diacetyl and 2,3-pentanedione, and (2) determinants associated with elevated, or reduced, short-term, task-based exposures to diacetyl and 2,3-pentanedione using a BMA approach.

## Methods

### Facility Characteristics

Roasted coffee production at sampled facilities ranged from 16 to 4,500 tons per year and total number of employees ranged from 4 to 150 workers. Air samples were collected between July 2015 and September 2017 across different seasons, in a variety of geographical locations, with differing amounts of natural ventilation occurring from open doors or windows. LeBouf et al. provides additional information on the facilities, including information relating to production scale and process-related factors [Table 1 of LeBouf et al. (13)].

### Sampling Approach

The full sampling approach utilized in the exposure assessment surveys performed at the 17 coffee roasting and packaging facilities was described previously in LeBouf et al. (13). Here, we provide a brief summary of the sampling approach used to collect short-term task-based, and instantaneous activity-based breathing zone and source air samples. Sampling at each facility was initiated by an HHE request. Workers were asked to voluntarily participate in the air sampling surveys, which lasted 2–4 days at each facility, and provided their informed consent prior to participating.

### Short-Term Task-Based Air Sampling and Analysis

We collected 606 personal short-term task-based samples in worker's breathing zones during various tasks including roasting (*n* = 189), grinding (*n* = 74), packaging (*n* = 203), QC (*n* = 44), flavoring (*n* = 15), cleaning machines (*n* = 36), moving roasted beans/ground coffee (*n* = 13), miscellaneous production (*n* = 17), miscellaneous café (*n* = 10), and maintenance (*n* = 5). A full description of the various sub-tasks included in each of these 10 task categories can be found in Supplementary Table 1 of LeBouf et al. (13). Repeat samples were collected for tasks on the same day and over multiple days whenever possible.

Short-term task-based samples were collected on silica gel tubes (SKC Inc., Eighty Four, PA) and analyzed for diacetyl and 2,3-pentanedione according to the modified OSHA Sampling and Analytical Methods 1013/1016. Two glass silica gel sorbent tubes were connected with tubing and placed in a protective light-blocking cover and sampled at a flow rate of 200 mL/min. Sample analyses were performed in the NIOSH Respiratory Health Division's Organics Laboratory. The median limits of detection (LODs) were 0.9 ppb for diacetyl and 1.0 ppb for 2,3-pentanedione.

### Instantaneous Activity-Based and Source Air Sampling and Analysis

We collected 296 instantaneous activity-based breathing zone samples, and 312 instantaneous source air samples using evacuated canisters for diacetyl and 2,3-pentanedione. A full list of volatile organic compounds (VOCs) analyzed in instantaneous canister samples can be found in LeBouf et al. (13).

Instantaneous canister samples were collected and analyzed in accordance with NIOSH method 3900 (29). The sampler consisted of a 450-mL evacuated canister (Entech Instruments, Inc., Simi Valley, CA) equipped with an instantaneous fitting designed for a short sampling duration (<30 s). For activity-based air samples, the inlet of the canister was opened and held near the worker's breathing zone for <30 s while they performed an activity. For source air samples, the inlet of the canister was opened and held for <30 s directly at the source of interest. Median LODs were 0.6 ppb for diacetyl and 0.8 ppb for 2,3-pentanedione, based on a 1.5-times dilution factor, which is typical for instantaneous samples. However, individual LOD concentrations varied because they depended on the sample volume inside each canister.

### Sample-Level and Process-Level Determinants

The source-receptor model described by Tielemans et al. was used to conceptualize factors that might modify inhalational exposure to diacetyl and 2,3-pentanedione during tasks (30). Source-receptor model factors included source strength (e.g., whole bean or ground coffee), transport of the contaminant through different compartments (e.g., process isolation), and loss of contaminants (e.g., local exhaust ventilation). Information on sample-level and process-level factors relating to production scale, sources of alpha-diketones, and engineering controls were collected prior to, during, and after completion of surveys. Descriptions of sample-level and process-level factors can be found in Supplementary Table 1.

Task-based sample-level factors included roaster characteristics (e.g., roaster capacity and enclosed/unenclosed), grinder characteristics (e.g., grinder's typical weight of ground coffee processed), coffee characteristics during grinding task (e.g., grinding flavored or unflavored coffee), coffee characteristics during packaging task (e.g., packaging volume), and sampled task type (Supplementary Tables 1, 4, 7). Process-level determinants did not vary within a facility and were systematically collected on forms prior to or after sampling. Process-level factors included coffee storage determinants (e.g., sum of all open storage sources present in a facility), general sources determinants (e.g., total number of sources of diacetyl or 2,3-pentanedione), amount of roasted coffee produced (e.g., average roasted coffee production in lbs per day), roast depth (e.g., average roast length in minutes), amount of grinding performed and grinding processes (e.g., average percent of production as ground coffee), flavoring process determinants (e.g., flavor ground coffee and isolated flavoring room), automation of sources (e.g., percent automated sources), isolation of sources (e.g., any isolated sources/processes), enclosure of sources (e.g., any enclosed sources), and mechanical ventilation type [e.g., general exhaust ventilation (GEV)] (Supplementary Tables 1, 5–7).

### Statistical Modeling

Statistical analyses were performed using R (version 4.0 or greater; R Foundation for Statistical Computing), JMP 15.0 and SAS 9.4 (SAS Institute, Inc., Cary, NC). In addition, all Bayesian analyses were programmed using rjags (27) and data were organized and summarized in figures in R using tidyverse and ggplot2. Diacetyl and 2,3-pentanedione measurements were log-transformed allowing for the use of ANOVA and linear regression-based methods. Repeated measures analyses were used to account for within subject variability when sufficient numbers of workers with repeated measurements (*n* >5) were included in sample sets.

Bayesian modeling strategies accounting for censored data were used throughout our analyses because up to 24 and 27% of task-based samples for diacetyl and 2,3-pentanedione were below their respective LODs (25, 31). In all cases, priors were selected to be as weakly informative as possible to allow the data to drive the inference. Specifically, priors for regression coefficients or mean parameters were specified to be a wide normal distribution with mean 0 and variance 1,000,000. Fixed effect models used inverse-gamma priors on the variances [with shape = 0.1 and scale = 0.1 as described in Gelman et al. (32)]. Repeated-measures random effect models used uniform priors on the standard deviations with a range of ln (1.01) to ln (500). Convergence was assessed using trace plots and Markov chain Monte Carlo (MCMC) standard error. Models suggested almost immediate convergence (within 5,000 iterations). Each linear regression model chain with at least one predictor was thinned to keep only every 60th iteration to avoid autocorrelation in the chains. All estimates provided in tables or figures are for the median posterior estimate and the respective credible interval (based on quantiles of the posterior distribution).

Additionally, in all analyses, we assumed independence, linearity, equal variances, and normality of residuals, consistent with linear regressions. Non-linear relationships were not explored. Similarly, lognormality of each chemical was assumed, and other distributions were not explored.

#### Descriptive Analysis of Task-Based Samples and Instantaneous Activity/Source Based Samples

Exposure estimates for short-term task and instantaneous activity and source exposures were generated using a Bayesian intercept only (ANOVA; no predictors) model. Short-term task estimates further accounted for repeated measures. A total of 20,000 iterations after 5,000 iterations of burn-in were used to develop posterior exposure estimates of the GM, GSD, and 95th percentile for each short-term task and instantaneous exposure distribution. Instantaneous canister samples were summarized for various personal or source activities. Additional details of the methods used in the descriptive analysis of instantaneous samples can be found in the Supplementary Material.

#### Task-Based Univariate Determinant Models

We aimed to identify determinants affecting exposures across all tasks as well as task-specific determinants. Thus, we generated (1) an overall model to identify determinants of exposure across all tasks, and (2) task-specific models to identify additional sample-level and task-process specific determinants. We performed a series of single-variable Bayesian linear regression models with each individual determinant separately to identify important determinants of exposures. Determinants were designated as notable if the 80% credible interval (Bayesian uncertainty interval) for the regression estimate of any slope did not include 0. Additional information on how determinants of short-term task-based exposure to diacetyl or 2,3-pentanedione were created can be found in the Supplementary Material.

We conducted a series of single-variable Bayesian regression models accounting for repeated measurements for the overall, roasting, grinding, and packaging models. Facility was not included as a random effect due to too few subjects (<5) observed at many facilities resulting in insufficient information to estimate the variance components in a nested random effect model; thus, we used a model with random effect for subjects only. Additionally, QC (*n* = 44) and flavoring models (*n* = 15) had too few measurements to run repeated measures models. Therefore, we used a simpler fixed effect form of the Bayesian regression models to identify notable determinants. Multiple linear regression models for QC and flavoring were also not developed due to small sample sizes.

#### Multicollinearity Check

Many determinants were expected to be highly correlated with one another due to many process-based determinants being related to each other. Inclusion of multiple correlated predictors would lead to multicollinearity/collinearity and increased standard errors on the regression estimates. Therefore, to determine collinear combinations of determinants for each model, we calculated Pearson correlations of each pair of notable determinants. We developed Pearson correlations for each category above reference of a determinant using indicators. It is commonly agreed that correlations >0.5 will result in multicollinearity (33, 34). Pairs of determinants with 0.5 level correlation or greater (and in at least one category if a categorical variable) were noted as multicollinear and were excluded from entry into the same models. In addition, some variables were identified as nested when one variable was a subset of another variable. All nested variables were also excluded from entry into the same models to avoid the inclusion of redundant variables in the same models.

#### Bayesian Model Averaging

To develop estimates of each variable's contribution to exposure for diacetyl and 2,3-pentanedione, we used a BMA approach. BMA performs a set of Bayesian linear regressions that considers all possible combinations of predictors (as fixed effects). The models are then summarized over the regression estimates.

We developed a list of all possible models for each model type (task model and analyte). Each model was developed to account for repeated measures and to account for measurements below the LOD. Then, using information from the multicollinearity check, we removed any models with any multicollinear combination of predictors. Thus, the final BMA approach considered a subset of possible models.

We utilized GSD reduction, calculated as the overall GSD in the null model minus the overall GSD in the models with determinants, as an alternative metric to R-squared to evaluate the need for weighting models. R-squared is avoided in Bayesian methods, especially when Bayesian methods are utilized to estimate values below the LOD, because R-squared statistics could be misleading as they will estimate the variance of the censored measurements to be low at each iteration. After experimenting with weighting models by relative contribution (% GSD reduction), we observed that weighted models did not result in substantial changes to estimates compared to unweighted models which is likely related to a lack in substantial overall GSD reduction (analysis not shown). As a result, we did not weight models when calculating parameter estimates for each determinant.

Each model was run for 10,000 iterations after 5,000 iterations of burn-in (after thinning every 60 iterations). We performed the averaging process at each iteration of the process for each model containing the determinant providing a full posterior distribution of the average regression parameter estimate across models. Averaging was performed only in the models in which the determinant was included; this was done intentionally to avoid shrinkage toward 0 in the estimates of the coefficients for determinants that were not included in all models (due to collinearity).

The relative magnitude of effect of each determinant on exposure to diacetyl or 2,3-pentanedione was assessed by calculating the percent change (1) for categorical variables compared to the reference category or (2) per x units of a continuous variable. Percent change was calculated based on the following formula: Percent Change = (exp(beta_k × units_k)−1) × 100 where beta_k is the regression coefficient estimate and units_k is the units of measure for the regression coefficient k. We defined a notable or credible difference to be present when the 95% credible interval (CI) for the slope coefficient or the percentage change does not contain 0.

## Results

### Comparison of Diacetyl and 2,3-Pentanedione Measurements

Log-transformed diacetyl and 2,3-pentanedione air concentrations from task-based samples (*n* = 606) were positively correlated with a Spearman's correlation coefficient of *rho* = 0.507.

### Short-Term Task-Based Exposure Summary

**Figure 1:**
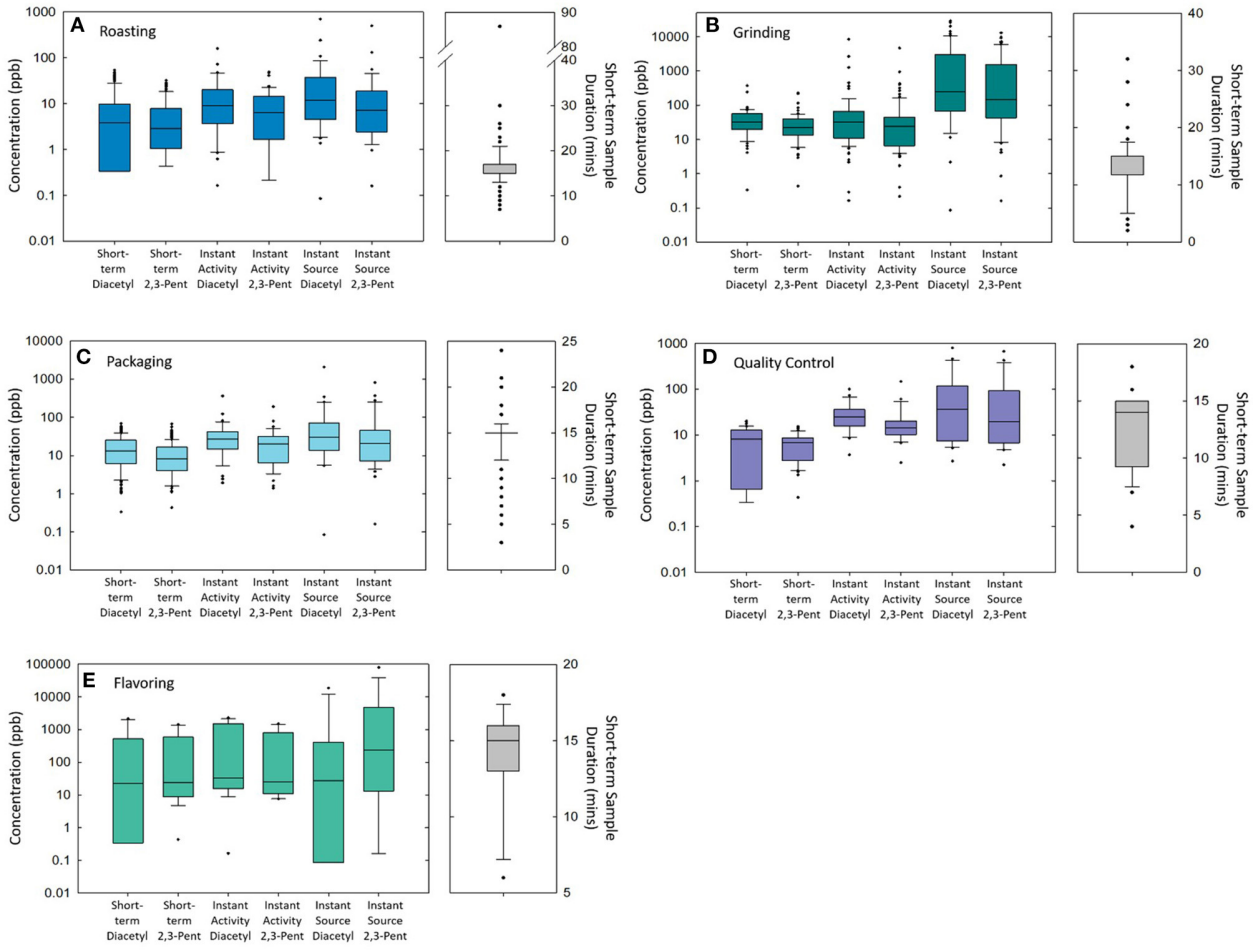
A panel of box plots of diacetyl and 2,3-pentanedione concentrations measured in short-term task-based, instantaneous activity, and instantaneous source samples and short-term task-based sample durations during roasting tasks **(A)**, grinding tasks **(B)**, packaging tasks **(C)**, QC tasks **(D)**, and flavoring tasks **(E)**.

A summary of personal short-term task-based exposures and sample durations (range: 2–86 min) can be seen in Figure 1 and Table 1. Overall, the highest task-based exposures for diacetyl and 2,3-pentanedione across all facilities were measured during grinding (GMs = 27.8 and 22.7 ppb, respectively), flavoring (GMs = 5.4 and 45.1 ppb), and moving roasted beans or ground coffee (GMs = 21.7 and 13.1 ppb). Large variability (GSD = 30.2) was observed for diacetyl exposures during flavoring tasks.

**Table 1:** Personal short-duration task exposures to diacetyl and 2,3-pentanedione.

**Task**	**Sampling duration (min–max)**	***N***	**k**	**Diacetyl**						**2,3-Pentanedione**					
				**GM (ppb)**	**GSD**	**P95 (ppb)**	**%BDL**	***N*** **15 min samples**	**% >STEL (*N*)**	**GM (ppb)**	**GSD**	**P95 (ppb)**	**%BDL**	***N*** **15 min samples**	**% >STEL (*N*)**
Flavoring coffee	6–18	15	5	5.4	30.2	1,102	27%	6	50% (3)	45.1	14.8	3,817	7%	6	50% (3)
Grinding coffee	2–32	74	32	27.8	2.7	147	4%	35	69% (24)	22.7	2.6	112	1%	35	34% (12)
Moving roasted beans or ground coffee	3–25	13	9	21.7	2.7	109	0%	6	50% (3)	13.1	2.6	63	0%	6	0% (0)
Cleaning machines	5–46	36	18	10.3	4.0	102	11%	11	64% (7)	6.5	3.4	49	14%	11	0% (0)
Packaging coffee	3–55	203	74	11.3	2.9	66	5%	136	24% (32)	7.0	2.9	40	8%	136	6% (8)
Misc. production	3–29	17	9	4.7	4.4	52	18%	4	0% (0)	2.7	3.9	25	24%	4	0% (0)
Roasting coffee	7–86	189	34	3.6	5.3	55	25%	70	13 (9)	3.1	4.1	32	24%	70	0% (0)
QC	4–18	44	9	3.9	3.7	33	25%	14	0% (0)	5.5	2.1	19	2%	14	0% (0)
Miscellaneous café tasks	5–16	10	6	2.2	4.5	25	30%	4	0% (0)	3.5	2.5	16	10%	4	0% (0)
Maintenance of machines	13–15	5	1	–	–	15*	20%	4	0% (0)	–	–	7.8*	20%	4	0% (0)

N, number of samples; k, number of workers; GM, geometric mean; ppb, parts per billion; GSD, geometric standard deviation; P95, 95th percentile; %BDL, percent samples below the limit of detection; N 15 min samples, number of 15 minute samples for comparison to NIOSH STEL; % >STEL (N), percent of 15 min samples greater than NIOSH STEL for diacetyl or 2,3-pentanedione; STEL, NIOSH short-term exposure limit of 25 ppb diacetyl and 31 ppb 2,3-pentanedione; ^*^indicates where maximum is presented when <5 measurements were above the detection limit; –indicates not enough samples above the detection limit to obtain an estimate.

Of the samples collected for 15 min duration and available for comparison with the NIOSH STELs, 50% (*n* = 3/6) of flavoring tasks, 69% (*n* = 24/35) of grinding tasks, 50% (*n* = 3/6) of moving roasted beans or ground coffee tasks, 24% (*n* = 32/136) of packaging tasks, 13% (*n* = 9/70) of roasting tasks, and 64% (*n* = 7/11) of cleaning machines tasks exceeded the NIOSH STEL of 25 ppb diacetyl (Table 1). Additionally, 50% (*n* = 3/6) of flavoring tasks, 34% (*n* = 12/35) of grinding tasks, and 6% (*n* = 8/136) of packaging tasks exceeded the NIOSH STEL of 31 ppb 2,3-pentanedione.

### Instantaneous Activity-Based and Source Exposure Summary

A summary of instantaneous activity-based and source samples can be seen in Figure 1 and Supplementary Tables 2, 3. Instantaneous activity and source samples summarized in Figure 1 are those associated with roasting (Figure 1A), grinding (Figure 1B), packaging (Figure 1C), QC (Figure 1D), and flavoring (Figure 1E) tasks and processes. Descriptive statistics (GM, GSD, P95) for instantaneous activity-based and source samples can be seen in Supplementary Tables 2, 3.

### Univariate Analyses of Determinants Contributing to Short-Term Task-Based Exposures

**Figure 2:**
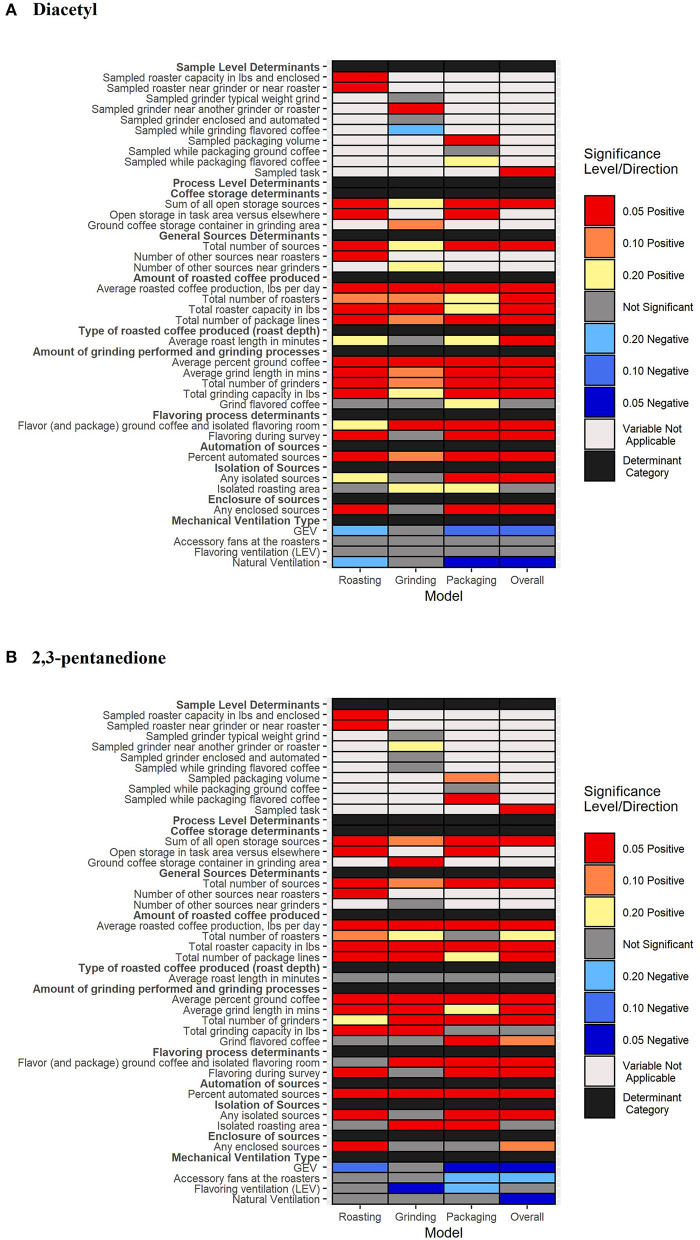
A heat-map of significant determinants identified in univariate analyses considered for models of diacetyl **(A)** and 2,3-pentanedione **(B)** exposure. Notable credible intervals (CIs) are depicted for 80, 90, and 95% CIs. Positive associations are depicted with warm colors (yellow, orange, and red) and negative associations are depicted with cool colors (light blue to dark blue).

Determinant distributions and univariate model estimates of regression coefficients and 80% CIs are reported in Supplementary Tables 4–7. A color-coded heat map of determinants contributing to increased or decreased short-term, task-based exposures in coffee production overall as well as during specific tasks such as roasting, grinding, and packaging can be seen in Figures 2A,B. Additionally, univariate model estimates of GMs and 80% credible intervals for diacetyl and 2,3-pentanedione concentrations in each determinant category and each task category (all tasks, roasting, grinding, and packaging) can be seen in Supplementary Figures S1–S4. A summary of the univariate analyses for each individual task as well as for all tasks is provided in the Supplementary Materials.

### Multiple Linear Regression Models of Determinants Contributing to Short-Term Task-Based Exposures

**Figure 3:**
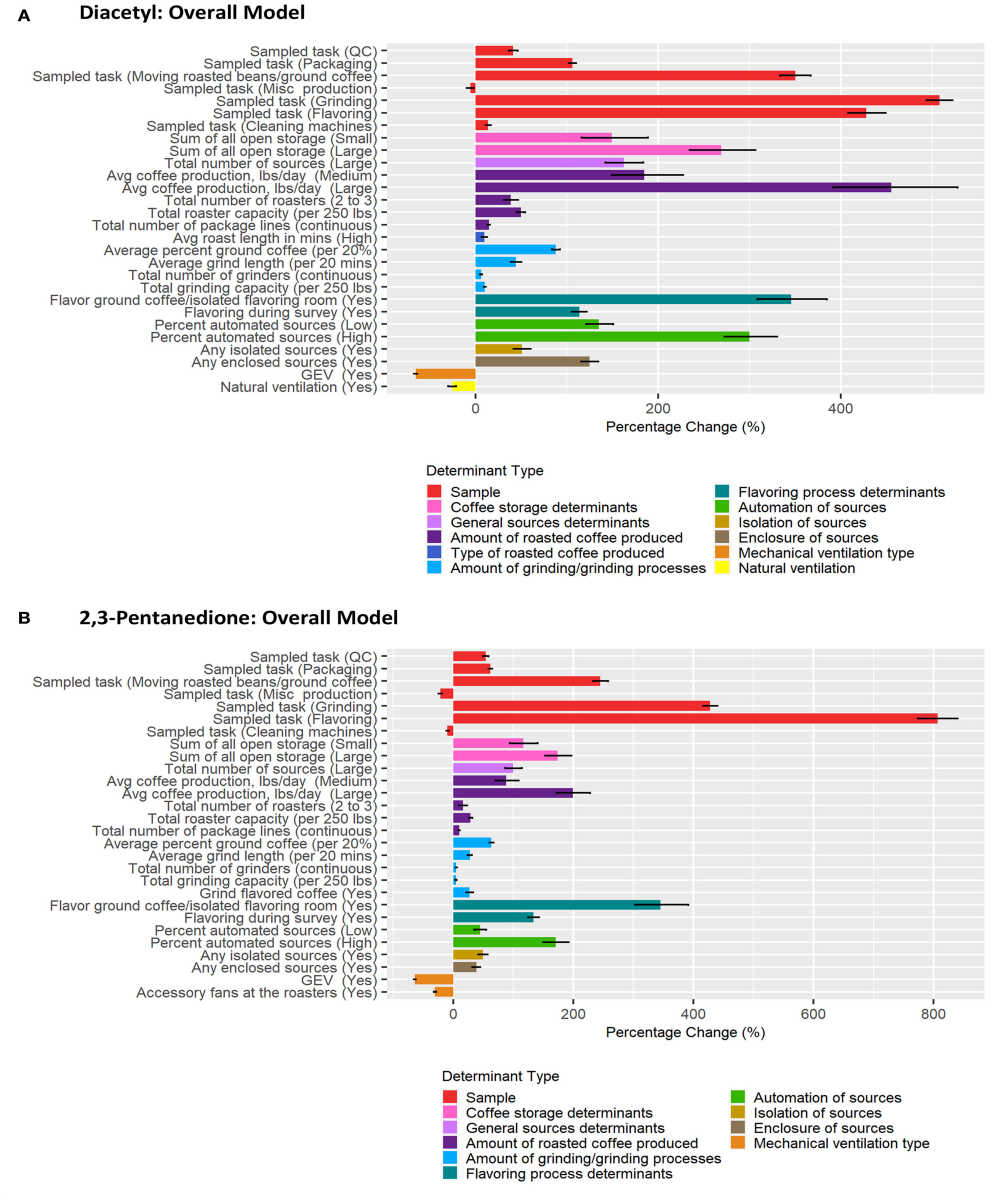
Bar chart of percent change in diacetyl **(A)** and 2,3-pentanedione **(B)** exposures during all tasks (overall model) compared with reference category or per unit change. Each bar represents the estimated percent change for the median posterior estimate with error bars for the 95% credible intervals. Percent change for continuous variables defined as per 1 unit with the exceptions of the following: total roaster capacity and total grinder capacity are calculated per 250 lbs, average percent ground coffee is calculated per increase in 20%, and average grind length in minutes is calculated per 20 min.

**Figure 4:**
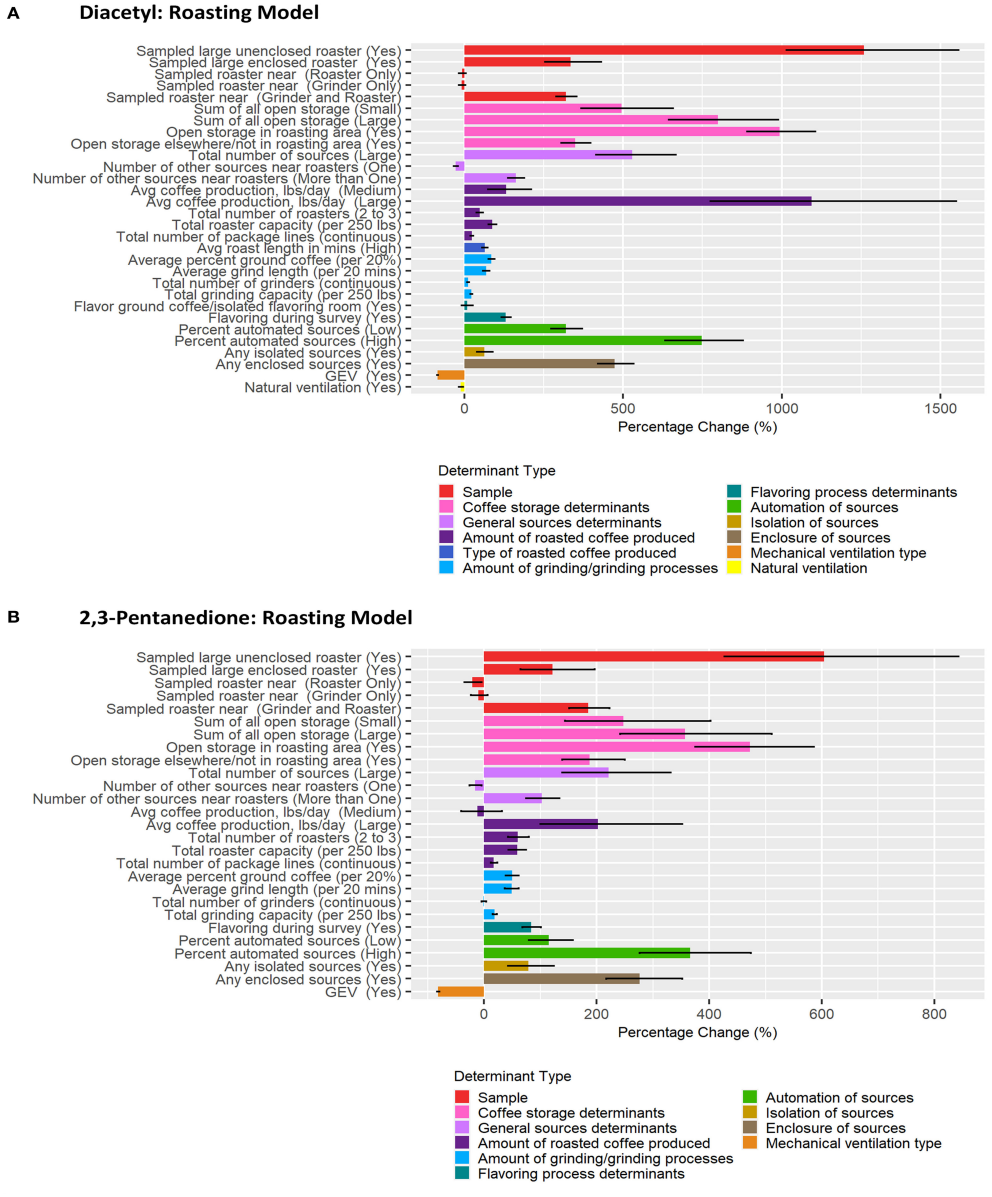
Bar chart of percent change in diacetyl **(A)** and 2,3-pentanedione **(B)** exposures during roasting tasks compared with reference category or per unit change. Each bar represents the estimated percent change for the median posterior estimate with error bars for the 95% credible intervals. Percent change for continuous variables defined as per 1 unit with the exceptions of the following: total roaster capacity and total grinder capacity are calculated per 250 lbs, average percent ground coffee is calculated per increase in 20%, and average grind length in minutes is calculated per 20 min.

**Figure 5:**
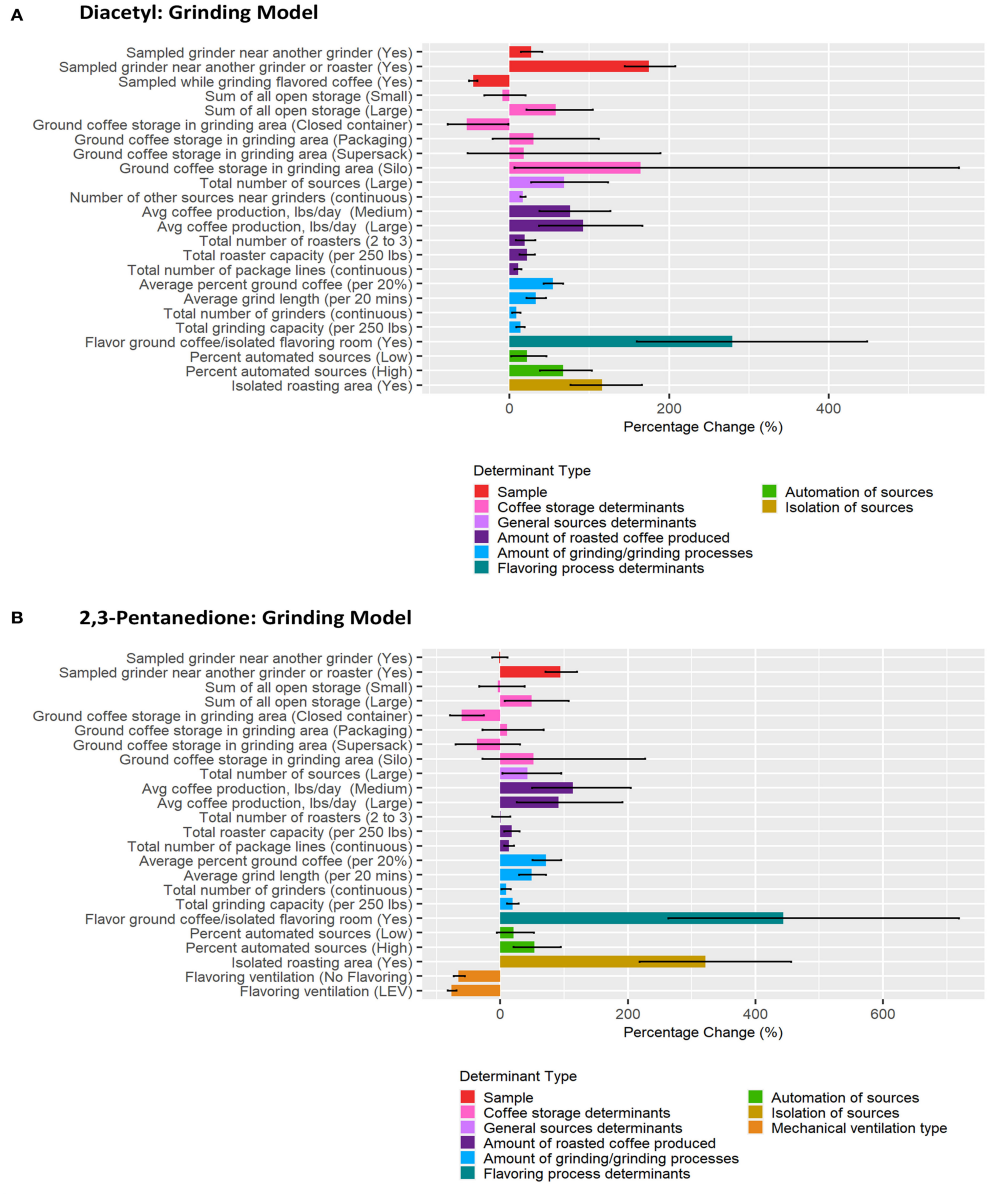
Bar chart of percent change in diacetyl **(A)** and 2,3-pentanedione **(B)** exposures during grinding tasks compared with reference category or per unit change. Each bar represents the estimated percent change for the median posterior estimate with error bars for the 95% credible intervals. Percent change for continuous variables defined as per 1 unit with the exceptions of the following: total roaster capacity and total grinder capacity are calculated per 250 lbs, average percent ground coffee is calculated per increase in 20%, and average grind length in minutes is calculated per 20 min.

**Figure 6:**
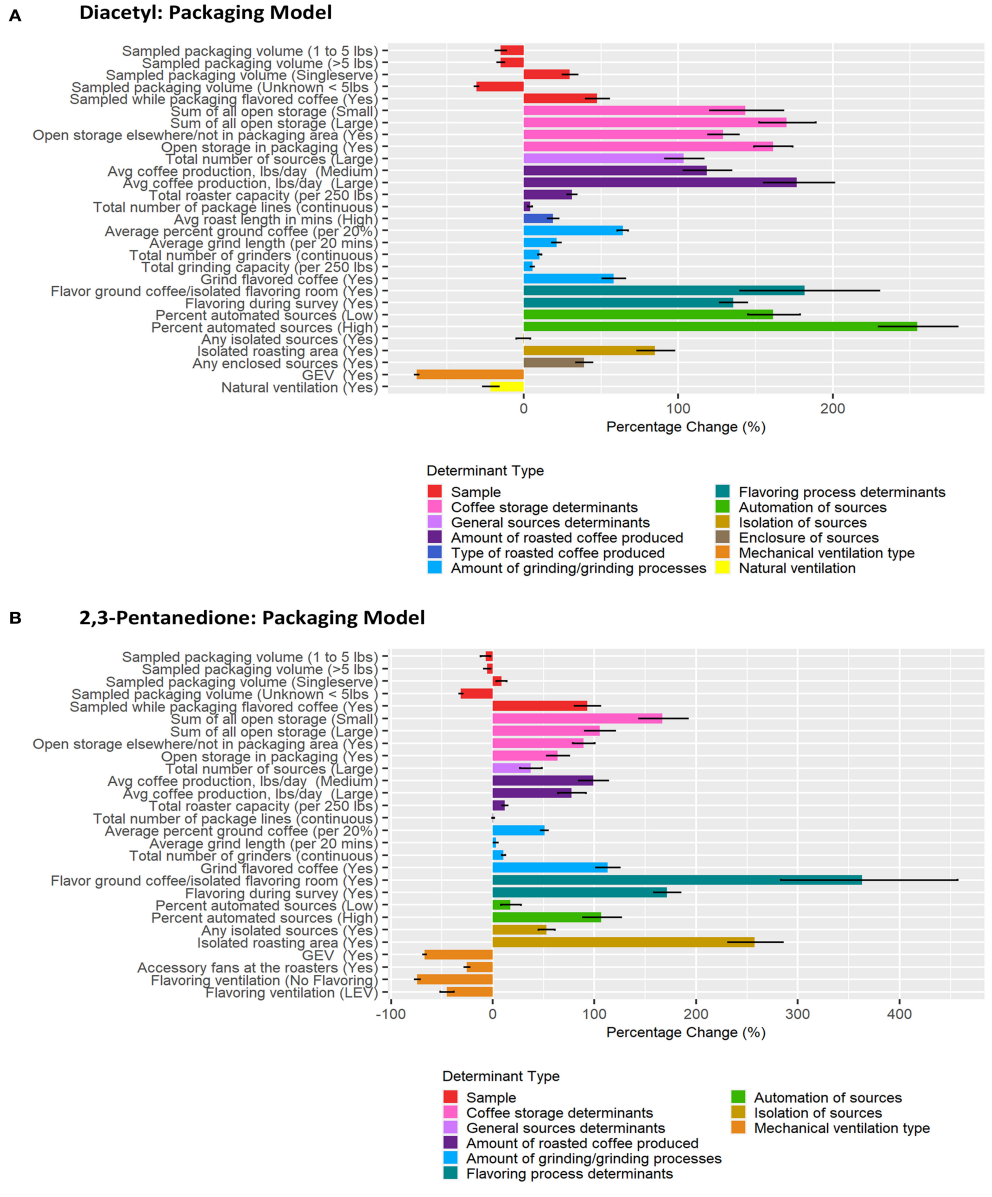
Bar chart of percent change in diacetyl **(A)** and 2,3-pentanedione **(B)** exposures during packaging tasks compared with reference category or per unit change. Each bar represents the estimated percent change for the median posterior estimate with error bars for the 95% credible intervals. Percent change for continuous variables defined as per 1 unit with the exceptions of the following: total roaster capacity and total grinder capacity are calculated per 250 lbs, average percent ground coffee is calculated per increase in 20%, and average grind length in minutes is calculated per 20 min.

Correlation matrices for all determinants identified in the previous step as notable at the 0.2 level on univariate analyses for each task model can be seen in Supplementary Figures S5–S8. BMA results are provided in Figures 3–6 and Tables 2–5. Average regression estimates across all multiple linear regression models and the 95% CI for each determinant that was identified as notable (i.e., 95% CI did not contain 0 for the slope) in single determinant analyses can be seen in Tables 2–5. The number and percent of models containing the determinant are also included in Tables 2–5. The percentage change in exposures for the given coefficient above reference along with the 95% CI can be seen in Figures 3–6. We note that the percent change is judged relative to the reference condition and should be interpreted alongside the intercept reference value for each respective BMA model.

**Table 2:** Bayesian model averaging results for diacetyl and 2,3-pentanedione exposures during all tasks (overall task model).

**Determinant**	**Diacetyl** ***N***_**total**_ **= 483**				**2,3-pentanedione** ***N***_**total**_ **= 483**			
	***N*** **models**	**% models included**	**Median posterior estimate - β**	**95% CI**	***N*** **models**	**% models included**	**Median posterior estimate - β**	**95% CI**
**Sample level determinants**								
Sampled task (QC)	242	50.1	0.34	(0.31, 0.38)	242	50.1	0.43	(0.4, 0.46)
Sampled task (packaging)	242	50.1	0.72	(0.7, 0.74)	242	50.1	0.48	(0.46, 0.5)
Sampled task (moving roasted beans/ground coffee)	242	50.1	1.5	(1.47, 1.54)	242	50.1	1.24	(1.2, 1.28)
Sampled task (Misc production)	242	50.1	−0.06	(−0.1, −0.02)	242	50.1	−0.24	(−0.28, −0.2)
Sampled task (grinding)	242	50.1	1.8	(1.78, 1.83)	242	50.1	1.66	(1.64, 1.69)
Sampled task (flavoring)	242	50.1	1.66	(1.63, 1.71)	242	50.1	2.2	(2.17, 2.24)
Sampled task (cleaning machines)	242	50.1	0.13	(0.1, 0.15)	242	50.1	−0.1	(−0.12, −0.08)
**Coffee storage determinants**								
Sum of all open storage (small, 1–2)	16	3.3	0.91	(0.77, 1.06)	24	5	0.77	(0.66, 0.88)
Sum of all open storage (large, >2)	16	3.3	1.31	(1.21, 1.4)	24	5	1.01	(0.93, 1.09)
**General sources determinants**								
Total number of sources (>7)	24	6	0.96	(0.88, 1.04)	32	6.6	0.69	(0.62, 0.76)
**Amount of roasted coffee produced**								
Avg coffee production, lbs/day (Medium, ≥1,000 lbs and <10,000 lbs)	16	3.3	1.05	(0.91, 1.18)	24	5	0.63	(0.53, 0.74)
Avg coffee production, lbs/day (Large, >10,000 lbs )	16	3.3	1.71	(1.59, 1.84)	24	5	1.1	(1, 1.19)
Total number of roasters (2, 3)	72	14.9	0.33	(0.27, 0.38)	52	10.8	0.15	(0.09, 0.21)
Total roaster capacity (per 250 lbs)	16	3.3	0.0016	(0.0015, 0.0017)	24	5	0.26	(0.23, 0.28)
Total number of package lines (continuous)	24	4.97	0.13	(0.12, 0.15)	24	5	0.09	(0.08, 0.11)
**Type of roasted coffee produced (roast depth)**								
Avg roast length in mins (high, ≥15 min)	234	48.5	0.09	(0.06, 0.12)	–	–	–	–
**Amount of grinding performed and grinding process determinants**								
Average percent ground coffee (per 20%)	72	14.9	0.032	(0.030, 0.033)	84	17.4	0.49	(0.47, 0.51)
Average grind length (per 20 min)	16	3.31	0.018	(0.016, 0.020)	40	8.3	0.24	(0.22, 0.27)
Total number of grinders (continuous)	92	19.1	0.06	(0.05, 0.07)	72	14.9	0.05	(0.04, 0.06)
Total grinding capacity (per 250 lbs)	84	17.4	0.0004	(0.0003, 0.0004)	64	13.3	0.04	(0.04, 0.05)
**Flavoring process determinants**								
Flavor ground coffee/isolated flavoring room (yes)	108	22.4	1.49	(1.41, 1.58)	66	13.7	1.49	(1.39, 1.59)
Flavoring during survey (yes)	144	29.8	0.76	(0.72, 0.80)	98	20.3	0.85	(0.81, 0.89)
**Engineering controls determinants**								
Percent automated sources (low, ≤ 6)	48	9.9	0.85	(0.79, 0.92)	36	7.5	0.37	(0.3, 0.44)
Percent automated sources (high, >6)	48	9.9	1.39	(1.31, 1.46)	36	7.5	1	(0.92, 1.07)
Any isolated sources/processes (yes)	54	11.2	0.41	(0.35, 0.47)	64	13.3	0.4	(0.35, 0.45)
Any enclosed sources (yes)	80	16.6	0.81	(0.77, 0.85)	72	14.9	0.33	(0.28, 0.37)
GEV (yes)	170	35.2	−1.08	(−1.13, −1.03)	216	44.7	−1.02	(−1.05, −0.98)
Natural ventilation (yes)	100	20.7	−0.30	(−0.36, −0.24)	–	–	–	–
Grind flavored coffee (yes)	–	–	–	–	160	33.1	0.24	(0.2, 0.29)
Accessory fans at the roasters (yes)	–	–	–	–	188	38.9	−0.36	(−0.39, −0.34)

*N_total_, total number of models; N, indicates number of models determinant was included in; %, percent; –, determinants that were not applicable (>0.2 on univariate analyses); 95% CI, 95% credible intervals*.

**Table 3:** Bayesian model averaging results for diacetyl and 2,3-pentanedione exposures during roasting tasks.

**Determinant**	**Diacetyl** ***N***_**total**_ **= 837**				**2,3-pentanedione** ***N***_**total**_ **= 240**			
	***N*** **models**	**% models included**	**Median posterior estimate - β**	**95% CI**	***N*** **models**	**% models included**	**Median posterior estimate - β**	**95% CI**
**Sample level determinants**								
Sampled roaster capacity (large) and unenclosed	36	4.3	2.61	(2.41, 2.81)	12	5.0	1.95	(1.66, 2.24)
Sampled roaster capacity (large) and enclosed	36	4.3	1.47	(1.27, 1.67)	12	5.0	0.8	(0.5, 1.09)
Sampled roaster near another roaster	158	19	−0.07	(−0.19, 0.06)	48	20.1	−0.23	(−0.42, −0.05)
Sampled roaster near another grinder	158	19	−0.08	(−0.18, 0.03)	48	20.1	−0.1	(−0.27, 0.06)
Sampled roaster near roaster and grinder	158	19	1.44	(1.36, 1.51)	48	20.1	1.05	(0.92, 1.17)
**Coffee storage determinants**								
Sum of all open storage (small, 1–2)	24	2.9	1.78	(1.54, 2.03)	8	3.3	1.25	(0.89, 1.61)
Sum of all open storage (large, >2)	24	2.9	2.2	(2.01, 2.39)	8	3.3	1.52	(1.23, 1.81)
Open storage in roasting area	102	12.3	2.39	(2.29, 2.49)	24	10	1.74	(1.56, 1.93)
Open storage elsewhere/not in roasting area	102	12.3	1.5	(1.4, 1.6)	24	10	1.06	(0.87, 1.25)
**General sources determinants**								
Total number of sources (>7)	24	2.9	1.84	(1.64, 2.04)	8	3.3	1.17	(0.87, 1.46)
Number of other sources near roasters (1)	252	30.3	−0.31	(−0.39, −0.23)	72	30.1	−0.17	(−0.30, −0.04)
Number of other sources near roasters (>1)	252	30.3	0.99	(0.88, 1.10)	72	30.1	0.7	(0.56, 0.85)
**Amount of roasted coffee produced**								
Avg coffee production, lbs/day (Medium, ≥1,000 lbs and <10,000 lbs)	24	2.9	0.84	(0.56, 1.14)	8	3.3	−0.12	(−0.53, 0.27)
Avg coffee production, lbs/day (large, >10,000 lbs)	24	2.9	2.48	(2.17, 2.8)	8	3.3	1.11	(0.7, 1.51)
Total number of roasters (2, 3)	308	37.1	0.39	(0.32, 0.46)	88	36.8	0.47	(0.36, 0.59)
Total roaster capacity (per 250 lbs)	24	3.8	0.63	(0.57, 0.7)	8	4.4	0.46	(0.36, 0.56)
Total number of package lines (continuous)	24	2.9	0.21	(0.18, 0.25)	8	3.3	0.16	(0.11, 0.21)
**Type of roasted coffee produced (roast depth)**								
Avg roast length in mins (High, ≥15 min)	416	50.1	0.50	(0.45, 0.55)	–	–	–	–
**Amount of grinding performed and grinding process determinants**								
Average percent ground coffee (per 20%)	76	9.1	0.62	(0.56, 0.67)	28	11.7	0.40	(0.33, 0.48)
Average grind length (per 20 min)	72	8.7	0.53	(0.47, 0.58)	24	10.0	0.40	(0.32, 0.48)
Total number of grinders (continuous)	96	11.6	0.11	(0.09, 0.14)	24	10.0	−0.01	(−0.05, 0.04)
Total grinding capacity (per 250 lbs)	96	11.6	0.21	(0.19, 0.23)	32	13.4	0.17	(0.15, 0.20)
**Flavoring process determinants**								
Flavor ground coffee/isolated flavoring room (yes)	144	17.3	0.09	(−0.08, 0.24)	–	–	–	–
Flavoring during survey (yes)	288	34.7	0.84	(0.77, 0.90)	100	41.8	0.61	(0.52, 0.70)
**Engineering controls determinants**								
Percent automated sources (low, ≤ 6)	88	10.6	1.44	(1.32, 1.55)	32	13.4	0.77	(0.58, 0.95)
Percent automated sources (high, >6)	88	10.6	2.14	(1.99, 2.28)	32	13.4	1.54	(1.33, 1.75)
Any isolated sources/processes (yes)	60	7.2	0.49	(0.34, 0.64)	16	6.7	0.58	(0.35, 0.81)
Any enclosed sources (yes)	102	12.3	1.75	(1.65, 1.85)	24	10.0	1.33	(1.15, 1.51)
GEV (yes)	292	35.1	−1.83	(−1.92, −1.75)	120	50.2	−1.68	(−1.8, −1.58)
Natural ventilation (yes)	248	29.8	−0.11	(−0.19, −0.03)	–	–	–	–

*N_total_, total number of models; N models, number of models determinant was included in; %, percent; –, determinants that were not applicable (>0.2 on univariate analyses); 95% CI, 95% credible intervals*.

**Table 4:** Bayesian model averaging results for diacetyl and 2,3-pentanedione exposures during grinding tasks.

**Determinant**	**Diacetyl** ***N***_**total**_ **= 154**				**2,3-pentanedione** ***N***_**total**_ **= 76**			
	***N*** **models**	**% models included**	**Median posterior estimate - β**	**95% CI**	***N*** **models**	**% models included**	**Median posterior estimate - β**	**95% CI**
**Sample level determinants**								
Sampled grinder near another grinder	49	31.8	0.24	(0.13, 0.34)	35	46.1	−0.01	(−0.13, 0.11)
Sampled grinder near another grinder and roaster	49	31.8	1.01	(0.89, 1.13)	35	46.1	0.66	(0.53, 0.79)
Sampled while grinding flavored coffee (yes)	68	44.2	−0.61	(−0.7, −0.51)	–	–	–	–
**Coffee storage determinants**								
Sum of all open storage (small, 1–2)	12	7.8	−0.09	(−0.38, 0.18)	6	7.9	−0.04	(−0.4, 0.33)
Sum of all open storage (large, >2)	12	7.8	0.45	(0.19, 0.72)	6	7.9	0.4	(0.07, 0.73)
Ground coffee storage in grinding area (closed container)	4	2.6	−0.77	(−1.49, −0.01)	4	5.3	−0.94	(−1.57, −0.3)
Ground coffee storage in grinding area (packaging)	4	2.6	0.26	(−0.24, 0.75)	4	5.3	0.1	(−0.33, 0.52)
Ground coffee storage in grinding area (supersack)	4	2.6	0.17	(−0.75, 1.06)	4	5.3	−0.46	(−1.2, 0.27)
Ground coffee storage in grinding area (silo)	4	2.6	0.97	(0.06, 1.89)	4	5.3	0.42	(−0.33, 1.18)
**General sources determinants**								
Total number of sources (>7)	8	5.2	0.52	(0.24, 0.8)	6	7.9	0.35	(0.03, 0.67)
Number of other sources near grinders (continuous)	49	31.8	0.16	(0.13, 0.18)	–	—-	–	–
**Amount of roasted coffee produced**								
Avg coffee production, lbs/day (medium, ≥1,000 lbs and <10,000 lbs)	8	5.2	0.57	(0.32, 0.82)	3	3.9	0.76	(0.4, 1.11)
Avg coffee production, lbs/day (large, >10,000 lbs)	8	5.2	0.65	(0.32, 0.98)	3	3.9	0.65	(0.23, 1.07)
Total number of roasters (2, 3)	50	32.5	0.18	(0.07, 0.28)	24	31.6	0	(−0.13, 0.14)
Total roaster capacity (per 250 lbs)	12	7.8	0.2	(0.12, 0.28)	6	7.9	0.16	(0.06, 0.26)
Total number of package lines (continuous)	6	3.9	0.1	(0.06, 0.14)	2	2.6	0.13	(0.06, 0.19)
**Amount of grinding performed and grinding process determinants**								
Average percent ground coffee (per 20%)	24	15.6	0.43	(0.36, 0.51)	8	10.5	0.54	(0.41, 0.67)
Average grind length (per 20 min)	12	7.8	0.29	(0.19, 0.38)	4	5.3	0.4	(0.26, 0.54)
Total number of grinders (continuous)	9	5.8	0.08	(0.03, 0.13)	6	7.9	0.09	(0.02, 0.15)
Total grinding capacity (per 250 lbs)	12	7.8	0.13	(0.08, 0.17)	4	5.3	0.18	(0.1, 0.25)
**Flavoring process determinants**								
Flavor ground coffee/isolated flavoring room (yes)	15	9.7	1.33	(0.95, 1.7)	10	13.2	1.69	(1.29, 2.1)
**Engineering controls determinants**								
Percent automated sources (low, ≤ 6)	30	19.5	0.2	(0.02, 0.38)	16	21.1	0.19	(−0.06, 0.42)
Percent automated sources (high, >6)	30	19.5	0.51	(0.32, 0.71)	16	21.1	0.43	(0.19, 0.67)
Isolated roasting area (yes)	34	22.1	0.77	(0.57, 0.98)	13	17.1	1.44	(1.16, 1.72)
Flavoring ventilation (NA, no flavoring)	NA	NA	NA	NA	17	22.4	−1.06	(−1.3, −0.82)
Flavoring ventilation (LEV)	NA	NA	NA	NA	17	22.4	−1.45	(−1.74, −1.16)

*N_total_, total number of models; N models, number of models determinant was included in; %, percent; –, determinants that were not applicable (>0.2 on univariate analyses); 95% CI, 95% credible intervals; NA, not applicable; LEV, local exhaust ventilation*.

**Table 5:** Bayesian model averaging results for diacetyl and 2,3-pentanedione exposures during packaging tasks.

**Determinant**	**Diacetyl** ***N***_**total**_ **= 927**				**2,3-pentanedione** ***N***_**total**_ **= 631**			
	***N*** **models**	**% models included**	**Median posterior estimate - β**	**95% CI**	***N*** **models**	**% models included**	**Median posterior estimate - β**	**95% CI**
**Sample level determinants**								
Packaging 1–5 lbs	362	35.5	−0.16	(−0.2, −0.12)	224	35.5	−0.07	(−0.12, −0.02)
Packaging >5 lbs	362	35.5	−0.16	(−0.19, −0.14)	224	35.5	−0.06	(−0.09, −0.02)
Packaging single serve ground coffee	362	35.5	0.26	(0.22, 0.3)	224	35.5	0.08	(0.03, 0.13)
Packaging unknown, <5 lbs	362	35.5	−0.37	(−0.39, −0.35)	224	35.5	−0.37	(−0.4, −0.35)
Packaging flavored coffee (yes)	165	16.2	0.39	(0.34, 0.44)	104	16.5	0.66	(0.59, 0.72)
**Coffee storage determinants**								
Sum of all open storage (small, 1–2)	42	4.1	0.89	(0.79, 0.99)	48	7.6	0.98	(0.89, 1.07)
Sum of all open storage (large, >2)	42	4.1	0.99	(0.93, 1.06)	48	7.6	0.72	(0.65, 0.79)
Open storage elsewhere/not in packaging area	206	20.2	0.83	(0.79, 0.87)	108	17.1	0.64	(0.58, 0.69)
Open storage in packaging	206	20.2	0.96	(0.91, 1.01)	108	17.1	0.49	(0.43, 0.56)
**General sources determinants**								
Total number of sources (>7)	52	5.1	0.71	(0.65, 0.77)	34	5.4	0.32	(0.24, 0.39)
**Amount of roasted coffee produced**								
Avg coffee production, lbs/day (medium, ≥1,000 lbs and <10,000 lbs)	84	8.2	0.78	(0.71, 0.85)	72	11.4	0.69	(0.61, 0.76)
Avg coffee production, lbs/day (large, >10,000 lbs )	84	8.2	1.02	(0.94, 1.1)	72	11.4	0.57	(0.5, 0.65)
Total roaster capacity (per 250 lbs)	40	3.9	0.27	(0.25, 0.29)	44	7	0.11	(0.09, 0.14)
Total number of package lines (continuous)	40	3.9	0.04	(0.03, 0.05)	66	10.5	0	(0, 0.01)
**Type of roasted coffee produced (roast depth)**								
Avg roast length in mins (high, ≥15 min)	297	29.1	0.17	(0.14, 0.2)	–	–	–	–
**Amount of grinding performed and grinding process determinants**								
Average percent ground coffee (per 20%)	125	12.3	0.49	(0.47, 0.52)	100	15.8	0.41	(0.39, 0.43)
Average grind length (per 20 min)	65	6.4	0.19	(0.17, 0.22)	92	14.6	0.03	(0.01, 0.05)
Total number of grinders (continuous)	110	10.8	0.1	(0.09, 0.11)	44	7	0.1	(0.09, 0.11)
Total grinding capacity (per 250 lbs)	73	7.2	0.05	(0.04, 0.06)	–	–	–	–
Grind flavored coffee (yes)	222	21.8	0.46	(0.41, 0.51)	140	22.2	0.76	(0.7, 0.81)
**Flavoring process determinants**								
Flavor ground coffee/isolated flavoring room (yes)	39	3.8	1.04	(0.88, 1.19)	30	4.8	1.53	(1.34, 1.72)
Flavoring during survey (yes)	172	16.9	0.86	(0.82, 0.89)	100	15.8	1	(0.95, 1.05)
**Engineering controls determinants**								
Percent automated sources (low, ≤ 6)	92	9	0.96	(0.9, 1.03)	62	9.8	0.16	(0.08, 0.24)
Percent automated sources (high, >6)	92	9	1.27	(1.19, 1.34)	62	9.8	0.72	(0.64, 0.82)
Any isolated sources/processes (yes)	185	18.2	0	(−0.05, 0.04)	144	22.8	0.42	(0.37, 0.48)
Isolated roasting area (yes)	162	15.9	0.61	(0.55, 0.68)	102	16.2	1.27	(1.2, 1.35)
Any enclosed sources (yes)	170	16.7	0.33	(0.29, 0.37)	–	–	–	–
GEV (yes)	410	40.2	−1.18	(−1.22, −1.15)	316	50.1	−1.11	(−1.15, −1.07)
Accessory fans at the roasters (yes)	–	–	–	–	192	30.4	−0.29	(−0.33, −0.26)
Flavoring ventilation (NA, no flavoring)	–	–	–	–	50	7.9	−1.35	(−1.44, −1.26)
Flavoring ventilation (LEV)	–	–	–	–	50	7.9	−0.6	(−0.72, −0.48)

*N_total_, total number of models; N models, number of models determinant was included in; %, percent; –, determinants that were not applicable (>0.2 on univariate analyses); 95% CI, 95% credible intervals; NA, not applicable; LEV, local exhaust ventilation*.

#### Overall Tasks

A total of 483 overall task diacetyl models and 483 overall task 2,3-pentandione models were generated comprising 19 determinants for both diacetyl and 2,3-pentanedione exposures (Table 2). Among sample-level determinants, task type, specifically, performing grinding or flavoring tasks, were associated with the highest increases in diacetyl and 2,3-pentanedione exposures when compared to the reference group of roasting tasks (Table 2; Figure 3). Across models containing sampled task, grinding was associated with a median increase of 508% in diacetyl concentrations (95% CI: 494–522%) and 428% increase in 2,3-pentanedione concentrations (95% CI: 416–440%) compared to the reference task (Figure 3). Similarly, flavoring task was associated with a median increase of 428% in diacetyl concentrations (95% CI: 408–449%) and 806% median increase in 2,3-pentanedione concentrations (95% CI: 774–840%) above the reference group of roasting tasks (Figure 3). We note that the highest increases for diacetyl and 2,3-pentanedione (e.g., 508% increase in diacetyl for grinding tasks and 806% increase for flavoring tasks) are large, albeit relative, increases. For context when interpreting percentage change, the intercept reference value across all models was 6.0 ppb diacetyl and 6.3 ppb 2,3-pentanedione.

For process-level determinants, the top five production-level determinants with greatest impact on increasing diacetyl exposures compared to their respective reference categories included (1) >10,000 lbs average daily roasted coffee production, (2) flavor ground coffee, (3) >6% automated sources, (4) >2 sources of open storage, and (5) >1,000 lbs of average roasted coffee production, with 95% CIs of percent increases ranging from (1) 392–528%, (2) 309–385%, (3) 272–330%, (4) 234–307% and (5) 149–227%, respectively (Figure 3).

Process-level determinants associated with notable increases in 2,3-pentanedione exposure in the overall task model were similar to those for diacetyl, although the exact order was not the same. Additional determinants identified as notable for 2,3-pentanedione exposures in the overall task model included grinding flavored coffee (reference: grinding only unflavored coffee) (Table 2; Figure 3). Average roast length in minutes was identified as a determinant associated with notable increases in diacetyl, but not 2,3-pentanedione (Figure 3). The top five production-level determinants with the greatest impact on increasing 2,3-pentanedione exposures were (1) flavor ground coffee, (2) >10,000 lbs average daily roasted coffee production, (3) >2 sources of open storage, (4) >6% automated sources, and (5) flavoring during survey, with 95% CIs of percent increases ranging from (1) 303–392%, (2) 173–228%, (3) 153–197%, (4) 150–192%, and (5) 124–143%, respectively (Figure 3).

Process-level determinants associated with notable decreases in diacetyl or 2,3-pentanedione exposure in the overall task model included GEV and natural ventilation (Table 2; Figure 3). GEV was associated with estimated 95% CIs of percent decreases ranging from 64.1 to 67.6% in diacetyl and 62.3 to 65.2% in 2,3-pentanedione (95% CIs, Figure 3). Natural ventilation was associated with 95% CIs of percent decreases ranging from 21.5 to 29.9% for diacetyl but not 2,3-pentanedione (Figure 3). Additionally, accessory fans at the roasters were associated with 95% CIs of percent decreases ranging from 28.6 to 32.3% for 2,3-pentanedione but not diacetyl.

#### Roasting Tasks

A total of 837 roasting task diacetyl models and 240 roasting task 2,3-pentanedione models were generated comprising 22 and 19 determinants for diacetyl and 2,3-pentanedione exposures, respectively (Table 3). Determinants associated with increases in diacetyl and 2,3-pentanedione exposure during roasting tasks were similar to those identified in the overall task model although the order was not the same. Additional roasting specific process-level determinants associated with increases in diacetyl and 2,3-pentanedione during roasting tasks included open storage of coffee in the roasting area and >1 source near the roasters (Table 3; Figure 4). Also, unlike the overall model, flavor ground coffee did not contribute to increased diacetyl exposures and total number of grinders did not contribute to increased 2,3-pentanedione exposures during roasting tasks.

Among sample-level determinants, sampled roaster capacity (large) and unenclosed was associated with some of the highest increases in diacetyl exposures compared to the reference category of sampled small unenclosed roasters (Table 5; Figure 4). This effect was notably larger for large unenclosed roasters (95% CI: 1,014–1,556% increase in diacetyl; 427–843% increase in 2,3-pentanedione) than for a large enclosed roaster (95% CI: 255–431% increase in diacetyl; 65–197% increase in 2,3-pentanedione) (Figure 4). Additionally, a sampled roaster being near another roaster and grinder was associated with notable increases of both diacetyl and 2,3-pentanedione (95% CI: 289–354% increase in diacetyl; 151–223% increase in 2,3-pentanedione) when compared to the reference of a sampled roaster not near any grinders or roasters (Table 3; Figure 4). We note that although an increase ranging from 1,014–1,556% for diacetyl and 427–843% is large, for context, the intercept reference values for diacetyl and 2,3-pentanedione across all roasting models was 3.0 ppb diacetyl and 3.9 ppb 2,3-pentanedione.

Among process-level determinants, the top five with the greatest impact on increasing diacetyl exposures during roasting tasks were (1) open storage of coffee in the roasting area, (2) >10,000 lbs average daily roasted coffee production, (3) >2 sources of open storage, (4) high percent (>6%) automated sources, and (5) large number of total sources (>7), with 95% CIs of percent increases ranging from (1) 890–1,104%, (2) 775–1,548%, (3) 644–988%, (4) 632–878%, and (5) 414–667%, respectively. The top five process-level determinants with greatest impact on increasing 2,3-pentanedione exposures during roasting tasks were (1) open storage of coffee in the roasting area, (2) high percent (>6%) automated sources, (3) >2 sources of open storage, (4) any enclosed source, and (5) 1–2 sources of open storage, with 95% CIs of percent increases ranging from (1) 375–586%, (2) 277–474%, (3) 242–511%, (4) 217–352%, and (5) 144–402%, respectively (Figure 4). Some differences were noted between the models for diacetyl and 2,3-pentanedione. Total number of grinders and average roast length in minutes contributed to notable increases in diacetyl exposure but not 2,3-pentanedione exposure.

Process-level determinants associated with decreases in exposure to diacetyl or 2,3-pentanedione during roasting tasks, listed from highest to lowest median percent decreases, included: GEV (included in both diacetyl and 2,3-pentanedione models), natural ventilation (included in diacetyl model) and one source near the roasters (included in both diacetyl and 2,3-pentanedione models) (Table 3; Figure 4). GEV was associated with estimated 95% CIs of percent decreases ranging from 80.5 to 84.3% decreases in diacetyl and 79.4 to 83.4% decreases in 2,3-pentanedione (Figure 4). Natural ventilation was associated with estimated 95% CI of percent decreases ranging from 3.0 to 17.2% decreases in diacetyl (Figure 4).

#### Grinding Tasks

A total of 154 grinding task diacetyl models and 76 grinding task 2,3-pentanedione models were generated comprising 17 and 16 determinants for diacetyl and 2,3-pentanedione exposures, respectively (Table 4). Determinants associated with increases in diacetyl and 2,3-pentanedione exposure during grinding tasks were similar as those noted in the overall task model (e.g., coffee storage determinants, total number of sources, amount of coffee produced, amount of grinding performed, flavoring ground coffee). Some grinding specific determinants were also notable in the grinding model: silo containers for ground coffee storage in the grinding area (reference: open containers) and number of other sources near the grinder were associated with increases in diacetyl but not 2,3-pentanedione exposures (Table 4; Figure 5). Additionally, isolated roasting area was associated with increased diacetyl and 2,3-pentanedione exposures during grinding tasks but not in the overall model.

Among sample-level determinants, sampled grinder near another grinder and roaster was associated with some of the highest increases in diacetyl (95% CI: 144–208%) and 2,3-pentanedione (95% CI: 70.6–120.2%) exposures during grinding tasks compared to the reference category of the sampled grinder not being near another grinder or roaster (Table 4; Figure 5). This effect was smaller but also notable for diacetyl exposures for sampled grinders near another grinder only (95% CI: 14–41% increase) (Figure 5). For context when interpreting percentage change, the intercept reference values for diacetyl and 2,3-pentanedione across all grinding models was 23.3 ppb diacetyl and 22.7 ppb 2,3-pentanedione.

Among process-level determinants, the top five process-level determinants with the greatest impact on increasing diacetyl exposures during grinding tasks were (1) flavor ground coffee, (2) silo storage containers for ground coffee in the grinding area (reference: open containers), (3) isolated roasting area, (4) >10,000 lbs average daily roasted coffee production, and (5) 1,000–10,000 lbs average daily roasted coffee production, with 95% CIs of percent increases ranging from (1) 159–448%, (2) 5.9–563%, (3) 77–166%, (4) 37–166%, and (5) 37–127%, respectively. Similarly, the top five process-level determinants with the greatest impact on increasing 2,3-pentanedione exposures during grinding tasks were (1) flavor ground coffee, (2) isolated roasting area, (3) 1,000–10,000 lbs average daily roasted coffee production, (4) >10,000 lbs average daily roasted coffee production, and (5) average percent ground coffee, with 95% CIs of percent increases ranging from (1) 263–719%, (2) 219–456%, (3) 49–205%, and (4) 26–192%, and (5) 51–95%, respectively (Figure 5).

Multiple sample and process-level determinants were associated with decreases in diacetyl or 2,3-pentanedione exposure during grinding tasks. Sampled while grinding flavored coffee (reference: sampled while grinding unflavored coffee) was associated with decreases ranging from 40.1–50.5% for diacetyl (95% CIs, Figure 5). Closed containers for storage of ground coffee in the grinding area (reference: open containers) was associated with 95% CIs of percent decreases ranging from 1.4 to 77.5% for diacetyl and 25.9–79.1% for 2,3-pentanedione, compared to the reference category of open containers (Figure 5). Flavoring processes performed with ventilation in the form of local exhaust ventilation (LEV) was associated with 95% CIs of percent decreases ranging from 68.5 to 82.5% for 2,3-pentanedione during grinding tasks, compared with the reference group of flavoring processes performed with no LEV (Figure 5).

#### Packaging Tasks

A total of 927 packaging task diacetyl models and 631 packaging task 2,3-pentanedione models were generated comprising 23 and 20 determinants for diacetyl and 2,3-pentanedione exposures, respectively (Table 5). Process-level factors associated with notable increases in diacetyl and 2,3-pentanedione exposure during packaging tasks were similar to those noted in the overall task model (e.g., coffee storage determinants, total number of sources, amount of coffee produced, amount of grinding performed, flavoring ground coffee, flavoring during survey). Some additional determinants associated with increased diacetyl and 2,3-pentanedione exposures included some packaging specific determinants such as open storage of coffee in the packaging area (reference: no open storage). Additionally, isolated roasting area (reference: roasting area not isolated) was associated with increased diacetyl and 2,3-pentanedione exposures during packaging tasks.

The sample-level determinants of (1) sample collected while a worker packaged flavored coffee (95% CIs: 40–55% diacetyl, 81–106% 2,3-pentanedione; reference: packaging unflavored coffee) and (2) single-serve coffee pods of ground coffee (95% CIs: 25–35% diacetyl, 4–14% 2,3-pentanedione; reference: packaging <1 lb coffee) were associated with notably higher exposures to diacetyl and 2,3-pentanedione after controlling for other covariates (Table 5; Figure 6). For context when interpreting percentage change, the intercept reference values for diacetyl and 2,3-pentanedione across all packaging models was 12.1 ppb diacetyl and 9.8 ppb 2,3-pentanedione.

The top five process-level determinants with the greatest impact on increasing diacetyl exposures during packaging tasks were (1) high (>6%) automated sources, (2) flavor ground coffee, (3) >10,000 lbs average daily roasted coffee production, (4) >2 sources of open storage of coffee, and (5) low (>0%, ≤ 6%) automated sources with 95% CIs of percent increases ranging from (1) 230–281%, (2) 140–230%, (3) 155–201%, (4)152–189%, and (5)145–179%, respectively. Similarly, the top five process-level determinants with the greatest impact on increasing 2,3-pentanedione exposures during packaging tasks were (1) flavor ground coffee, (2) isolated roasting area (reference: roasting area not isolated), (3) flavoring during survey (reference: not flavoring during survey), (4) 1–2 sources of open storage, and (5) grind flavored coffee, with 95% CIs of percent increases ranging from (1) 284–457%, (2) 231–285%, (3) 159–184%, (4) 144–192%, and (5) 101–125%, respectively (Figure 6).

Multiple process-level factors were associated with decreases in diacetyl or 2,3-pentanedione exposure during packaging tasks. Natural ventilation was associated with 95% CIs of percent decreases ranging from 16.4 to 26.5% for diacetyl (Figure 6). GEV was associated with 95% CIs of percent decreases ranging from 68.2–70.5% for diacetyl and 65.8–68.4% for 2,3-pentanedione (Figure 6). Accessory fans at the roasters and flavoring processes performed with LEV (reference: flavoring processes performed with no LEV) were associated with percent decreases ranging from 23.0–27.9% and 38.4–51.4% for 2,3-pentanedione, respectively (Figure 6).

## Discussion

Five cases of obliterative bronchiolitis observed among current and former coffee production workers were first described in 2013 and 2015 (21, 22). Since then, two recent case reports have described additional cases of obliterative bronchiolitis in workers exposed to diacetyl and 2,3-pentanedione in coffee production (12, 35) including a case of obliterative bronchiolitis observed in a current worker at one of the 17 coffee production facilities included in our study here (12). Observed cases of obliterative bronchiolitis among current and former workers in coffee production facilities along with measurements of elevated exposures to diacetyl and 2,3-pentanedione in the 17 facilities surveyed here (13) highlights a need to understand determinants of exposures to alpha-diketones in coffee production facilities such that exposure mitigation strategies can be designed and implemented accordingly.

An understanding of tasks associated with higher diacetyl and 2,3-pentanedione exposures and determinants of elevated task-based exposures is particularly important because elevated short-term exposures to diacetyl and 2,3-pentanedione can potentially contribute to (1) obliterative bronchiolitis and negative respiratory health outcomes (22, 36) and (2) higher TWA full-shift exposures (37–39). Previous studies in various workplace settings describe how specific tasks and processes contributing to high exposures can be overlooked when full-shift sampling is used to guide controls for exposure mitigation (40–42). Quantifying intermittent, task- and process-based exposures is needed for (2) an understanding of short-term or peak exposures, (1) comparison with short-term exposure limits, and/or (3) use as an exposure metric in epidemiological studies (43). Additionally, instantaneous measurements during very brief activities or at specific process-related sources can identify high exposures and emissions that could otherwise also be overlooked in exposure assessments and subsequent exposure control strategies. Further, task-based sampling can be used to develop models that identify determinants of high short-term exposures that can be directly targeted for exposure controls.

Traditional modeling approaches have favored multiple linear regression or linear mixed effects models (24, 37). However several notable limitations exist when making inferences based on these models, including uncertainty and variability associated with model building strategies and the selection of the best final model (44). Different approaches to select the final model can lead to different final models identified by different researchers. Alternatively, model averaging methods incorporate uncertainties of model selection strategies by summarizing a set of contending models to make inferences about the predictors and is widely used in other fields (45). The use of model averaging methods in occupational exposure assessment was first proposed and used by Lavoue et al. in 2009 (44). A model averaging approach is appealing because it can be implemented easily using standard statistical software (45–47). The BMA modeling approach is particularly advantageous because it addresses uncertainties in model building and variable selection, censored data issues, repeated measurements on individuals, and provides posterior distribution of parameters for all variables considered.

In our study, we identified tasks and sources associated with elevated exposures to diacetyl and 2,3-pentanedione. We then used BMA models to identify determinants associated with short-term task-based exposures to diacetyl and 2,3-pentanedione and highlight additional process-level and task-level factors to focus exposure mitigation efforts in coffee production facilities.

### Tasks and Sources Associated With Elevated Exposures to Diacetyl and 2,3-Pentanedione During Coffee Production

Grinding, flavoring, packaging ground coffee, and various production tasks with ground coffee had among the highest personal task and instantaneous activity exposures for diacetyl and 2,3-pentanedione (13). Specifically, samples collected during flavoring (50%), grinding (69%), moving roasted beans or ground coffee (50%), packaging (24%), roasting (13%), and cleaning roasting, grinding, packaging, and flavoring machines (64%) tasks exceeded the NIOSH STEL of 25 ppb diacetyl. Similarly, samples collected during flavoring (50%), grinding (34%), and packaging (6%) tasks exceeded the NIOSH STEL of 31 ppb 2,3-pentanedione. Although no exposure limits exist for instantaneous exposures to diacetyl and 2,3-pentanedione, the instantaneous measurements during specific tasks highlight opportunities for exposure mitigation controls. Ground coffee, flavored coffee, liquid flavorings, and off-gassing bins or packages were also identified as the highest sources of diacetyl and 2,3-pentanedione. Elevated task, instantaneous activity, and instantaneous source exposures associated with grinding and flavoring tasks are consistent with the work history of a coffee production worker diagnosed with obliterative bronchiolitis, who performed grinding and flavoring tasks for 7 years prior to diagnosis in the grinding area and flavoring room of one of the 17 coffee and roasting facilities described here (12).

Our results suggest coffee production facilities can consider targeting grinding and flavoring tasks for exposure mitigation. Specifically, facilities can consider isolating flavoring and grinding tasks in a designated area or room and utilizing LEV to directly remove alpha-diketone emissions from these isolated areas and processes. Facilities can also consider enclosing and ventilating grinders for reduction of alpha-diketone emissions from grinders. Another article in this Research Topic collection of articles investigating exposures and respiratory health in coffee workers shares encouraging results which show significant decreases in exposure to diacetyl and 2,3-pentanedione after enclosing and ventilating grinders. Stanton et al. observed >75% decreases in concentrations of alpha-diketones in the production space after enclosing and ventilating grinders at a large coffee production facility (48). Similar engineering controls such as enclosing and ventilating flavoring mixers can also be considered for flavoring processes (49). We also observed high exposures during moving roasted beans or ground coffee, packaging, roasting, and cleaning machines tasks, indicating additional engineering and administrative controls are needed to mitigate exposures to diacetyl and 2,3-pentanedione in coffee roasting and packaging facilities. Modeling determinants of short-term task-based exposures highlighted additional process-level and task-level factors to focus exposure mitigation efforts and are discussed in greater detail below.

### Determinants of Exposure to Diacetyl and 2,3-Pentanedione During Coffee Production Tasks

No previous studies have reported on determinants contributing to task-based exposures in coffee production, despite a need to understand factors contributing to elevated short-term exposures and design exposure mitigation strategies to reduce short-term and full-shift exposures to minimize risks for respiratory disease. The BMA models for overall task and individual tasks highlighted additional determinants that contributed to higher exposures to both diacetyl and 2,3-pentanedione including sum of all open storage sources, average percent of production as ground coffee, flavoring ground coffee and flavoring during the survey. Flavoring ground coffee was associated with 309–384% increases in diacetyl concentrations and 303–392% increases in 2,3-pentanedione compared to tasks at facilities that flavored whole bean coffee, but not ground coffee. Our results indicate a need for additional engineering controls such as LEV to capture and directly remove emissions from flavored ground coffee during flavoring processes. Additionally, sites that flavor ground coffee should evaluate where and how flavored ground coffee is stored and/or handled, to minimize further emissions during later steps in production. Similarly, any open storage of coffee was associated with elevated short-term exposures to diacetyl and 2,3-pentanedione across all tasks. Our results underscore the importance of reducing exposures across all tasks by storing all coffee in closed containers. Companies that need to store coffee in open containers as part of an off-gassing step in production can consider isolating their open containers of coffee in a space separate from other production processes and implementing source control ventilation designed to capture and remove emissions directly from the open containers. Additionally, surrogates for the amount of ground coffee produced at a site such as average percent ground coffee, total number of grinders, total grinding capacity, and average grind length in minutes were all associated with increases in exposures to diacetyl and 2,3-pentanedione across all tasks. These results highlight the importance of carefully evaluating where and how ground coffee is handled, packaged, and stored in later steps of production and implementing additional engineering and administrative controls to mitigate exposure to emissions from ground coffee.

Unsurprisingly, GEV was associated with decreases in exposures for both diacetyl and 2,3-pentanedione across all tasks, compared with exposures at sites with no GEV. Facilities that do not currently have operational GEV should consider implementing a GEV system designed to create a negative pressure in higher alpha-diketone concentration areas such as flavoring rooms or grinding areas. Pressure differentials can be created by providing GEV supply and exhaust air strategically to different production areas. For example, the exhaust air volume from the flavoring rooms or grinding areas can be designed and operated at slightly greater flow rate than the volume of supply air. A general rule is to design the GEV system with a supply flow rate set at 5–10% less than the exhaust flow rate (49, 50). GEV can also minimize the accumulation of alpha-diketones, and other potential pollutants, in the air of production spaces by diluting contaminants with supply air and exhausting contaminants to the outside (49, 51). Similarly, natural ventilation was observed to decrease diacetyl concentrations across all tasks. However, careful consideration should be taken when utilizing natural ventilation and should be done in accordance with state and local health codes. Opening doors or windows introduces unfiltered air and might contain outdoor air pollutants such as pollen and dust. Further, opening windows and doors can (1) cause imbalances in pressure differentials imparted by ventilation systems designed to mitigate exposures and (2) affect the ability of the heating, ventilation, and air-conditioning (HVAC) system to adequately control temperatures and humidity.

Interestingly, high automation and any enclosed sources were observed as determinants of increased exposure across all tasks. Because high automation and source enclosures were predominantly observed at larger coffee production sites, this observation was likely confounded by production scale. Unfortunately, we did not observe these controls at a sufficient number of small production facilities to evaluate the effect of these controls after accounting for production scale.

Additional determinants identified in the grinding model included storing ground coffee in closed containers in the grinding area which resulted in some of the largest estimated decreases in exposures, indicating that storing ground coffee in closed containers can help further mitigate exposures. Further, if the grinder was located within 10 ft of another grinder or roaster it resulted in increases in exposures during grinding tasks. This effect was markedly higher for grinders near another roaster and grinder, as compared to those near only another grinder. As discussed above, facilities can consider isolating grinding tasks in a designated area or room and increasing general ventilation as well as LEV to directly remove emissions of alpha-diketones from the isolated grinding area. Additionally, some of the highest increases in exposures during packaging tasks were observed when packaging flavored compared to unflavored coffee. Increases in exposures during packaging were also observed when packaging single serve coffee pods of ground coffee. Facilities can consider additional engineering controls to minimize sources of alpha-diketone exposure during packaging tasks such as flavored and/or ground coffee. These results underscore the importance of carefully evaluating when and how flavored or ground coffee is handled during all production steps and implementing additional engineering and administrative controls to mitigate exposures to emissions from flavored and/or ground coffee. Lastly, although exposures during roasting were among the lowest exposure tasks, there were several roasting specific determinants identified as associated with increased exposures. Some of the highest increases in exposure during roasting tasks were estimated for large, unenclosed roasters, and open storage of coffee in the roasting area. Our results suggest that enclosing roasters, especially roasters with roasting capacity >200 lbs, and storing coffee in closed containers in the roasting area can reduce exposures during roasting tasks.

Sample size for flavoring tasks was too small with too few workers observed to generate multiple linear regression models of determinants of elevated exposures while performing flavoring tasks. However, univariate analyses did identify determinants associated with increases in exposures to diacetyl and/or 2,3-pentanedione during flavoring tasks including amount of roasted coffee produced and amount of grinding performed. Similarly, the sample size for QC tasks was too small with a high degree of censoring to generate multiple linear regression models of determinants. However, univariate analyses identified QC grinding tasks, additional open storage sources, total number of sources, amount of roasted coffee produced, and amount of grinding performed as potential determinants of elevated exposure while performing QC tasks. Additionally, the highest instantaneous activity measurements of diacetyl and 2,3-pentanedione during QC tasks were observed during QC grinding.

### Differences in Determinants of Exposure to Diacetyl vs. 2,3-Pentanedione

Scatter plots of log-transformed diacetyl and 2,3-pentanedione air concentrations from task-based samples revealed a positive association with a Spearman's correlation coefficient of 0.507. A positive association was expected because both diacetyl and 2,3-pentanedione are natural byproducts from coffee roasting and are emitted during roasting, grinding, and packaging whole bean and ground coffee. However, differences in emissions of diacetyl and 2,3-pentanedione from freshly roasted coffee have been observed previously, with increased emissions rates of diacetyl, but not 2,3-pentanedione, observed with increasing roast level (52). These differences likely contributed to our observation of roast depth associated determinants (average roast length in minutes) as a notable determinant of increased exposure to diacetyl, but not 2,3-pentanedione, during roasting, packaging, and all tasks. Further, differences exist between diacetyl and 2,3-pentanedione emissions from flavored vs. non-flavored coffee because of differences in the relative amounts of diacetyl or 2,3-pentanedione added to liquid flavorings, with 2,3-pentanedione often used as a substitute for diacetyl in liquid flavorings (53). These differences in concentrations of diacetyl and 2,3-pentanedione were prominent in the task-based samples during flavoring tasks with the GM for 2,3-pentanedione being almost 9-fold higher than diacetyl. Additionally, measurements of diacetyl varied widely during flavoring tasks as indicated by a large GSD of 30.2.

Differences in concentrations observed for diacetyl and 2,3-pentanedione in processes where flavored vs. unflavored coffee was present likely contributed to differences between models for diacetyl and 2,3-pentanedione. For example, grinding flavored coffee was associated with increased exposure to 2,3-pentanedione, but not diacetyl, across all tasks. Additionally, flavoring processes performed with LEV present contributed to lower 2,3-pentanedione, but not diacetyl exposures during grinding and packaging tasks, as compared to these tasks performed at sites with no LEV. Our results suggest that source controls targeting flavoring processes not only reduce exposures during flavoring tasks but also during grinding and packaging tasks. We note that we were not able to directly assess the effect of isolating flavoring processes because an isolated flavoring room was only observed among sites that flavored ground and whole bean coffee.

### BMA Modeling Approach to Identify Determinants of Occupational Exposures

Our analyses utilized a sophisticated modeling approach (BMA) to obtain final inferences on a variety of determinants of short-term task-based exposures. Our approach allowed us to understand the (1) impact of each variable when put in models with other non-collinear variables and (2) effect of controlling for other non-collinear variables. This approach also accounted for measurements below the LOD using a Bayesian left-censored framework allowing for inferences. Our modeling strategy also reduced ambiguity present in selecting an ideal final model, because the effects of multiple models are averaged together for final inferences. We also avoided multicollinearity concerns by removing collinear combinations of variables, and subsequently generated reasonable variance estimates and the ability to identify notable differences when those were truly present. It should be noted that not all determinants will go in the models in the BMA process together, so we cannot say that all models controlled for all the other variables. Similarly, the number of models where a determinant is included is a function of how correlated that determinant is with other determinants. For example, determinants could be important but also highly correlated with many other determinants and subsequently included in relatively few models. Therefore, the number of models containing the determinant should not be interpreted as the importance of the determinant in this context. The importance of the determinant should be judged relative to slope value or the percent change. Although our results are focused on determinants of exposure in coffee roasting and packaging facilities, mixed modeling can be used to identify determinants across many occupational settings.

### Limitations and Further Research

Flavoring coffee was associated with some of the highest measurements of exposure to alpha-diketones in the surveyed coffee production facilities (13). However, our sample size for flavoring tasks was too small to generate stable models of determinants of elevated exposures while flavoring. The univariate analyses identified amount of roasted coffee produced and amount of grinding performed as potential determinants of elevated exposure while flavoring. However, the small sample size likely limited our ability to observe other potentially meaningful determinants of exposures during flavoring tasks. Further research with a sufficient sample size to generate multiple linear regression models of diacetyl and 2,3-pentanedione during flavoring tasks is needed to fully assess determinants of exposure to alpha-diketones while flavoring. Additionally, although we were able to generate stable models of determinants of elevated exposures while grinding, our sample size for short-term grinding tasks was relatively small and this contributed to small cell sizes in some categorical determinants, making it difficult to evaluate their effect. For example, small cell sizes in the no GEV category limited our ability to assess the effect of GEV on grinding task exposures. Our smaller sample size for grinding tasks was because grinding was a very brief (<2 min) task at many of the locations surveyed. Because grinding was often performed very briefly, many of the grinding tasks were sampled with instantaneous canisters, and not with short-term task-based samples, and instantaneous canister samples were not modeled in our analyses here.

Not all sample-level determinants were systematically collected during short-term task-based sample collection. Collecting the desired level of detail on exposure determinants for tasks during sampling was challenging because workers performed numerous short-lived activities and were highly mobile making their continuous observation impractical. Some sample-level determinants included unknown values due to not being recorded during short-term task-based sample collection and limited our ability to fully characterize the contributions of each sample-level determinant. Future studies including systematic collection of sample-level determinants would allow for more informed modeling of exposures during short-term task-based sample collection.

Many of our determinants were process-based and at the facility level. Because they were at the facility level, they did not vary by task and potentially directly or indirectly affected all tasks. Additionally, we did not observe determinants across all possible categories and combinations. For example, we only observed some engineering controls such as automation and enclosures at large facilities which made it difficult to evaluate the effect of these engineering controls after accounting for production scale. Future studies designed to evaluate the effect of different engineering controls such as automation and enclosing processes, specifically, are needed. Similarly, having one source near the roasters was associated with lower exposures during roasting tasks. This was an unexpected finding that is likely confounded by an isolated roasting area because we only observed an isolated roasting area at facilities with one source near the roasters. The effect of having an isolated roasting area potentially contributed to the decreased exposures during roasting tasks observed at sites with only one source near the roasters. Unfortunately, we could not evaluate the effect of sources near the roaster on exposures during roasting tasks because we did not observe having an isolated roasting area across all categories for numbers of sources near the roaster. Additional studies designed to evaluate the effect of engineering controls such as source controls, automation, enclosures, and isolation of production spaces are needed to fully assess the effects of these controls on emissions and exposures in coffee production. Our modeling results should be evaluated cautiously and should be used as a guide in decision making in combination with other facility specific information.

Further, as described in LeBouf et al., another limitation of our study is the potential for selection bias (13). Our surveys were initiated by facility management or employees through the HHE program and therefore are not a random sample of facilities. Selection bias is possible. However, the effect on exposure measurements is thought to be minimal as our measurements of exposure during specific tasks are within ranges reported in other studies (11, 14, 54).

Our analyses included task-based samples collected at coffee production facilities with fewer than 500 employees. We did not conduct surveys at large facilities, specifically those with >500 employees. Task-based exposures in larger facilities remains uncharacterized. Results from our study can alert management at larger facilities to potential tasks and sources of elevated exposure to diacetyl and 2,3-pentanedione that can be targeted for exposure mitigation; however, further research is needed to characterize short-term task-based exposures in larger facilities specifically, as their work processes, production volumes, and exposure levels could differ from those observed in our study.

### Conclusions

Grinding, flavoring, packaging, and various production tasks with ground coffee were among the highest personal task-based short-term and instantaneous activity measurements for diacetyl and 2,3-pentanedione. Ground coffee, flavored coffee, liquid flavorings, and off-gassing bins or packages were also identified as the highest sources of diacetyl and 2,3-pentanedione. Determinants associated with increased exposure to diacetyl and 2,3-pentanedione across all tasks included sum of all open storage sources and average percent of production as ground coffee. Additionally, flavoring ground coffee and flavoring during survey contributed to higher exposures for both analytes in most task groups. Our results suggest that facilities who aim to reduce exposures to alpha-diketones can consider isolating flavoring and grinding tasks in a designated area or room, adjusting these spaces to negative pressure using GEV, and enclosing and ventilating grinders and flavoring mixers with LEV to directly remove emissions of alpha-diketones from these isolated areas and processes. Additionally, facilities can consider minimizing open storage of roasted coffee, with special attention given to the open storage and off-gassing of ground coffee. Additional LEV can be used to directly remove alpha-diketones from off-gassing coffee where open storage is required for off-gassing procedures and can mitigate exposures near stored coffee as well as throughout the facility.

## Data Availability Statement

The datasets presented in this article are not readily available because of restrictions imposed under the US Privacy Act and the limitations of what participants consented to. The data underlying the analyses presented, beyond what is provided in the paper, are confidential and not available to researchers outside the National Institute for Occupational Safety and Health (NIOSH). For more information about NIOSH's policy regarding sensitive data, see https://www.cdc.gov/niosh/ocas/datahandle.html. Requests to access the datasets should be directed to BB, bblackley@cdc.gov.

## Ethics Statement

The studies involving human participants were reviewed and approved by the NIOSH Institutional Review Board (NIOSH Protocol 17-RHD-06XP). The patients/participants provided their written informed consent to participate in this study.

## Author Contributions

BB, JC-G, AF, RL, and MV contributed to conception and design of the study. BB, CG, XL, and MV performed the statistical analyses. BB wrote the first draft of the manuscript. All authors contributed to manuscript revisions, read, and approved the submitted version.

## Funding

This work was supported by the National Institute for Occupational Safety and Health (NIOSH).

## Conflict of Interest

The authors declare that the research was conducted in the absence of any commercial or financial relationships that could be construed as a potential conflict of interest.

## Publisher's Note

All claims expressed in this article are solely those of the authors and do not necessarily represent those of their affiliated organizations, or those of the publisher, the editors and the reviewers. Any product that may be evaluated in this article, or claim that may be made by its manufacturer, is not guaranteed or endorsed by the publisher.
