# Supplementary material for: Determinants of Task-Based Exposures to Alpha-Diketones in Coffee Roasting and Packaging Facilities Using a Bayesian Model Averaging Approach
Source: Front Public Health. 2022 Jun 9;10:878907. doi: 10.3389/fpubh.2022.878907 (PMC9218577; doi:10.3389/fpubh.2022.878907)

**Supplementary Materials:**

**Methods:**

**Descriptive Analysis of Instantaneous Activity and Source Based Samples**

Instantaneous canister samples were summarized for various personal or source activities. Geometric means (GMs), geometric standard deviations (GSDs), maximums, and 95^th^ percentiles were developed using traditional lognormal likelihood-based formulas when all data were above the LOD. When measurements were below the LOD, a Bayesian intercept only (ANOVA; no predictors) model was used to estimate statistics. Due to few workers with repeated measurements, a fixed effect model without random effects for subjects was used. We used 20,000 iterations after 5,000 iterations of burn-in. For further description of this Bayesian model see the fixed-effect model described in LeBouf et al. (13).

**Task-based Univariate Determinant Models**

Determinants of short-term task-based exposure to diacetyl or 2,3-pentanedione were created using indicators (0/1) for categorical variables or using continuous measures. Continuous measures were used when > 5 levels were present in a continuous measure, otherwise categories were developed. Categories with less than 4 observations were collapsed. Continuous determinants were centered for the final BMA approach to promote stability in the models.

We performed a series of single-variable Bayesian linear regression models with each individual determinant separately to identify important determinants of exposures. Categorical variables were compared with the reference category. The reference category for engineering control variables was “no” or “none.” The reference category for variables related to source strength were categories hypothesized to have the weakest source strength and therefore lowest exposure. From these models, we obtained estimates of the regression coefficients and GMs. Determinants were designated as notable if the 80% credible interval (Bayesian uncertainty interval) for the regression estimate of any slope did not include 0.

**Results:**

**Instantaneous Activity-based and Source Exposure Summary**

A summary of instantaneous activity-based and source samples can be seen in Supplementary Tables 2–3. Below, we provide a summary of instantaneous activity-based and source exposures by task and process type. We note many of the task activities and sources are based on small sample sizes and should be interpreted with caution.

*Flavoring Activities and Sources*

Among all flavoring tasks sampled with instantaneous canisters, flavoring ground coffee had the highest activity-based levels for diacetyl (maximum=2,307 ppb) and 2,3-pentanedione (maximum=1,509 ppb) (Supplementary Table 2). Instantaneous source measurements for flavoring related sources indicated high concentrations of diacetyl and 2,3-pentanedione at the dispenser of flavoring in the flavoring hopper (18,744 ppb diacetyl and 9,746 ppb 2,3-pentanedione based on one sample) and above the flavoring mixer when flavoring ground coffee (6,732 ppb diacetyl and 236 ppb 2,3-pentanedione based on one sample) (Supplementary Table 3). Several instantaneous flavoring related source samples had no detectable diacetyl (all<LOD) but high levels of 2,3-pentanedione (1,469–29,219 ppb) (Supplementary Table 3).

*Grinding Activities and Sources*

Instantaneous grinding activities and ground coffee related source samples varied widely as noted by large GSDs (Supplementary Tables 2-3). Diacetyl and 2.3-pentadione exposures associated with grinding sources were orders of magnitude higher than grinding task activities (Supplementary Table 2). Among all grinding tasks sampled, grinding 2 lbs (1 sample=8,524 ppb), grinding 6 lbs (P95=1,230 ppb), and dumping whole beans into grinder (maximum=370 ppb) had the highest levels for diacetyl whereas grinding 2 lbs (1 sample=4,693 ppb), grinding 6 lbs coffee (P95=1,161 ppb), and grinding 40 lbs flavored coffee (maximum=212 ppb) had the highest levels for 2,3-pentanedione (Supplementary Table 2). High P95 estimates were noted for instantaneous source samples collected above a bag or bin of ground coffee (P95=238,480 ppb diacetyl and 17,502 ppb 2,3-pentanedione), at production grinders’ discharge (P95=45,517 ppb diacetyl and 25,004 ppb 2,3-pentanedione), and at sources associated with production grinding (P95=13,867 ppb diacetyl and 7,299 ppb 2,3-pentanedione) (Supplementary Table 3).

*Packaging Activities and Sources*

The highest instantaneous activity sample during packaging tasks was collected while a worker dispensed ground coffee into a hopper for packaging (3,765 ppb diacetyl and 3,377 ppb 2,3-pentanedione based on 1 sample) (Supplementary Table 2). Similarly, the highest instantaneous packaging source samples were collected at single serve pods with ground coffee packaging machines (P95=508 ppb diacetyl and 826 ppb 2,3-pentanedione) (Supplementary Table 3). Packaging ground coffee was among the highest source samples as well (P95=260 ppb diacetyl and 180 ppb 2,3-pentanedione) (Supplementary Table 3).

*Other Production Activities and Sources*

Other instantaneous activity and source samples with high levels of diacetyl and 2,3-pentanedione included miscellaneous production tasks with ground coffee, coffee storage and off-gassing sources, and moving roasted beans. High instantaneous activity measurements during miscellaneous production tasks with ground coffee were collected while a worker removed a supersack from ground coffee (maximums=2,692 ppb diacetyl and 949 ppb 2,3-pentanedione) (Supplementary Table 2). High instantaneous source measurements were collected above bins or packages in the off-gassing area (maximums=10,541 ppb and 4,123 ppb 2,3-pentanedione), degassing a coffee package (4,322 ppb diacetyl and 1,389 ppb 2,3-pentanedione based on one sample), just above a bin while the bin was opened (maximums=7,386 ppb diacetyl and 1,749 ppb 2,3-pentanedione) and from inside a whole bean storage bin (P95= 6,956 ppb diacetyl and 4,624 ppb 2,3-pentanedione) (Supplementary Table 3). Moving roasted beans activities and sources varied widely in measurements of diacetyl and 2,3-pentanedione, with the highest samples of any moving roasted beans activities measured while scooping beans from a storage container (P95=407 ppb diacetyl and 433 ppb 2,3-pentanedione) (Supplementary Table 2). Similarly, the highest samples collected at any moving roasted beans related sources were measured while scooping beans from a storage container (215 ppb diacetyl and 108 ppb 2,3-pentanedione based on one sample) (Supplementary Table 3).

**Univariate Analyses of Short-term TWA Task-based Exposures**

*Roasting Tasks*

Sample level determinants associated with notable increases (p<0.2) in diacetyl and 2,3-pentanedione exposure during roasting tasks included sampled roaster capacity (in lbs) and whether it was enclosed, as well as whether the sampled roaster was near (within 10 feet) of another grinder and roaster (Figures 2 and 3; Supplementary Table 4).

Process-level determinants associated with notable increases (p<0.2) in diacetyl and 2,3-pentanedione exposure during roasting tasks included coffee storage determinants such as sum of all open storage sources (≥1 open storage source) and open storage in roasting area versus elsewhere (Figures 2 and 3; Supplementary Tables 5 and 6). General sources determinants such as total number of sources (>7 sources) and number of other sources near roasters (>1 source) were associated with notable increases as well (Figures 2 and 3; Supplementary Tables 5 and 6).

Total production capacity and type of coffee produced, such as daily production amount and capacities, amount of grinding, and flavoring process determinants, were associated with notable increases in diacetyl and 2,3-pentanedione as well. Specifically, amount of roasted coffee produced (average roasted coffee production (≥10,000 lbs/day), total number of roasters (≥2), total roaster capacity (lbs), total number of package lines) and amount of grinding performed (average percent of production as ground coffee, average grind length (mins), total number of grinders, total grinding capacity (lbs)) were also associated with notable increases in roasting task exposures to diacetyl and 2,3-pentanedione. Flavoring during the survey was also associated with notable increases in diacetyl and 2,3-pentanedione during roasting tasks. Flavoring ground coffee (and having an isolated flavoring room; all facilities that flavored ground coffee had an isolated flavoring room), as well as average roast length in minutes (≥ 15 minutes) was associated with notable increases in diacetyl but not 2,3-pentanedione exposures (Figures 2 and 3; Supplementary Tables 5 and 6).

Engineering controls such as general exhaust ventilation (GEV) was associated with notable decreases in exposure to diacetyl and 2,3-pentanedione (Figures 2 and 3; Supplementary Tables 5 and 6). Natural ventilation was also associated with notable decreases in exposure to diacetyl during roasting tasks (Figure 2; Supplementary Table 5). Determinants associated with notable increases in exposure to diacetyl and 2,3-pentanedione during roasting tasks included percent automated sources (low (≤ 6%) and high (> 6%) percent automated sources versus none), any isolated sources/processes, and any enclosed sources (Figures 2 and 3; Supplementary Tables 5 and 6).

*Grinding Tasks*

Sample level determinants associated with notable increases (p<0.2) in diacetyl and 2,3-pentanedione exposure during grinding tasks included whether the sampled grinder was near (within 10 feet) of another grinder and roaster (Figures 2 and 3; Supplementary Table 4).

Process-level determinants associated with notable increases (p<0.2) in diacetyl and 2,3-pentanedione exposure during grinding tasks included coffee storage determinants such as sum of all open storage sources (≥1 open storage source) and if ground coffee in the grinding area was not stored in containers with lids (open containers, packaging, permeable supersack, and silo) (Figures 2 and 3; Supplementary Tables 5 and 6). General sources determinants such as total number of sources (>7 sources) were associated with notable increases in diacetyl and 2,3-pentanedione and number of other sources near grinders (4–5 sources within 10 feet of grinders) was associated with notable increases in diacetyl during grinding tasks (Figures 2 and 3; Supplementary Tables 5 and 6).

Similar to roasting tasks, total production capacity and type of coffee produced, such as daily production amount and capacities, amount of grinding, and flavoring process determinants, were associated with notable increases in exposure to diacetyl and 2,3-pentanedione during grinding tasks as well. Specifically, amount of roasted coffee produced (average roasted coffee production (≥10,000 lbs/day), total number of roasters (≥2), total roaster capacity (lbs), total number of package lines) and amount of grinding performed (average percent of production as ground coffee, average grind length (mins), total number of grinders, total grinding capacity (lbs)) were also associated with notable increases in grinding task exposures to diacetyl and 2,3-pentanedione. Flavoring ground coffee and having an isolated flavoring room was associated with notable increases in diacetyl and 2,3-pentanedione exposures (Figures 2 and 3; Supplementary Tables 5 and 6).

Engineering controls associated with notable decreases in exposure to 2,3-pentanedione included flavoring ventilation (local exhaust ventilation (LEV) versus no LEV) (Figure 2; Supplementary Table 1). Determinants associated with notable increases in exposure to diacetyl and 2,3-pentanedione during grinding tasks included percent automated sources (low (≤ 6%) and high (> 6%) percent automated sources versus none), and isolated roasting area (Figures 2 and 3; Supplementary Tables 5 and 6).

*Packaging Tasks*

Sample level determinants associated with notable increases (p<0.2) in diacetyl and 2,3-pentanedione exposure during packaging tasks included sampled packaging volume (single serve) and sampled while packaging flavored coffee (Figures 2 and 3; Supplementary Table 4).

Process-level determinants associated with notable increases (p<0.2) in diacetyl and 2,3-pentanedione exposure during packaging tasks included coffee storage determinants such as sum of all open storage sources (≥1 open storage source) and open storage in packaging area versus elsewhere (Figures 2 and 3; Supplementary Tables 5 and 6). Total number of sources (>7 sources) were associated with notable increases as well.

Amount of roasted coffee produced (average roasted coffee production (≥10,000 lbs/day), total roaster capacity (lbs), total number of package lines) and amount of grinding performed (average percent of production as ground coffee, average grind length (mins), total number of grinders) were also associated with notable increases in packaging task exposures to diacetyl and 2,3-pentanedione. Flavoring during the survey as well as flavoring ground coffee (and having an isolated flavoring room) were associated with notable increases in diacetyl and 2,3-pentanedione exposures during packaging tasks as well. Average roast length in minutes (≥ 15 minutes) was associated with notable increases in diacetyl but not 2,3-pentanedione exposures during packaging tasks (Figures 2 and 3; Supplementary Tables 5 and 6).

Engineering controls such as GEV were associated with notable decreases in exposure to diacetyl and 2,3-pentanedione (Figures 2 and 3; Supplementary Tables 3 and 4). Accessory fans at the roasters as well as flavoring ventilation (LEV) were also associated with notable decreases in exposure to 2,3-pentanedione during packaging tasks (Figure 2; Supplementary Table 1). Determinants associated with notable increases in exposure to diacetyl and 2,3-pentanedione during packaging tasks included percent automated sources (low (≤ 6%) and high (> 6%) percent automated sources versus none), any isolated sources/processes, isolated roasting area, and any enclosed sources (Figures 2 and 3; Supplementary Tables 5 and 6).

*Quality Control Tasks*

Quality control task type was associated with notable increases (p<0.2) in 2,3-pentanedione, but not diacetyl exposure during quality control tasks (Supplementary Table 7). Specifically, quality control grinding tasks were associated with notably higher exposures to 2,3-pentanedione when compared with quality control roasting tasks.

Process-level determinants associated with notable increases (p<0.2) in diacetyl and 2,3-pentanedione exposure during quality control tasks included coffee storage determinants (sum of all open storage sources) and total number of sources (>7 sources). Additionally, amount of roasted coffee produced (average roasted coffee production (>1,000 lbs/day), total roaster capacity (lbs), total number of package lines (≥4 lines)) and amount of grinding performed (average percent of production as ground coffee, average grind length (mins), total number of grinders, and total grinding capacity (lbs)) were also associated with notable increases in quality control task exposures to diacetyl and 2,3-pentanedione. Average roast length in minutes (≥ 15 minutes) was associated with notable decreases in diacetyl and 2,3-pentanedione exposures during quality control tasks; however, we note the small number of observations (n=4) in the longer duration average roast length category (Supplementary Table 7).

Engineering controls such as GEV, natural ventilation, and accessory fans at the roasters were associated with notable decreases in exposure to diacetyl and 2,3-pentanedione (Supplementary Table 7). Determinants associated with notable increases in exposure to diacetyl and 2,3-pentanedione during quality control tasks included percent automated sources (low (≤ 6%) and high (> 6%) percent automated sources versus none), and any enclosed sources (Supplementary Table 7).

*Flavoring Tasks*

No sample level determinants were systematically collected for flavoring tasks. Process-level determinants associated with notable increases (p<0.2) in diacetyl exposure during flavoring tasks included amount of roasted coffee produced (average roasted coffee production (>1,000 lbs/day), and total number of roasters. Notable increases in diacetyl and 2,3-pentanedione exposure during flavoring tasks were observed with increases in total roaster capacity (lbs) and total number of package lines. Amount of grinding performed (average grind length (mins), and total grinding capacity) were also associated with notable increases in flavoring task exposures to diacetyl. Total number of grinders was associated with notable increases in 2,3-pentanedione exposures during flavoring tasks as well (Supplementary Table 7).

Engineering controls such as natural ventilation and accessory fans at the roasters were associated with decreases in exposure to diacetyl and 2,3-pentanedione (Supplementary Table 4). Determinants associated with notable increases in exposure to diacetyl and 2,3-pentanedione during flavoring tasks included percent automated sources (low (≤ 6%) and high (> 6%) percent automated sources versus none), and any enclosed sources (Supplementary Table 4). Any isolated sources/processes, and having an isolated flavoring room specifically, were associated with notable increases in exposure to diacetyl during flavoring tasks as well (Supplementary Table 5).

*Overall Tasks*

Sample level determinants associated with notable increases (p<0.2) in diacetyl and 2,3-pentanedione exposure during all tasks included task type (grinding, flavoring, packaging, moving roasted beans or ground coffee, and QC tasks versus roasting tasks) (Figures 2 and 3; Supplementary Table 2).

Process-level determinants associated with notable increases (p<0.2) in diacetyl and 2,3-pentanedione exposure across all tasks included coffee storage determinants such as sum of all open storage sources (≥1 open storage source). Total number of sources (>7 sources) were associated with notable increases as well.

Amount of roasted coffee produced (average roasted coffee production (≥10,000 lbs/day), total number of roasters (≥2), total roaster capacity (lbs), total number of package lines) and amount of grinding performed (average percent of production as ground coffee, average grind length (mins), total number of grinders, total grinding capacity in lbs) were also associated with notable increases in overall task exposures to diacetyl and 2,3-pentanedione. If a facility ground flavored coffee was associated with higher 2,3-pentanedione, but not diacetyl, exposures during all tasks (Figures 2 and 3; Supplementary Tables 3 and 4).

Flavoring during the survey as well as flavoring ground coffee (and having an isolated flavoring room) were associated with notable increases in diacetyl and 2,3-pentanedione exposures during all tasks as well. Average roast length in minutes (≥ 15 minutes) was associated with notable increases in diacetyl but not 2,3-pentanedione exposures during packaging tasks (Figures 2 and 3; Supplementary Tables 3 and 4).

Engineering controls such as GEV were associated with notable decreases in exposure to diacetyl and 2,3-pentanedione (Figures 2 and 3; Supplementary Tables 3 and 4). Natural ventilation was associated with lower diacetyl, but not 2,3-pentanedione exposures during all tasks. Conversely, accessory fans at the roasters were associated with notable decreases in exposure to 2,3-pentanedione, but not diacetyl, during all tasks (Figure 2; Supplementary Table 1). Determinants associated with notable increases in exposure to diacetyl and 2,3-pentanedione during all tasks included percent automated sources (low (≤ 6%) and high (> 6%) percent automated sources versus none), any isolated sources/processes, and any enclosed sources (Figures 2 and 3; Supplementary Tables 3 and 4).

**Supplementary Tables:**

**Supplementary Table 1: Descriptions and source-receptor model component for sample-level and process-level determinants**

| **Determinant** | **Description** | **Source-receptor model component** |
| --- | --- | --- |
| **Sample-level, Roasting tasks** | | |
| **Sampled Roaster Capacity in lbs and Enclosed** | Sample roasted capacity in lbs and enclosure status | Source (activity emission) and process enclosure |
| Small capacity (< 200), no enclosure (intercept) |  |  |
| Large capacity (≥ 200), no enclosure |  |  |
| Large capacity (≥ 200), enclosed |  |  |
| **Sampled roaster near grinder or near roaster** | Sampled roaster within 10 ft of any grinders and/or roasters | Nearby processes (grinders or roasters within 10 ft) |
| No nearby grinder or roaster (intercept) |  |  |
| Nearby roaster, no nearby grinder |  |  |
| Nearby grinder, no nearby roaster |  |  |
| Nearby grinder and roaster |  |  |
| **Sample-level, Grinding tasks** | | |
| **Sampled grinder, typical weight grind** | Typical amount of coffee ground when using sampled grinder | Source (activity emission) |
| Small (intercept)  (≤ 5 lbs) |  |  |
| Large (> 5 lbs) |  |  |
| **Sampled grinder near another grinder or roaster** | Sampled grinder within 10 ft of any grinders and/or roasters | Nearby sources (grinders or roasters within 10 ft) |
| Not near grinder or roaster (intercept) |  |  |
| Near grinder, not near roaster |  |  |
| Near grinder and roaster |  |  |
| **Sampled grinder enclosed and automated** | Sampled grinder enclosed and automated (yes) or sampled grinder unenclosed and manual (no) | Process enclosure |
| No (intercept) |  |  |
| Yes |  |  |
| **Sampled while grinding flavored coffee** | Sample collected while grinding flavored coffee | Source (activity emission) |
| No (intercept) |  |  |
| Yes |  |  |
| **Sample-level, Packaging tasks** | | |
| **Sampled packaging volume** | Amount of coffee packaged during sample collection | Source (activity emission) |
| < 1 lb (intercept) |  |  |
| 1─5 lbs |  |  |
| 5 lb |  |  |
| Single serve |  |  |
| Unknown < 5 lbs |  |  |
| **Sampled while packaging ground coffee** | Sample collected while packaging ground coffee | Source (activity emission) |
| No (intercept) |  |  |
| Yes |  |  |
| Unknown |  |  |
| **Sampled while packaging flavored coffee** | Sample collected while packaging flavored coffee | Source (activity emission) |
| No (intercept) |  |  |
| Yes |  |  |
| Unknown |  |  |
| **Sample-level, Quality control tasks** | | |
| **Sampled task** | Quality control task performed during sample collection | Source (activity emission) |
| Quality control roasting |  |  |
| Brewing, cupping, and tasting |  |  |
| Quality control grinding |  |  |
| **Sample-level, Overall tasks** | | |
| **Sampled task** | Task performed during sample collection | Source (activity emission) |
| Roasting (intercept) |  |  |
| Quality control (QC) |  |  |
| Packaging coffee |  |  |
| Moving roasted beans or ground coffee |  |  |
| Miscellaneous production |  |  |
| Grinding coffee beans |  |  |
| Flavoring coffee |  |  |
| Cleaning machines |  |  |
| **Process-level, Coffee Storage Determinants** | | |
| **Sum of all open storage sources** | Sum of open storage of coffee (whole bean or ground coffee) in each production space  (sum of: open storage in roasting + open storage in grinding + open storage in packaging + open storage in flavoring) | Source (activity emission) |
| None (intercept) |  |  |
| Small (1-2) |  |  |
| Large (> 2) |  |  |
| **Open storage in task area versus elsewhere** | Open storage in task area, open storage elsewhere, or no open storage | Source (activity emission) |
| No open storage (intercept) |  |  |
| Open storage in task area |  |  |
| Open storage elsewhere |  |  |
| **Ground coffee storage container in grinding area** | Type of containers used for storage of ground coffee in grinding area | Source enclosure |
| Open container (intercept) |  |  |
| Containers with lids |  |  |
| Packaging |  |  |
| Permeable supersack |  |  |
| Silo |  |  |
| **Process-Level, General Sources Determinants** | | |
| **Total number of sources** | Sum of total number of sources  (Sum of number of roasters + number of grinders + number of packaging lines + sum of all open storage + flavoring during survey) | Source (activity emission) |
| Small (intercept) (≤ 7) |  |  |
| Large (> 7) |  |  |
| **Number of other sources near roasters** | Number of sources within 10 ft of roasters | Nearby sources |
| None |  |  |
| 1 |  |  |
| > 1 |  |  |
| **Number of other sources near grinders** | Number of sources within 10 ft of grinders | Nearby sources |
| Intercept |  |  |
| Slope |  |  |
| **Process-level, Process Determinants** | | |
| **Amount of roasted coffee produced** | | |
| **Average roasted coffee production, lbs per day** | Average roasted coffee production in lbs per day | Source (activity emission) |
| Small (intercept)  (< 1,000) |  |  |
| Medium  (≥ 1,000 and < 10,000) |  |  |
| Large  **(≥** 10,000) |  |  |
| **Total number of roasters** | Total number of roasters | Source (activity emission) |
| 1 (intercept) |  |  |
| 2─3 |  |  |
| **Total roaster capacity in lbs** | Roaster 1 capacity + roaster 2 capacity + roaster 3 capacity | Source (activity emission) |
| Intercept |  |  |
| Slope |  |  |
| **Total number of package lines** | Total number of packaging lines | Source (activity emission) |
| Intercept |  |  |
| Slope |  |  |
| **Type of roasted coffee produced (roast depth)** | | |
| **Average roast length in minutes** | Average roast length in minutes | Source (activity emission) |
| < 15 minutes |  |  |
| ≥ 15 minutes |  |  |
| **Amount of grinding performed and grinding process determinants** | | |
| **Average percent ground coffee of total production** | Average amount of ground coffee (as percent of total production) | Source (activity emission) |
| Intercept |  |  |
| Slope |  |  |
| **Average grind length in mins** | Average grinding task length, in minutes | Source (activity emission) |
| Intercept |  |  |
| Slope |  |  |
| **Total number of grinders** | Total number of grinders | Source (activity emission) |
| Intercept |  |  |
| Slope |  |  |
| **Total grinding capacity in lbs** | Sum of all grinders’ capacities | Source (activity emission) |
| Intercept |  |  |
| Slope |  |  |
| **Grind flavored coffee** | Facility grinds flavored coffee | Source (activity emission) |
| No |  |  |
| Yes |  |  |
| **Flavoring-related processes** | | |
| **Flavor (and package) ground coffee, accessory fans in flavoring room, and isolated flavoring room** | Facility flavors ground coffee and has accessory fans in isolated flavoring room* | Source (activity emission), fans present, and process isolation |
| Flavor (and package) whole bean coffee, do not flavor ground coffee/no accessory fans/no isolated flavoring room |  |  |
| Flavor (and package) ground and whole bean coffee/accessory fans/isolated flavoring room |  |  |
| Do not flavor coffee |  |  |
| **Flavoring during survey** | Flavoring was performed during the exposure assessment survey | Source (activity emission) |
| No flavoring (intercept) |  |  |
| Flavoring |  |  |
| **Process-level, Engineering Controls Determinants** | | |
| **Percent automated sources** | Percent of total number of sources that were automated | Automation |
| None (intercept) |  |  |
| Low (≤ 6) |  |  |
| High (> 6) |  |  |
| **Any isolated sources/processes** | Any sources or processes were isolated | Isolation |
| No (intercept) |  |  |
| Yes |  |  |
| **Any enclosed sources** | Any sources were enclosed | Process enclosure |
| No (intercept) |  |  |
| Yes |  |  |
| **GEV (with mechanical supply)** | Facility had GEV, with mechanical supply air | Ventilation |
| No (intercept) |  |  |
| Yes |  |  |
| **Natural ventilation** | Open windows or open doors during the survey | Ventilation |
| No (intercept) |  |  |
| Yes |  |  |
| **Accessory fans at the roasters** | Accessory fans were present at the roasters | Fans present |
| No (intercept) |  |  |
| Yes |  |  |
| **Isolated roasting area** | Roasting area was isolated | Isolation |
| No (intercept) |  |  |
| Yes |  |  |
| **Flavoring Ventilation (LEV)** | LEV is present during flavoring processes | Source Control |
| Flavoring, no LEV |  |  |
| Flavoring, LEV |  |  |
| No flavoring during survey |  |  |

*****all sites that flavored ground coffee also had an isolated flavoring room and accessory fans in the flavoring room

**Supplementary Table 2. Instantaneous activity task-based measurements of exposure to diacetyl and 2,3-pentanedione**

| **Task Group** | **N** | **Diacetyl** | | | | **2,3-Pentanedione** | | | |
| --- | --- | --- | --- | --- | --- | --- | --- | --- | --- |
|  |  | **GM (ppb)** | **GSD** | **P95 (ppb)** | **%BDL** | **GM (ppb)** | **GSD** | **P95 (ppb)** | **%BDL** |
| **Flavoring coffee** | | | | | | | | | |
| **Flavoring ground coffee** | 2 | – | – | 2,307* | 0 | – | – | 1,509* | 0 |
| **Flavoring unspecified if whole or ground** | 4 | – | – | 1,869* | 0 | – | – | 1,433* | 0 |
| **Above flavoring mixer with flavored whole bean coffee** | 5 | 65.6 | 6.7 | 1,500 | 0 | 84.9 | 5.1 | 1,259 | 0 |
| **Flavoring whole bean** | 2 | – | – | 37.6* | 0 | – | – | 38.5* | 0 |
| **Mixing/weighing flavors** | 3 | – | – | 509* | 33 | – | – | 13.1* | 0 |
| **Grinding flavored coffee** | | | | | | | | | |
| **Grinding 40 lbs flavored coffee** | 4 | – | – | 77.7* | 50 | – | – | 212* | 0 |
| **Grinding unflavored coffee** | | | | | | | | | |
| **Grinding 2 lbs** | 1 | 8,524† | – | – | 0 | 4,693† | – | – | 0 |
| **Grinding 6 lb bags** | 7 | 70.4 | 5.7 | 1,230 | 0 | 63.0 | 5.9 | 1,161 | 0 |
| **Dumping whole beans into grinder** | 4 | – | – | 370* | 0 | – | – | 131* | 0 |
| **Grinding 12 oz bags** | 1 | 30.8† | – | – | 0 | 28.6† | – | – | 0 |
| **Grinding (unspecified volume)** | 27 | 20.5 | 2.4 | 89.0 | 0 | 11.2 | 3.1 | 73.7 | 4 |
| **Grinding 5 lb bags** | 8 | 12.7 | 2.7 | 65.5 | 0 | 9.0 | 3.0 | 53.5 | 0 |
| **QC Grinding** | | | | | | | | | |
| **Grinding for QC cupping** | 7 | 39.0 | 1.7 | 90.4 | 0 | 25.7 | 1.8 | 67.2 | 0 |
| **Grinding for QC of roast depth (at roaster)** | 30 | 25.5 | 3.8 | 227 | 0 | 15.5 | 3.5 | 123 | 0 |
| **QC grinding** | 2 | – | – | 29.4* | 0 | – | – | 20.8* | 0 |
| **Moving roasted beans** | | | | | | | | | |
| **Scooping beans from storage container** | 6 | 38.4 | 4.2 | 407 | 0 | 32.0 | 4.9 | 433 | 0 |
| **Dumping roasted beans into hopper/storage bin/destoner** | 12 | 9.5 | 11.0 | 482 | 8 | 5.4 | 9.7 | 223 | 17 |
| **Transfer of roasted beans** | 2 | – | – | 59.6* | 0 | – | – | 25.1* | 0 |
| **Getting beans from storage bins** | 1 | 5.5† | – | – | 0 | 4.0† | – | – | 0 |
| **Moving beans from storage bin into destoner** | 1 | 2.0† | – | – | 0 | <LOD | – | – | 100 |
| **Cleaning machines** | | | | | | | | | |
| **Cleaning packaging machine** | 2 | – | – | 123.7* | 0 | – | – | 37.1* | 0 |
| **Cleaning chaff out of roaster exhaust line** | 6 | 11.2 | 1.3 | 18.2 | 0 | 8.0 | 1.7 | 19.5 | 0 |
| **Packaging** | | | | | | | | | |
| **Dispensing ground coffee into hopper for packaging** | 1 | 3,765† | – | – | 0 | 3,377† | – | – | 0 |
| **At autosealer** | 1 | 1,279† | – | – | 0 | 408† | – | – | 0 |
| **Packing bags into box** | 3 | – | – | 82.3* | 0 | – | – | 45.4* | 0 |
| **Filling packaging dispenser using vacuum** | 9 | 37.0 | 1.3 | 60.4 | 0 | 29.0 | 1.4 | 51.4 | 0 |
| **Packaging whole bean coffee** | 2 | – | – | 69.7* | 0 | – | – | 80.5* | 0 |
| **Packaging ground coffee** | 5 | 18.0 | 2.0 | 55.1 | 0 | 11.5 | 2.1 | 39.0 | 0 |
| **Packaging coffee (unspecified)** | 7 | 9.7 | 6.6 | 218 | 0 | 6.0 | 5.9 | 111 | 0 |
| **Filling packages with roasted coffee at packaging machine** | 6 | 28.8 | 1.7 | 67.9 | 0 | 24.3 | 1.6 | 53.3 | 0 |
| **Dumping beans into packaging machine** | 1 | 6.3† | – | – | 0 | 3.3† | – | – | 0 |
| **Miscellaneous production** | | | | | | | | | |
| **Removing supersack from ground coffee** | 2 | – | – | 2,692* | 0 | – | – | 949* | 0 |
| **Hand mixing of ground coffee** | 1 | 148† | – | – | 0 | 94.7† | – | – | 0 |
| **Rework ground coffee** | 1 | 27.6† | – | – | 0 | 14.6† | – | – | 0 |
| **Hand blending roasted beans** | 11 | 18.1 | 2.6 | 88.5 | 0 | 12.5 | 2.7 | 65.0 | 0 |
| **Opening a roasted bean storage bin** | 1 | 14.6† | – | – | 0 | 12.9† | – | – | 0 |
| **Weighing roasted beans** | 4 | – | – | 7.2* | 0 | – | – | 4.1* | 0 |
| **Roasting coffee beans** | | | | | | | | | |
| **Roaster taking sample of roasted beans from cooling bin** | 16 | 19.1 | 3.4 | 142 | 6 | 9.5 | 4.5 | 111 | 13 |
| **Roasting** | 5 | 10.1 | 2.6 | 48.5 | 0 | 0.9 | 71.3 | 30.6* | 40 |
| **Dumping green beans into hopper or roaster** | 1 | 26.3† | – | – | 0 | 19.6† | – | – | 0 |
| **Dumping roasted beans into cooling bin or working at cooling bin** | 37 | 9.3 | 5.2 | 141 | 5 | 6.2 | 4.6 | 77.1 | 8 |
| **Roaster checking color and/or smell of roasting beans** | 11 | 8.2 | 3.7 | 70.7 | 0 | 4.4 | 3.6 | 35.9 | 9 |
| **Dispensing beans from cooling bin into storage bin** | 12 | 2.1 | 4.8 | 26.8 | 8 | 0.8 | 14.2 | 55.0 | 50 |
| **QC** | | | | | | | | | |
| **Cupping** | 7 | 19.0 | 2.3 | 72.8 | 0 | 13.8 | 2.9 | 80.0 | 0 |
| **Misc QC** | 5 | 21.8 | 1.6 | 46.9 | 0 | 12.7 | 1.4 | 22.8 | 0 |

N=number of samples; GM=geometric mean; ppb=parts per billion; GSD=geometric standard deviation; P95=95^th^ percentile; %BDL=percent samples below the limit of detection; *indicates where maximum is presented when <5 measurements were above the detection limit; †indicates where raw value is presented when N=1; ─indicates not enough samples above the detection limit to obtain an estimate

**Supplementary Table 3. Instantaneous source measurements of diacetyl and 2,3-pentanedione**

| **Source** | **N** | **Diacetyl** | | | | **2,3-Pentanedione (ppb)** | | | |
| --- | --- | --- | --- | --- | --- | --- | --- | --- | --- |
|  |  | **GM**  **(ppb)** | **GSD** | **P95**  **(ppb)** | **%BDL** | **GM**  **(ppb)** | **GSD** | **P95**  **(ppb)** | **%<BDL** |
| **Flavoring related sources** | | | | | | | | | |
| **At dispenser of flavoring in flavoring hopper** | 1 | 18,744† | – | – | 0 | 9,746† | – | – | 0 |
| **Above flavoring mixer with flavored ground coffee** | 1 | 6,732† | – | – | 0 | 236† | – | – | 0 |
| **Above flavoring mixer with flavored whole bean coffee** | 4 | – | – | 420* | 50 | – | – | 80,496* | 25 |
| **Above empty flavoring hopper** | 3 | – | – | 28,399 | 0 | – | – | 21,899 | 0 |
| **At discharge from blender** | 1 | 86.7† | – | – | 0 | 69.9† | – | – | 0 |
| **Flavoring whole bean** | 1 | 27.5† | – | – | 0 | 68.6† | – | – | 0 |
| **Flavoring headspace** | 1 | <LOD | – | – | 100 | 1,469† | – | – | 0 |
| **Over bag while adding flavoring** | 1 | <LOD | – | – | 100 | 29,219† | – | – | 0 |
|  |  |  |  |  |  |  |  |  |  |
| **Grinding/ground coffee related sources** | | | | | | | | | |
| **Rework ground coffee** | 2 | – | – | 5,973* | 0 | – | – | 4,821* | 0 |
| **At production grinder discharge** | 15 | 1,190 | 9.2 | 45,517 | 0 | 664 | 9.1 | 25,004 | 0 |
| **Production grinding** | 26 | 411.4 | 8.5 | 13,867 | 0 | 239 | 8.0 | 7,299 | 0 |
| **Dumping whole beans into grinder** | 2 | – | – | 372* | 0 | – | – | 114* | 0 |
| **Above bag or bin of ground coffee** | 14 | 13.0 | 407 | 238,480 | 43 | 128 | 20.2 | 17,502 | 15 |
| **At production grinder** | 5 | 93.6 | 1.8 | 255 | 0 | 39.8 | 1.6 | 87.4 | 0 |
| **Approximate breathing zone height above ground coffee being dumped into hopper** | 3 | – | – | 173* | 0 | – | – | 47.3* | 0 |
| **Approximate breathing zone height above bag or bin of ground coffee** | 3 | – | – | 24.0* | 0 | – | – | 8.3* | 0 |
| **QC grinding related sources** | | | | | | | | | |
| **QC grinding** | 8 | 66.1 | 5.0 | 944 | 0 | 55.8 | 4.8 | 732 | 0 |
| **At QC grinder** | 1 | 5.2 | – | – | 0 | 4.8 | – | – | 0 |
| **Cafe grinding related sources** | | | | | | | | | |
| **At café grinder discharge** | 2 | – | – | 1,496* | 0 | – | – | 1,429* | 0 |
| **Café grinding** | 4 | – | – | 215* | 0 | – | – | 229* | 0 |
| **At café grinder** | 1 | 8.4 | – | – | 0 | 9.0 | – | – | 0 |
| **Moving roasted beans related sources** | | | | | | | | | |
| **Scooping beans from storage container** | 1 | 215† | – | – | 0 | 108† | – | – | 0 |
| **Transfer of roasted beans** | 1 | 184† | – | – | 0 | 98.1† | – | – | 0 |
| **Loading roasted beans** | 1 | 14.7† | – | – | 0 | 6.8† | – | – | 0 |
| **Dumping roasted beans into hopper/storage bin/destoner** | 11 | 11.0 | 5.1 | 160 | 0 | 6.2 | 5.0 | 87.5 | 0 |
| **Cleaning machines related sources** | | | | | | | | | |
| **Cleaning chaff out of roaster exhaust line** | 3 | – | – | 32.0* | 0 | – | – | 20.8* | 0 |
| **Packaging related sources** | | | | | | | | | |
| **At packaging machine (single serve pods with ground coffee)** | 5 | 176 | 1.9 | 508 | 0 | 131 | 3.1 | 826 | 0 |
| **Packaging ground coffee** | 7 | 29.6 | 3.8 | 260 | 14 | 19.3 | 3.9 | 180 | 14 |
| **Above 5 lb bag of packaged coffee** | 3 | – | – | 344* | 33 | – | – | 468* | 0 |
| **At dispenser of packaging machine** | 14 | 24.0 | 4.7 | 301 | 0 | 17.5 | 3.9 | 164 | 0 |
| **Hand packing coffee** | 7 | 76.9 | 1.6 | 159 | 0 | 35.2 | 1.6 | 74.1 | 0 |
| **At entry point for coffee into packaging machine** | 4 | – | – | 125* | 0 | – | – | 66.0* | 0 |
| **In packaging machine** | 3 | – | – | 71.8* | 0 | – | – | 51.0* | 0 |
| **Packaging coffee, unspecified** | 4 | – | – | 71.9* | 0 | – | – | 43.7* | 0 |
| **Packaging whole bean coffee** | 3 | – | – | 42.5* | 0 | – | – | 47.3* | 0 |
| **At enclosure where 5 lb bags are filled** | 1 | 27.8† | – | – | 0 | 10.4† | – | – | 0 |
| **Packing bags into box** | 2 | – | – | 29.6* | 0 | – | – | 10.7* | 0 |
| **At autosealer or heat sealer** | 3 | – | – | 16.3* | 0 | – | – | 8.8* | 0 |
| **Dispensing beans into packaging hopper** | 1 | 0.3† | – | – | 0 | <LOD | – | – | 100 |
| **Coffee storage and off-gassing sources** | | | | | | | | | |
| **Above bins or packages in off-gassing area** | 2 | – | – | 10,541* | 0 | – | – | 4,123* | 0 |
| **Opening a roasted bean storage bin** | 4 | – | – | 7,386* | 0 | – | – | 1,749* | 0 |
| **In roasted whole bean storage bin** | 32 | 613.2 | 4.4 | 6,956 | 0 | 370.9 | 4.6 | 4,624 | 0 |
| **Degassing a coffee package** | 1 | 4,322† | – | – | 0 | 1,389† | – | – | 0 |
| **In coffee storage bin, unspecified if whole bean or ground** | 10 | 64.7 | 7.7 | 1,876 | 0 | 45.1 | 6.2 | 914 | 0 |
| **Above closed storage bins** | 2 | – | – | 15.2* | 50 | – | – | 11.9* | 50 |
| **Miscellaneous production and café sources** | | | | | | | | | |
| **Making espresso (café)** | 6 | 21.7 | 6.4 | 460 | 0 | 21.8 | 6.6 | 481 | 0 |
| **Hand blending roasted beans** | 4 | – | – | 273* | 0 | – | – | 229* | 0 |
| **Above roasted whole beans** | 7 | 10.3 | 3.9 | 96.6 | 0 | 5.8 | 3.0 | 36.6 | 0 |
| **Misc QC (café and QC pour over, QC sample waste container)** | 3 | – | – | 95.9* | 0 | – | – | 105 | 0 |
| **At cold brew soaking vat (café)** | 2 | – | – | 33.1* | 0 | – | – | 42.3* | 0 |
| **Melting chocolate (café)** | 1 | 12.2† | – | – | 0 | 12.1† | – | – | 0 |
| **Misc no coffee (frothing milk in café)** | 1 | 5.0† | – | – | 0 | 3.9† | – | – | 0 |
| **Roasting coffee related sources** | | | | | | | | | |
| **Roasting** | 5 | 14.6 | 50.1 | 226* | 20 | 10.5 | 26.6 | 125* | 20 |
| **At roaster door to cooling bin** | 16 | 17.4 | 4.7 | 224 | 0 | 9.2 | 5.4 | 148 | 0 |
| **At destoner** | 3 | – | – | 63.5* | 0 | – | – | 18.0* | 0 |
| **At cooling bin** | 18 | 7.8 | 3.5 | 60.9 | 6 | 5.4 | 4.5 | 65.0 | 6 |
| **Dispensing beans from cooling bin into storage bin** | 11 | 6.4 | 2.9 | 37.1 | 0 | 2.9 | 3.2 | 19.4 | 0 |
| **Dumping green beans into hopper or roaster** | 2 | – | – | 41.0* | 0 | – | – | 28.3* | 0 |
| **Roaster checking color and/or smell of roasting beans** | 4 | – | – | 23.4* | 0 | – | – | 11.9* | 0 |
| **QC related sources** | | | | | | | | | |
| **Cupping** | 5 | 35.6 | 2.5 | 162 | 0 | 21.9 | 3.4 | 167 | 0 |

N=number of samples; GM=geometric mean; ppb=parts per billion; GSD=geometric standard deviation; P95=95^th^ percentile; %BDL=percent samples below the limit of detection; *indicates where maximum is presented when <5 measurements were above the detection limit; †indicates where raw value is presented when N=1; ─indicates not enough samples above the detection limit to obtain an estimate

**Supplementary Table 4**: Determinant distributions and univariate models for sample level determinants of diacetyl and 2,3-pentanedione considered in the multiple regression models for roasting, grinding, packaging, and overall tasks–**bold p<0.2**

| **Determinant** | **N (%)**  **Med (range)** | **β (CI)** | **N (%)**  **Med (range)** | **β (CI)** |
| --- | --- | --- | --- | --- |
| **Roasting tasks, N_w_= 34, N_t_=189** | | | | |
|  | **Diacetyl**  **% <LOD=25.4%** | | **2,3-pentanedione**  **% <LOD =23.8%** | |
| **Sampled Roaster Capacity in lbs and Enclosed** |  |  |  |  |
| Small capacity (< 200), no enclosure (intercept) | 135 (71%) | 0.68  (0.46─0.90) | 135 (71%) | 0.67  (0.48─0.86) |
| Large capacity (≥ 200), no enclosure | 28 (15%) | **2.53**  **(1.87─3.20)** | 28 (15%) | **2.03**  **(1.44─2.62)** |
| Large capacity (≥ 200), enclosed | 26 (14%) | **1.21**  **(0.54─1.88)** | 26 (14%) | **0.79**  **(0.19─1.39)** |
| **Sampled roaster near grinder or near roaster** |  |  |  |  |
| No nearby grinder or roaster (intercept) | 81 (43%) | 0.94  (0.71─1.15) | 81 (43%) | 0.87  (0.60─1.13) |
| Nearby roaster, no nearby grinder | 26 (14%) | 0.20  (-0.76─1.17) | 26 (14%) | -0.06  (-0.91─0.80) |
| Nearby grinder, no nearby roaster | 40 (21%) | -0.27  (-1.20─0.65) | 40 (21%) | -0.23  (-1.05─0.57) |
| Nearby grinder and roaster | 42 (22%) | **1.72**  **(1.12─2.32)** | 42 (22%) | **1.37**  **(0.84─1.91)** |
| **Grinding tasks, Nw= 32, Nt=74** | | | | |
|  | **Diacetyl**  **% censoring=4.1%** | | **2,3-pentanedione**  **% censoring=1.4%** | |
| **Sampled grinder, typical weight grind** |  |  |  |  |
| Small (intercept)  (≤ 5 lbs) | 47 (64%) | 3.28 (3.11─3.45) | 47 (64%) | 3.09 (2.93─3.25) |
| Large (> 5 lbs) | 27 (36%) | 0.20  (-0.33─0.72) | 27 (36%) | 0.14  (-0.36─0.63) |
| **Sampled grinder near another grinder or roaster** |  |  |  |  |
| Not near grinder or roaster (intercept) | 42 (57%) | 3.02 (2.80─3.23) | 42 (57%) | 2.94 (2.73─3.16) |
| Near grinder, not near roaster | 18 (24%) | 0.43  (-0.04─0.91) | 18 (24%) | 0.26  (-0.22─0.74) |
| Near grinder and roaster | 14 (19%) | **1.04**  **(0.53─1.55)** | 14 (19%) | **0.59**  **(0.07─1.12)** |
| **Sampled grinder enclosed and automated** |  |  |  |  |
| No (intercept) | 68 (92%) |  | 68 (92%) |  |
| Yes | 6 (8%) | **Note from one site** | 6 (8%) | **Note from one site** |
| **Sampled while grinding flavored coffee** |  |  |  |  |
| No (intercept) | 66 (89%) | 3.42  (3.28─3.57) | 66 (89%) | 3.14  (3.00─3.28) |
| Yes | 8 (11%) | **-0.62**  **(-1.15─-0.10)** | 8 (11%) | -0.10  (-0.61─0.41) |
| **Packaging tasks, N_w_=74, N_t_=203** | | | | |
|  | **Diacetyl**  **% censoring=4.9%** | | **2,3-pentanedione**  **% censoring=7.9%** | |
| **Sampled packaging volume** |  |  |  |  |
| < 1 lb (intercept) | 49 (24%) | 2.57  (2.38─2.76) | 49 (24%) | 2.08  (1.89─2.28) |
| 1─5 lbs | 5 (2%) | -0.14  (-0.66─0.38) | 5 (2%) | -0.04  (-0.56─0.48) |
| 5 lb | 26 (13%) | -0.16  (-0.48─0.16) | 26 (13%) | -0.005  (-0.33─0.34) |
| Single serve | 17 (8%) | **0.71**  **(0.23─1.18)** | 17 (8%) | **0.47**  **(-0.004─0.95)** |
| Unknown < 5 lbs | 106 (52%) | **-0.38**  **(-0.62─-0.14)** | 106 (52%) | **-0.36**  **(-0.60─-0.11)** |
| **Sampled while packaging ground coffee** |  |  |  |  |
| No (intercept) | 79 (39%) | 2.50  (2.36─2.65) | 79 (39%) | 2.07  (1.93─2.21) |
| Yes | 58 (29%) | 0.14  (-0.11─0.38) | 58 (29%) | 0.15  (-0.08─0.39) |
| Unknown | 66 (33%) | -0.33  (-0.56─-0.11) | 66 (33%) | -0.50  (-0.72─-0.28) |
| **Sampled while packaging flavored coffee** |  |  |  |  |
| No (intercept) | 177 (87%) | 2.26  (2.18─2.34) | 177 (87%) | 1.79  (1.71─1.87) |
| Yes | 10 (5%) | **0.52**  **(0.08─0.95)** | 10 (5%) | **0.78**  **(0.33─1.23)** |
| Unknown | 16 (8%) | 1.34  (0.91─1.77) | 16 (8%) | 1.13  (0.70─1.56) |
| **Overall tasks, N_w_=126, N_t_=591** | | | | |
|  | **Diacetyl**  **% censoring=14%** | | **2,3-pentanedione**  **% censoring=12.4%** | |
| **Sampled task** |  |  |  |  |
| Roasting (intercept) | 189 (32%) | 1.61  (1.45─1.79) | 189 (32%) | 1.40  (1.24─1.56) |
| Quality control (QC) | 44 (7%) | **0.39**  **(0.02─0.75)** | 44 (7%) | **0.46**  **(0.15─0.78)** |
| Packaging coffee | 203 (34%) | **0.80**  **(0.56─1.03)** | 203 (34%) | **0.52**  **(0.31─0.74)** |
| Moving roasted beans or ground coffee | 13 (2%) | **1.58**  **(1.18─1.98)** | 13 (2%) | **1.30**  **(0.92─1.68)** |
| Miscellaneous production | 17 (3%) | 0.10  (-0.37─0.56) | 17 (3%) | -0.13  (-0.56─0.30) |
| Grinding coffee beans | 74 (13%) | **1.79**  **(1.53─2.05)** | 74 (13%) | **1.64**  **(1.39─1.88)** |
| Flavoring coffee | 15 (3%) | **1.75**  **(1.34─2.15)** | 15 (3%) | **2.26**  **(1.88─2.64)** |
| Cleaning machines | 36 (6%) | 0.18  (-0.06─0.43) | 36 (6%) | -0.06  (-0.30─0.18) |

**CI indicates 80% credible interval; β is ppb for every unit change in predictor;**

**N_w_ indicates the number of workers sampled;**

**N_t_ indicates the total number of samples**

**Supplementary Table 5.** Determinant distributions and univariate models for process level determinants of diacetyl considered in the multiple regression models for roasting, grinding, packaging, and overall tasks–**bold p<0.2**

| **Determinant** | **Roasting**  **N_w_= 34**  **N_t_=189**  **% censoring=25.4%** | | **Grinding**  **N_w_= 32**  **N_t_=74**  **% censoring=4.1%** | | **Packaging**  **N_w_=74**  **N_t_=203**  **% censoring=4.9%** | | **Overall**  **N_w_=126**  **N_t_=591**  **% censoring=14%** | |
| --- | --- | --- | --- | --- | --- | --- | --- | --- |
|  | **N (%)**  **Med (range)** | **β (CI)** | **N (%)**  **Med (range)** | **β (CI)** | **N (%)**  **Med (range)** | **β (CI)** | **N (%)**  **Med (range)** | **β (CI)** |
| **Coffee Storage Determinants** | | | | | | | | |
| **Sum of all open storage sources** |  |  |  |  |  |  |  |  |
| None (intercept) | 106 (56%) | 0.15  (-0.18─0.47) | 37 (50%) | 3.14  (2.93─3.35) | 52 (26%) | 1.63  (1.42─1.84) | 223 (38%) | 1.51  (1.32─1.70) |
| Small (1-2) | 23 (12.2%) | **1.57**  **(0.90─2.26)** | 13 (18%) | 0.23  (-0.40─0.85) | 39 (19%) | **1.00**  **(0.60─1.40)** | 92 (16%) | **0.83**  **(0.44─1.22)** |
| Large (> 2) | 60 (32%) | **2.30**  **(1.72─2.89)** | 24 (32%) | **0.62**  **(0.11─1.12)** | 112 (55%) | **1.15**  **(0.86─1.43)** | 276 (47%) | **1.26**  **(0.98─1.54)** |
| **Open storage in task area versus elsewhere** | **In roasting area** | **In roasting area** |  |  | **In packaging area** | **In packaging area** |  |  |
| No open storage (intercept) | 106 (56%) | 0.15  (-0.18─0.47) | ─ | ─ | 52 (26%) | 1.63  (1.43─1.84) | ─ | ─ |
| Open storage in task area | 49 (26%) | **2.56**  **(1.94─3.17)** | ─ | ─ | 64 (32%) | **1.27**  **(0.96─1.58)** | ─ | ─ |
| Open storage elsewhere | 34 (18%) | **1.50**  **(0.88─2.13)** | ─ | ─ | 87 (43%) | **0.95**  **(0.64─1.26)** | ─ | ─ |
| **Ground coffee storage container in grinding area** |  |  |  |  |  |  |  |  |
| Open container (intercept) | ─ | ─ | 12 (16%) | 3.44 (3.02─3.86) |  | ─ | ─ | ─ |
| Containers with lids*, # | ─ | ─ | 4 (5%) | **-1.15**  **(-2.00─-0.30)** |  | ─ | ─ | ─ |
| Packaging | ─ | ─ | 42 (57%) | -0.07  (-0.59─0.46) |  | ─ | ─ | ─ |
| Permeable supersack | ─ | ─ | 10 (14%) | -0.10  (-1.21─1.01) |  | ─ | ─ | ─ |
| Silo# | ─ | ─ | 6 (8%) | 0.72  (-0.39─1.83) |  | ─ | ─ | ─ |
| **General Sources Determinants** | | | | | | | | |
| **Total number of sources** |  |  |  |  |  |  |  |  |
| Small (intercept) (≤ 7) | 116 (61%) | 0.43  (0.12─0.73) | 44 (59%) | 3.18  (3.00─3.37) | 68 (33%) | 1.86  (1.67─2.06) | 264 (45%) | 1.74  (1.57─1.91) |
| Large (> 7) | 73 (39%) | **1.80**  **(1.23─2.38)** | 30 (41%) | **0.47**  **(0.01─0.94)** | 135 (67%) | **0.86**  **(0.58─1.14)** | 327 (55%) | **0.92**  **(0.65─1.19)** |
| **Number of other sources near roasters** |  |  |  |  |  |  |  |  |
| None | 51 (27%) | 0.86  (0.36─1.35) | ─ | ─ | ─ | ─ | ─ | ─ |
| 1 | 64 (34%) | -0.28  (-1.05─0.49) | ─ | ─ | ─ | ─ | ─ | ─ |
| > 1 | 74 (39%) | **1.57**  **(0.81─2.34)** | ─ | ─ | ─ | ─ | ─ | ─ |
| **Number of other sources near grinders** |  |  |  |  |  |  |  |  |
| Intercept | ─ | ─ | 2  (0─5) | 2.94  (2.69─3.19) | ─ | ─ | ─ | ─ |
| Slope | ─ | ─ |  | **0.20**  **(0.09─0.31)** | ─ | ─ | ─ | ─ |
| **Process Determinants** | | | | | | | | |
| **Amount of roasted coffee produced** | | | | | | | | |
| **Average roasted coffee production, lbs per day** |  |  |  |  |  |  |  |  |
| Small (intercept)  (< 1,000) | 54 (29%) | 0.58  (0.15─0.99) | 21 (28%) | 2.81  (2.53─3.10) | 37 (18%) | 1.57  (1.29─1.84) | 139 (24%) | 1.37  (1.11─1.63) |
| Medium  (≥ 1,000 and < 10,000) | 75 (40%) | 0.10  (-0.65─0.87) | 29 (39%) | **0.72**  **(0.28─1.16)** | 54 (26%) | **0.71**  **(0.31─1.11)** | 176 (30%) | **0.70**  **(0.33─1.07)** |
| Large  **(≥** 10,000) | 60 (32%) | **1.86**  **(1.17─2.54)** | 24 (32%) | **0.95**  **(0.45─1.45)** | 112 (55%) | **1.21**  **(0.87─1.55)** | 276 (47%) | **1.40**  **(1.07─1.73)** |
| **Total number of roasters** |  |  |  |  |  |  |  |  |
| 1 (intercept) | 70 (37%) | 0.68  (0.24─1.11) | 30 (41%) | 3.06  (2.82─3.30) | 51 (25%) | 2.19  (1.91─2.47) | 179 (30%) | 1.82  (1.59─2.06) |
| 2─3 | 119 (63%) | **0.99**  **(0.29─1.69)** | 44 (59%) | **0.53**  **(0.12─0.94)** | 152 (75%) | 0.31  (-0.04─0.66) | 412 (70%) | **0.34**  **(0.06─0.63)** |
| **Total roaster capacity in lbs** |  |  |  |  |  |  |  |  |
| Intercept | 200  (25─1000) | 0.40  (0.11─0.68) | 141  (26─1000) | 3.07  (2.87─3.27) | 670  (25─1000) | 1.85  (1.66─2.03) | 200  (25─1000) | 1.59  (1.42─1.76) |
| Slope |  | **0.0025**  **(0.002─0.003)** |  | **0.001**  **(0.0004─0.002)** |  | **0.001**  **(0.0008─0.0015)** |  | **0.001**  **(0.001─0.002)** |
| **Total number of package lines** |  |  |  |  |  |  |  |  |
| Intercept | 5  (0─9) | 0.52  (0.17─0.85) | 2  (0─9) | 3.08  (2.87─3.30) | 5  (0─9) | 1.98  (1.78─2.18) | 2  (0─9) | 1.74  (1.56─1.92) |
| Slope |  | **0.23**  **(0.14─0.32)** |  | **0.09**  **(0.02─0.16)** |  | **0.10**  **(0.06─0.14)** |  | **0.13**  **(0.09─0.17)** |
| **Type of roasted coffee produced (roast depth)** | | | | | | | | |
| **Average roast length in minutes**† |  |  |  |  |  |  |  |  |
| < 15 minutes | 63 (33%) | 0.60  (0.11─1.09) | 31 (42%) | 3.21  (2.87─3.54) | 99 (49%) | 2.23  (2.03─2.42) | 259 (44%) | 2.02  (1.83─2.21) |
| ≥ 15 minutes | 118 (62%) | **0.98**  **(0.22─1.75)** | 38 (51%) | 0.20  (-0.29─0.68) | 97 (48%) | **0.34**  **(0.04─0.64)** | 309 (52%) | **0.46**  **(0.16─0.75)** |
| **Amount of grinding performed and grinding process determinants** | | | | | | | | |
| **Average percent ground coffee of total production** |  |  |  |  |  |  |  |  |
| Intercept | 27  (3─65) | -0.09  (-0.51─0.33) | 27  (3─65) | 2.76  (2.51─3.02) | 27  (3─65) | 1.46  (1.26─1.66) | 27  (3─65) | 1.25  (1.06─1.45) |
| Slope |  | **0.05**  **(0.03─0.06)** |  | **0.02**  **(0.01─0.03)** |  | **0.03**  **(0.02─0.04)** |  | **0.033**  **(0.03─0.04** |
| **Average grind length in mins** |  |  |  |  |  |  |  |  |
| Intercept | 4.5  (0.3─60) | 0.67  (0.41─0.91) | 3  (0.3─60) | 3.17  (3.00─3.34) | 15  (0.3─60) | 2.07  (1.92─2.22) | 5.5  (0.3─60) | 1.87  (1.73─2.01) |
| Slope |  | **0.03**  **(0.02─0.05)** |  | **0.01**  **(0.003─0.02)** |  | **0.01**  **(0.009─0.02)** |  | **0.02**  **(0.01─0.02)** |
| **Total number of grinders** |  |  |  |  |  |  |  |  |
| Intercept | 3  (0─9) | 0.41  (-0.16─0.96) | 3  (1─9) | 2.94  (2.65─3.23) | 3  (0─9) | 1.88  (1.63─2.13) | 3  (0─9) | 1.76  (1.52─2.00) |
| Slope |  | **0.25**  **(0.10─0.42)** |  | **0.12**  **(0.04─0.20)** |  | **0.14**  **(0.08─0.20)** |  | **0.14**  **(0.08─0.20)** |
| **Total grinding capacity in lbs** |  |  |  |  |  |  |  |  |
| Intercept | 50  (4─2001) | 0.77  (0.53─1.00) | 50  (4─2001) | 3.19  (3.03─3.36) | 405  (4─2001) | 2.19  (2.05─2.34) | 76  (4─2001) | 1.99  (1.86─2.12) |
| Slope |  | **0.001**  **(0.0007─0.002)** |  | **0.0005**  **(0.00008─0.0009)** |  | **0.0003**  **(0.0002─0.0005)** |  | **0.0005**  **(0.0003─0.0007)** |
| **Grind flavored coffee** |  |  |  |  |  |  |  |  |
| No | 163 (86%) | 1.23  (1.04─1.41) | 61 (82%) | 3.36  (3.19─3.53) | 178 (88%) | 2.37  (2.28─2.45) | 505 (85%) | 2.29  (2.21─2.36) |
| Yes | 26 (14%) | 0.25  (-0.75─1.25) | 13 (18%) | -0.15  (-0.64─0.35) | 25 (12%) | **0.53**  **(0.08─0.98)** | 86 (15%) | 0.10  (-0.33─0.53) |
| **Flavoring process determinants** | | | | | | | | |
| **Flavor (and package) ground coffee, accessory fans in flavoring room, and isolated flavoring room** |  |  |  |  |  |  |  |  |
| Flavor (and package) whole bean coffee, do not flavor ground coffee/no accessory fans/no isolated flavoring room | 21 (11%) | 1.40  (0.52─2.28) | 6 (8%) | 2.48  (1.94─3.04) | 11 (5%) | 1.88  (1.34─2.43) | 49 (8%) | 1.42  (0.92─1.91) |
| Flavor (and package) ground and whole bean coffee/accessory fans/isolated flavoring room | 28 (15%) | **1.36**  **(0.12─2.58)** | 13 (18%) | **1.50**  **(0.76─2.24)** | 38 (19%) | **1.46**  **(0.84─2.09)** | 116 (20%) | **1.88**  **(1.31─2.46)** |
| Do not flavor coffee | 140 (74%) | -0.46  (-1.47─0.55) | 55 (74%) | **0.83**  **(0.21─1.44)** | 154 (76%) | **0.33**  **(-0.25─0.91)** | 426 (72%) | **0.68**  **(0.15─1.21)** |
| **Flavoring during survey** |  |  |  |  |  |  |  |  |
| No flavoring (intercept) | 146 (77%) | 0.94  (0.71─1.15) | 55 (74%) | 3.31  (3.13─3.49) | 157 (77%) | 2.17  (2.07─2.26) | 435 (74%) | 2.05  (1.95─2.14) |
| Flavoring | 43 (22.8%) | **1.40**  **(0.63─2.17)** | 19 (26%) | 0.04  (-0.43─0.51) | 46 (23%) | **1.06**  **(0.76─1.36)** | 156 (26%) | **1.02**  **(0.71─1.32)** |
| **Engineering Controls Determinants** | | | | | | | | |
| **Percent automated sources** |  |  |  |  |  |  |  |  |
| None (intercept) | 114 (60%) | 0.45  (0.17─0.72) | 41 (55%) | 3.08  (2.89─3.26) | 59 (29%) | 1.74  (1.55─1.93) | 247 (42%) | 1.60  (1.43─1.78) |
| Low (≤ 6) | 43 (23%) | **1.47**  **(0.84─2.11)** | 18 (24%) | **0.58**  **(0.07─1.10)** | 91 (44%) | **0.81**  **(0.52─1.10)** | 206 (35%) | **0.88**  **(0.59─1.17)** |
| High (> 6) | 32 (17%) | **2.49**  **(1.73─3.26)** | 15 (20%) | **0.99**  **(0.35─1.63)** | 53 (26%) | **1.33**  **(1.00─1.67)** | 138 (23%) | **1.45**  **(1.13─1.79)** |
| **Any isolated sources/processes** |  |  |  |  |  |  |  |  |
| No (intercept) | 95 (50%) | 0.80  (0.37─1.23) | 31 (42%) | 3.37  (3.12─3.61) | 47 (23%) | 1.93  (1.68─2.18) | 189 (32%) | 1.89  (1.67─2.12) |
| Yes | 94 (50%) | **0.79**  **(0.09─1.49)** | 43 (58%) | -0.09  (-0.51─0.34) | 156 (77%) | **0.66**  **(0.34─0.98)** | 402 (68%) | **0.56**  **(0.26─0.87)** |
| **Any enclosed sources** |  |  |  |  |  |  |  |  |
| No (intercept) | 140 (74%) | 0.72  (0.51─0.93) | 57 (77%) | 3.29  (3.15─3.43) | 108 (53%) | 2.13  (1.98─2.28) | 361 (61%) | 1.91  (1.77─2.04) |
| Yes | 94 (49%) | **1.93**  **(1.32─2.54)** | 17 (23%) | 0.31  (-0.37─0.98) | 95 (47%) | **0.61**  **(0.33─0.89)** | 230 (39%) | **0.87**  **(0.61─1.13)** |
| **GEV (with mechanical supply)**† |  |  |  |  |  |  |  |  |
| No (intercept) | 14 (7%) | 2.34  (1.40─3.28) | 10 (14%) | 3.72  (3.21─4.23) | 21 (10%) | 3.14  (2.68─3.60) | 61 (10%) | 2.98  (2.54─3.41) |
| Yes | 158 (84%) | **-1.12**  **(-2.18─-0.05)** | 61 (82%) | -0.46  (-1.05─0.12) | 174 (86%) | **-0.73**  **(-1.23─-0.23)** | 502 (85%) | **-0.68**  **(-1.15─-0.20)** |
| **Natural ventilation**† |  |  |  |  |  |  |  |  |
| No (intercept) | 98 (52%) | 1.72  (1.40─2.04) | 27 (36%) | 3.24  (2.93─3.55) | 91 (45%) | 2.58  (2.43─2.73) | 264 (45%) | 2.44  (2.29─2.58) |
| Yes | 44 (23%) | **-1.10**  **(-2.00─-0.21)** | 22 (30%) | -0.16  (-0.66─0.35) | 37 (18%) | **-1.03**  **(-1.03─-0.22)** | 132 (22%) | **-0.63**  **(-1.01─-0.24)** |
| **Accessory fans at the roasters** |  |  |  |  |  |  |  |  |
| No (intercept) | 107 (57%) | 0.96  (0.59─1.33) | 40 (54%) | 3.38  (3.16─3.61) | 77 (38%) | 2.41  (2.22─2.59) | 282 (48%) | 2.33  (2.16─2.50) |
| Yes | 82 (43%) | 0.63  (-0.09─1.37) | 34 (46%) | -0.14  (-0.57─0.30) | 126 (62%) | 0.03  (-0.27─0.33) | 309 (52%) | -0.05  (-0.33─0.23) |
| **Isolated roasting area** |  |  |  |  |  |  |  |  |
| No (intercept) | 178 (94%) | 1.26  (1.11─1.40) | 67 (91%) | 3.24  (3.10─3.39) | 186 (92%) | 2.38  (2.30─2.45) | 545 (92%) | 2.26  (2.19─2.33) |
| Yes# | 11 (6%) | 0.09  (-1.38─1.57) | 7 (9%) | **0.67**  **(0.05─1.31)** | 17 (8%) | **0.69**  **(0.13─1.24)** | 46 (8%) | 0.48  (-0.06─1.01) |
| **Flavoring Ventilation (LEV)** |  |  |  |  |  |  |  |  |
| Flavoring, no LEV | 15 (8%) | 1.68  (0.78─2.57) | 9 (12%) | 3.71  (3.19-4.23) | 21 (10%) | 3.17  (2.75─3.59) | 61 (10%) | 2.70  (2.30─3.12) |
| Flavoring, LEV | 28 (15%) | 1.25  (-0.05─2.55) | 10 (14%) | -0.80  (-1.62─0.008) | 25 (12%) | 0.09  (-0.47─0.64) | 95 (16%) | 0.53  (-0.005─1.07) |
| No flavoring during survey | 146 (77%) | -0.74  (-1.75─0.28) | 55 (74%) | -0.40  (-1.00─0.21) | 157 (77%) | **-1.01**  **(-1.47─-0.54)** | 435 (74%) | **-0.65**  **(-1.10─-0.20)** |

**N_w_ indicates the number of workers sampled; N_t_ indicates the total number of samples; β is for every unit change in predictor; CI indicates 80% credible interval;**

***note small cell size (n<5);**

†**indicates variables with some information not recorded (missing values, accounted for in modeling).**

**Note: GM=e^(intercept + β)^**

**Supplementary Table 6.** Determinant distributions and univariate models for process level determinants of 2,3-pentanedione considered in the multiple regression models for roasting, grinding, packaging, and overall tasks–**bold p<0.2**

| **Determinant** | **Roasting**  **N_w_= 34**  **N_t_=189**  **% censoring=23.8%** | | **Grinding**  **N_w_= 32**  **N_t_=74**  **% censoring=1.4%** | | **Packaging**  **N_w_=74**  **N_t_=203**  **% censoring=7.9%** | | **Overall**  **N_w_=126**  **N_t_=591**  **% censoring=12.4%** | |
| --- | --- | --- | --- | --- | --- | --- | --- | --- |
|  | **N (%)**  **Med (range)** | **β (CI)** | **N (%)**  **Med (range)** | **β (CI)** | **N (%)**  **Med (range)** | **β (CI)** | **N (%)**  **Med (range)** | **β (CI)** |
| **Coffee Storage Determinants** | | | | | | | | |
| **Sum of all open storage sources** |  |  |  |  |  |  |  |  |
| None (intercept) | 106 (56%) | 0.19  (-0.09─0.47) | 37 (50%) | 2.93  (2.73─3.12) | 52 (26%) | 1.34  (1.12─1.56) | 223 (38%) | 1.40  (1.21─1.59) |
| Small (1-2) | 23 (12.2%) | **1.61**  **(0.97─2.25)** | 13 (18%) | 0.20  (-0.38─0.78) | 39 (19%) | **1.07**  **(0.63─1.49)** | 92 (16%) | **0.71**  **(0.32─1.09)** |
| Large (> 2) | 60 (32%) | **1.75**  **(1.24─2.26)** | 24 (32%) | **0.71**  **(0.24─1.19)** | 112 (55%) | **0.77**  **(0.47─1.08)** | 276 (47%) | **0.80**  **(0.52─1.07)** |
| **Open storage in task area versus elsewhere** | **In roasting area** | **In roasting area** |  |  | **In packaging area** | **In packaging area** |  |  |
| No open storage (intercept) | 106 (56%) | 0.21  (-0.06─0.47) | ─ | ─ | 52 (26%) | 1.35  (1.12─1.57) | ─ | ─ |
| Open storage in task area | 49 (26%) | **1.95**  **(1.44─2.46)** | ─ | ─ | 64 (32%) | **0.86**  **(0.53─1.20)** | ─ | ─ |
| Open storage elsewhere | 34 (18%) | **1.37**  **(0.81─1.94)** | ─ | ─ | 87 (43%) | **0.80**  **(0.46─1.14)** | ─ | ─ |
| **Ground coffee storage container in grinding area** |  |  |  |  |  |  |  |  |
| Open container (intercept) | ─ | ─ | 12 (16%) | 3.56  (3.17─3.95) | ─ | ─ | ─ | ─ |
| Containers with lids* | ─ | ─ | 4 (5%) | **-1.48**  **(-2.25─-0.70** | ─ | ─ | ─ | ─ |
| Packaging | ─ | ─ | 42 (57%) | -0.43  (-0.91─0.06) | ─ | ─ | ─ | ─ |
| Permeable supersack | ─ | ─ | 10 (14%) | **-0.97**  **(-1.93─-0.02)** | ─ | ─ | ─ | ─ |
| Silo | ─ | ─ | 6 (8%) | -0.07  (-1.07─0.92) | ─ | ─ | ─ | ─ |
| **General Sources Determinants** | | | | | | | | |
| **Total number of sources** |  |  |  |  |  |  |  |  |
| Small (intercept) (≤ 7) | 116 (61%) | 0.47  (0.21─0.73) | 44 (59%) | 2.93  (2.76─3.11) | 68 (33%) | 1.58  (1.37─1.79) | 264 (45%) | 1.60  (1.43─1.76) |
| Large (> 7) | 73 (39%) | **1.40**  **(0.89─1.91)** | 30 (41%) | **0.64**  **(0.20─1.08)** | 135 (67%) | **0.55**  **(0.25─0.85)** | 327 (55%) | **0.54**  **(0.28─0.80)** |
| **Number of other sources near roasters** |  |  |  |  |  |  |  |  |
| None | 51 (27%) | 0.70  (0.26─1.13) | ─ | ─ | ─ | ─ | ─ | ─ |
| 1 | 64 (34%) | -0.03  (-0.70─0.63) | ─ | ─ | ─ | ─ | ─ | ─ |
| > 1 | 74 (39%) | **1.33**  **(0.66─2.01)** | ─ | ─ | ─ | ─ | ─ | ─ |
| **Number of other sources near grinders** |  |  |  |  |  |  |  |  |
| Intercept | ─ | ─ | 2  (0─5) | 2.98  (2.73─3.23) | ─ | ─ | ─ | ─ |
| Slope | ─ | ─ |  | 0.07  (-0.04─0.19) | ─ | ─ | ─ | ─ |
| **Process Determinants** | | | | | | | | |
| **Amount of roasted coffee produced** | | | | | | | | |
| **Average roasted coffee production, lbs per day** |  |  |  |  |  |  |  |  |
| Small (intercept)  (< 1,000) | 54 (29%) | 0.67  (0.28─1.05) | 21 (28%) | 2.59  (2.32─2.86) | 37 (18%) | 1.30  (1.00─1.60) | 139 (24%) | 1.29  (1.04─1.54) |
| Medium  (≥ 1,000 and < 10,000) | 75 (40%) | -0.01  (-0.69─0.67) | 29 (39%) | **0.71**  **(0.29─1.12)** | 54 (26%) | **0.71**  **(0.28─1.13)** | 176 (30%) | **0.57**  **(0.21─0.94)** |
| Large  **(≥** 10,000) | 60 (32%) | **1.30**  **(0.68─1.94)** | 24 (32%) | **1.02**  **(0.55─1.49)** | 112 (55%) | **0.82**  **(0.44─1.19)** | 276 (47%) | **0.91**  **(0.59─1.23)** |
| **Total number of roasters** |  |  |  |  |  |  |  |  |
| 1 (intercept) | 70 (37%) | 0.61  (0.24─0.98) | 30 (41%) | 2.90  (2.66─3.13) | 51 (25%) | 1.82  (1.54─2.11) | 179 (30%) | 1.67  (1.45─1.89) |
| 2─3 | 119 (63%) | **0.85**  **(0.25─1.44)** | 44 (59%) | **0.44**  **(0.05─0.84)** | 152 (75%) | 0.15  (-0.20─0.50) | 412 (70%) | **0.34**  **(0.06─0.63)** |
| **Total roaster capacity in lbs** |  |  |  |  |  |  |  |  |
| Intercept | 200  (25─1000) | 0.46  (0.20─0.70) | 141  (26─1000) | 2.87  (2.67─3.06) | 670  (25─1000) | 1.65  (1.45─1.85) | 200  (25─1000) | 1.51  (1.35─1.68) |
| Slope |  | **0.002**  **(0.001─0.003)** |  | **0.001**  **(0.0004─0.0016)** |  | **0.001**  **(0.0002─0.001)** |  | **0.0008**  **(0.0005─0.001)** |
| **Total number of package lines** |  |  |  |  |  |  |  |  |
| Intercept | 5  (0─9) | 0.55  (0.26─0.84) | 2  (0─9) | 2.81  (2.61─3.01) | 5  (0─9) | 1.73  (1.53─1.94) | 2  (0─9) | 1.61  (1.43─1.78) |
| Slope |  | **0.17**  **(0.09─0.25)** |  | **0.12**  **(0.06─0.18)** |  | **0.04**  **(0.002─0.09)** |  | **0.07**  **(0.03─0.11)** |
| **Type of roasted coffee produced (roast depth)** | | | | | | | | |
| **Average roast length in minutes**† |  |  |  |  |  |  |  |  |
| < 15 minutes | 63 (33%) | 0.79  (0.37─1.20) | 31 (42%) | 2.94  (2.63─3.25) | 99 (49%) | 1.94  (1.75─2.14) | 259 (44%) | 1.76  (1.59─1.94) |
| ≥ 15 minutes | 118 (62%) | 0.49  (-0.16─1.15) | 38 (51%) | 0.35  (-0.10─0.79) | 97 (48%) | 0.014  (-0.29─0.32) | 309 (52%) | 0.26  (-0.008─0.54) |
| **Amount of grinding performed and grinding process determinants** | | | | | | | | |
| **Average percent ground coffee** |  |  |  |  |  |  |  |  |
| Intercept | 27  (3─65) | 0.13  (-0.24─0.50) | 27  (3─65) | 2.52  (2.29─2.77) | 27  (3─65) | 1.17  (0.96─1.39) | 27  (3─65) | 1.14  (0.95─1.34) |
| Slope |  | **0.03**  **(0.02─0.05)** |  | **0.02**  **(0.01─0.03)** |  | **0.024**  **(0.02─0.03)** |  | **0.025**  **(0.02─0.03)** |
| **Average grind length in mins** |  |  |  |  |  |  |  |  |
| Intercept | 4.5  (0.3─60) | 0.66  (0.44─0.88) | 3  (0.3─60) | 2.90  (2.74─3.06) | 15  (0.3─60) | 1.76  (1.61─1.92) | 5.5  (0.3─60) | 1.67  (1.54─1.80) |
| Slope |  | **0.03**  **(0.02─0.04)** |  | **0.02**  **(0.009─0.03)** |  | **0.007**  **(0.001─0.013)** |  | **0.01**  **(0.006─0.016)** |
| **Total number of grinders** |  |  |  |  |  |  |  |  |
| Intercept | 3  (0─9) | 0.55  (0.07─1.01) | 3  (1─9) | 2.60  (2.32─2.87) | 3  (0─9) | 1.43  (1.17─1.68) | 3  (0─9) | 1.50  (1.27─1.73) |
| Slope |  | **0.17**  **(0.04─0.31)** |  | **0.16**  **(0.09─0.24)** |  | **0.13**  **(0.07─0.19)** |  | **0.11**  **(0.05─0.16)** |
| **Total grinding capacity in lbs** |  |  |  |  |  |  |  |  |
| Intercept | 50  (4─2001) | 0.73  (0.53─0.93) | 50  (4─2001) | 2.95  (2.79─3.10) | 405  (4─2001) | 1.90  (1.75─2.04) | 76  (4─2001) | 1.79  (1.66─1.91) |
| Slope |  | **0.0009**  **(0.0005─0.001)** |  | **0.0006**  **(0.0003─0.001)** |  | 0.0001  (-0.0001─0.0003) |  | **0.0002**  **(0.00003─0.0004)** |
| **Grind flavored coffee** |  |  |  |  |  |  |  |  |
| No | 163 (86%) | 1.10  (0.94─1.25) | 61 (82%) | 3.04  (2.88─3.20) | 178 (88%) | 1.85  (1.77─1.93) | 505 (85%) | 1.86  (1.79─1.93) |
| Yes | 26 (14%) | 0.18  (-0.66─1.02) | 13 (18%) | 0.34  (-0.12─0.80) | 25 (12%) | **0.80**  **(0.35─1.24)** | 86 (15%) | **0.52**  **(0.13─0.90)** |
| **Flavoring process determinants** | | | | | | | | |
| **Flavor (and package) ground coffee, accessory fans in flavoring room, and isolated flavoring room** |  |  |  |  |  |  |  |  |
| Flavor (and package) whole bean coffee, do not flavor ground coffee/no accessory fans/no isolated flavoring room | 21 (11%) | 1.25  (0.50─2.02) | 6 (8%) | 2.42  (1.92─2.92) | 11 (5%) | 1.22  (0.65─1.77) | 49 (8%) | 1.31  (0.85─1.77) |
| Flavor (and package) ground and whole bean coffee/accessory fans/isolated flavoring room | 28 (15%) | 1.01  (-0.07─2.07) | 13 (18%) | **1.66**  **(1.00─2.32)** | 38 (19%) | **1.68**  **(1.06─2.32)** | 116 (20%) | **1.57**  **(1.03─2.11)** |
| Do not flavor coffee | 140 (74%) | **-0.39**  **(-1.25─0.48)** | 55 (74%) | **0.60**  **(0.05─1.15)** | 154 (76%) | 0.51  (-0.07─1.10) | 426 (72%) | 0.40  (-0.09─0.89) |
| **Flavoring during survey** |  |  |  |  |  |  |  |  |
| No flavoring (intercept) | 146 (77%) | 0.86  (0.67─1.04) | 55 (74%) | 3.02  (2.85─3.19) | 157 (77%) | 1.68  (1.59─1.78) | 435 (74%) | 1.66  (1.57─1.75) |
| Flavoring | 43 (22.8%) | **1.13**  **(0.48─1.78)** | 19 (26%) | 0.38  (-0.05─0.81) | 46 (23%) | **1.07**  **(0.78─1.36)** | 156 (26%) | **1.06**  **(0.79─1.34)** |
| **Engineering Controls Determinants** | | | | | | | | |
| **Percent automated sources** |  |  |  |  |  |  |  |  |
| None (intercept) | 114 (60%) | 0.48  (0.24─0.72) | 41 (55%) | 2.87  (2.69─3.05) | 59 (29%) | 1.47  (1.26─1.67) | 247 (42%) | 1.48  (1.31─1.65) |
| Low (≤ 6) | 43 (23%) | **1.04**  **(0.48─1.60)** | 18 (24%) | **0.78**  **(0.30─1.27)** | 91 (45%) | **0.43**  **(0.12─0.73)** | 206 (35%) | **0.45**  **(0.18─0.73)** |
| High (> 6) | 32 (17%) | **2.08**  **(1.41─2.75)** | 15 (20%) | **0.74**  **(0.12─1.35)** | 53 (26%) | **1.13**  **(0.78─1.48)** | 138 (23%) | **1.12**  **(0.80─1.44)** |
| **Any isolated sources/processes** |  |  |  |  |  |  |  |  |
| No (intercept) | 95 (50%) | 0.59  (0.24─0.95) | 31 (42%) | 3.07  (2.83─3.30) | 47 (23%) | 1.46  (1.22─1.71) | 189 (32%) | 1.57  (1.36─1.79) |
| Yes | 94 (50%) | **0.91**  **(0.32─1.49)** | 43 (58%) | 0.10  (-0.31─0.50) | 156 (77%) | **0.63**  **(0.31─0.96)** | 402 (68%) | **0.49**  **(0.20─0.77)** |
| **Any enclosed sources** |  |  |  |  |  |  |  |  |
| No (intercept) | 140 (74%) | 0.70  (0.51─0.88) | 57 (77%) | 3.15  (3.01─3.28) | 108 (53%) | 1.89  (1.74─2.05) | 361 (61%) | 1.77  (1.64─1.90) |
| Yes | 94 (49%) | **1.48**  **(0.94─2.03)** | 43 (58%) | -0.21  (-0.85─0.42) | 95 (47%) | 0.09  (-0.21─0.38) | 230 (39%) | **0.34**  **(0.08─0.61)** |
| **GEV (with mechanical supply)**† |  |  |  |  |  |  |  |  |
| No (intercept) | 14 (7%) | 2.26  (1.51─3.02) | 10 (14%) | 3.50  (3.02─3.98) | 21 (10%) | 2.80  (2.35─3.25) | 61 (10%) | 2.76  (2.36─3.16) |
| Yes | 158 (84%) | **-1.18**  **(-2.03─-0.33)** | 61 (82%) | -0.42  (-0.97─0.13) | 174 (86%) | **-0.89**  **(-1.38─-0.41)** | 502 (85%) | **-0.85**  **(-1.28─-0.41)** |
| **Natural ventilation**† |  |  |  |  |  |  |  |  |
| No (intercept) | 98 (52%) | 1.45  (1.17─1.73) | 27 (36%) | 2.90  (2.62─3.18) | 91 (45%) | 1.93  (1.78─2.09) | 264 (45%) | 1.91  (1.78─2.05) |
| Yes | 44 (23%) | -0.53  (-1.29─0.24) | 22 (30%) | -0.06  (-0.51─0.38) | 37 (18%) | -0.20  (-0.61─0.21) | 132 (22%) | -0.14  (-0.50─0.21) |
| **Accessory fans at the roasters** |  |  |  |  |  |  |  |  |
| No (intercept) | 107 (57%) | 1.04  (0.73─1.34) | 40 (54%) | 3.19  (2.97─3.40) | 77 (38%) | 2.11  (1.93─2.30) | 282 (48%) | 2.09  (1.93─2.25) |
| Yes | 82 (43%) | 0.18  (-0.43─0.78) | 34 (46%) | -0.14  (-0.55─0.27) | 126 (62%) | **-0.29**  **(-0.59─-0.003)** | 309 (52%) | **-0.30**  **(-0.56─-0.03)** |
| **Isolated roasting area** |  |  |  |  |  |  |  |  |
| No (intercept) | 178 (94%) | 1.13  (1.01─1.25) | 67 (91%) | 2.97  (2.84─3.10) | 186 (92%) | 1.85  (1.78─1.93) | 545 (92%) | 1.86  (1.79─1.93) |
| Yes | 11 (6%) | -0.14  (-1.39─1.11) | 7 (9%) | **1.35**  **(0.83─1.88)** | 17 (8%) | **1.18**  **(0.66─1.70)** | 46 (8%) | 0.83  (0.34**─**1.31) |
| **Flavoring Ventilation (LEV)** |  |  |  |  |  |  |  |  |
| Flavoring, no LEV | 15 (8%) | 1.53  (0.78-2.30) | 9 (12%) | 4.09  (3.65-4.52) | 21 (10%) | 3.16  (2.76─3.56) | 61 (10%) | 2.68  (2.31─3.05) |
| Flavoring, LEV | 28 (15%) | 0.89  (-0.23─2.00) | 10 (14%) | **-1.49**  **(-2.17─-0.82)** | 25 (12%) | **-0.63**  **(-1.15─-0.11)** | 95 (16%) | 0.02  (-0.47─0.51) |
| No flavoring during survey | 146 (77%) | -0.68  (-1.55─0.18) | 55 (74%) | -1.07  (-1.57─-0.57) | 157 (77%) | **-1.47**  **(-1.91─-1.04)** | 435 (74%) | **-1.01**  **(-1.42─-0.60)** |

**N_w_ indicates the number of workers sampled; N_t_ indicates the total number of samples; β is for every unit change in predictor; CI indicates 80% credible interval;**

***note small cell size (n<5);**

†**indicates variables with some information not recorded (missing values, accounted for in modeling).**

**Note: GM=e^(intercept + β)^**

**Supplementary Table 7.** Determinant distributions and univariate models for process level determinants of diacetyl and 2,3-pentanedione during quality control and flavoring tasks–**bold p<0.2**

| **Determinant** | **Quality Control**  **N_w_=9**  **N_t_=44** | | | | **Flavoring**  **N_w_=5**  **N_t_=15** | | | |
| --- | --- | --- | --- | --- | --- | --- | --- | --- |
|  | **Diacetyl**  **% censoring=25%** | | **2,3-pentanedione**  **% censoring=2%** | | **Diacetyl**  **% censoring=%** | | **2,3-pentanedione**  **% censoring=%** | |
|  | **N (%)**  **Med (range)** | **β (CI)** | **N (%)**  **Med (range)** | **β (CI)** | **N (%)**  **Med (range)** | **β (CI)** | **N (%)**  **Med (range)** | **β (CI)** |
| **Sample Level Determinants** | | | | | | | | |
| **Sampled task** |  |  |  |  |  |  |  |  |
| Roasting | 17 (39%) | 1.26  (0.81─1.70) | 17 (39%) | 1.49  (1.27─1.72) | ─ | ─ | ─ | ─ |
| Brewing, cupping, and tasting | 16 (36%) | 0.44  (-0.18─1.07) | 16 (36%) | 0.12  (-0.20─0.44) | ─ | ─ | ─ | ─ |
| Grinding | 11 (25%) | 0.51  (-0.19─1.21) | 11 (25%) | **0.49**  **(0.13─0.85)** | ─ | ─ | ─ | ─ |
| **Coffee Storage Determinants** | | | | | | | | |
| **Sum of all open storage sources** |  |  |  |  |  |  |  |  |
| Large (intercept)  (> 2) | 25 (57%) | 2.33  (2.19─2.46) | 25 (57%) | 1.97  (1.84─2.10) | 10 (67%) | 4.17  (2.98─5.37) | 10 (67%) | 4.22  (3.16─5.27) |
| Small (1-2) | 6 (14%) | -0.11  (-0.41─0.20) | 6 (14%) | **0.19**  **(-0.10─0.48)** | 2 (13%) | -0.54  (-3.44─2.32) | 2 (13%) | -0.43  (-3.03─2.17) |
| None | 13 (30%) | **-2.51**  **(-2.83─-2.20)** | 13 (30%) | **-1.13**  **(-1.35─-0.91)** | 3 (20%) | 100% <LOD | 3 (20%) | -1.54  (-3.74─0.67) |
| **General Sources Determinants** | | | | | | | | |
| **Total number of sources** |  |  |  |  |  |  |  |  |
| Large (intercept)  (> 7) | 25 (57%) | 2.33  (2.09─2.57) | 25 (57%) | 1.97  (1.80─2.13) | 12 (80%) | ‡ | 12 (80%) | 4.16  (3.22─5.09) |
| Small (≤ 7) | 19 (43%) | **-1.75**  **(-2.15─-1.36)** | 19 (43%) | **-0.72**  **(-0.97─-0.47)** | 3 (20%) | 100% < LOD‡ | 3 (20%) | -1.50  (-3.59─0.59) |
| **Process Determinants** | | | | | | | | |
| **Amount of roasted coffee produced** | | | | | | | | |
| **Average roasted coffee production, lbs per day** |  |  |  |  |  |  |  |  |
| Large (intercept)  **(≥** 10,000) | 25 (57%) | 2.33  (2.16─2.50) | 25 (57%) | 1.97  (1.82─2.12) | 10 (67%) | 4.17  (2.80─5.53) | 10 (67%) | 4.24  (3.21─5.26) |
| Medium  (≥ 1,000 and  < 10,000) | 10 (23%) | **-0.76**  **(-1.08─-0.45)** | 10 (23%) | **-0.33**  **(-0.61─-0.05)** | 0 (0%) | n/a | 0 (0%) | n/a |
| Small  (< 1,000) | 9 (20%) | 100% <LOD‡ | 9 (20%) | **-1.14**  **(-1.43─-0.85)** | 5 (33%) | **-3.99**  **(-6.76─-1.37)** | 5 (33%) | -1.13  (-2.91─0.65) |
| **Total number of roasters** |  |  |  |  |  |  |  |  |
| 1 (intercept) | 10 (23%) | 1.46  (0.91─2.01) | 10 (23%) | 1.63  (1.33─1.94) | 5 (33%) | 0.16  (-2.28─2.42) | 5 (33%) | 3.13  (1.69─4.59) |
| 2 | 34 (77%) | 0.12  (-0.50─0.75) | 34 (77%) | 0.03  (-0.31─0.38) | 10 (67%) | **4.00**  **(1.39─6.78)** | 10 (67%) | 1.11  (-0.68─2.89) |
| 3 | n/a | n/a | n/a | n/a | n/a | n/a | n/a | n/a |
| **Total roaster capacity in lbs** |  |  |  |  |  |  |  |  |
| Intercept | 670  (55─670) | 0.25  (-0.11─0.60) | 670  (55─670) | 1.11  (0.89**─**1.33) | 748  (26─1000) | -0.33  (-2.44─1.62) | 748  (26─1000) | 2.56  (1.19─3.94) |
| Slope |  | **0.003**  **(0.0025─0.0038)** |  | **0.0013**  **(0.001─0.002)** |  | **0.006**  **(0.003─0.008)** |  | **0.002**  **(0.0003─0.004)** |
| **Total number of package lines** |  |  |  |  |  |  |  |  |
| Small (intercept)  (< 4) | 19 (43%) | 0.58  (0.26─0.89) | 19 (43%) | 1.25  (1.06─1.44) | 5 (33%) | 0.16  (-2.28─2.42) | 5 (33%) | 3.13  (1.69─4.59) |
| Large  (≥ 4) | 25 (57%) | **1.75**  **(1.36─2.16)** | 25 (57%) | **0.72**  **(0.46─0.97)** | 10 (67%) | **4.00**  **(1.39─6.78)** | 10 (67%) | 1.11  (-0.68─2.89) |
| **Type of roasted coffee produced (roast depth)** | | | | | | | | |
| **Average roast length in minutes** |  |  |  |  |  |  |  |  |
| < 15 minutes | 40 (91%) | 1.70  (1.43─1.96) | 40 (91%) | 1.74  (1.60─1.88) | 2 (13%) | ¶ | 2 (13%) | ¶ |
| ≥ 15 minutes† | 4 (9%) | **-1.59**  **(-2.54─-0.66)** | 4 (9%) | **-0.95**  **(-1.44─-0.45)** | 13 (87%) | ¶ | 13 (87%) | ¶ |
| **Amount of grinding performed and grinding process determinants** | | | | | | | | |
| **Average percent ground coffee of total production** |  |  |  |  |  |  |  |  |
| High (≥ 25) | 31 (70%) | 2.31  (2.19─2.43) | 31 (70%) | 2.00  (1.89─2.12) | 12 (80%) | ‡ | 12 (80%) | 4.16  (3.22─5.09) |
| Low (< 25) | 13 (30%) | **-2.48**  **(-2.80─-2.18)** | 13 (30%) | **-1.17**  **(-1.38─-0.96)** | 3 (20%) | 100% < LOD | 3 (20%) | -1.50  (-3.59─0.59) |
| **Average grind length in mins** |  |  |  |  |  |  |  |  |
| Intercept | 15  (0.33─15) | 0.49  (0.14─0.83) | 15  (0.33─15) | 1.23  (1.03─1.43) | 47.5  (2─60) | 0.66  (-2.00─3.10) | 47.5  (2─60) | 3.57  (2.04─5.11) |
| Slope |  | **0.12**  **(0.09─0.15)** |  | **0.05**  **(0.03─0.07)** |  | **0.06**  **(0.007─0.11)** |  | 0.008  (-0.03─0.04) |
| **Total number of grinders** |  |  |  |  |  |  |  |  |
| Small (≤5) | 19 | 0.58  (0.26─0.89) | 19 | 1.25  (1.06─1.44) | 7 | 3.56  (1.89─5.16) | 7 | 4.70  (3.71─5.70) |
| Large (>5) | 25 | **1.75**  **(1.36─2.16)** | 25 | **0.72**  **(0.46─0.97)** | 8 | -1.48  (-3.96─1.08) | 8 | **-2.11**  **(-3.72─-0.53)** |
| **Total grinding capacity in lbs** |  |  |  |  |  |  |  |  |
| Intercept | 405  (4─405) | 0.36  (0.04─0.67) | 405  (4─405) | 1.15  (0.95─1.34) | 1001  (5─1285) | 0.79  (-1.75─3.13) | 1001  (5─1285) | 3.61  (2.13─5.12) |
| Slope |  | **0.005**  **(0.004─0.006)** |  | **0.002**  **(0.0015─0.003)** |  | **0.003**  **(0.0002─0.005)** |  | 0.0003  (-0.001─0.002) |
| **Grind flavored coffee** |  |  |  |  |  |  |  |  |
| Yes (intercept) | 0 (0%) | n/a | 0 (0%) | n/a | 11 (73%) | 1.86  (1.06─2.62) | 11 (73%) | 2.88  (2.23─3.52) |
| No | 44 (100%) | n/a | 44 (100%) | n/a | 4 (27%) | **5.22**  **(3.82─6.67)** | 4 (27%) | **3.78**  **(2.55─5.03)** |
| **Flavoring process determinants** | | | | | | | | |
| **Flavor (and package) ground coffee, accessory fans in flavoring room, and isolated flavoring room** |  |  |  |  |  |  |  |  |
| Flavor (and package) whole bean coffee, do not flavor ground coffee/no accessory fans/no isolated flavoring room | 0 (0%) | n/a | 0 (0%) | n/a | 5 (33%) | 0.16  (-2.28─2.42) | 5 (33%) | 3.13  (1.69─4.59) |
| Flavor (and package) ground and whole bean coffee/accessory fans/isolated flavoring room | 0 (0%) | n/a | 0 (0%) | n/a | 10 (67%) | **4.00**  **(1.39─6.78)** | 10 (67%) | 1.11  (-0.68─2.89) |
| Do not flavor coffee | 44 (100%) | n/a | 44 (100%) | n/a | 0 (0%) | n/a | 0 (0%) | n/a |
| **Flavoring during survey** |  |  |  |  |  |  |  |  |
| No flavoring (intercept) | 44 (100%) | n/a | 44 (100%) | n/a | 0 (0%) | n/a | 0 (0%) | n/a |
| Flavoring | 0 (0%) | n/a | 0 (0%) | n/a | 15 (100%) | n/a | 15 (100%) | n/a |
| **Engineering Controls Determinants** | | | | | | | | |
| **Percent automated sources** |  |  |  |  |  |  |  |  |
| None (intercept) | 13 (30%) | -0.18  (-0.48─0.10) | 13 (30%) | 0.83  (0.66─1.01) | 5 (33%) | 1.08  (-0.35─2.39) | 5 (33%) | 3.11  (2.11─4.12) |
| Low (≤ 6) | 25 (57%) | **2.51**  **(2.19─2.83)** | 25 (57%) | **1.13**  **(0.92─1.35)** | 6 (40%) | 1.24  (-0.41─2.97) | 6 (40%) | -0.46  (-1.84─0.89) |
| High* (> 6) | 6 (14%) | **2.40**  **(2.01─2.81)** | 6 (14%) | **1.33**  **(1.01─1.64)** | 4 (27%) | **6.00**  **(4.20─7.92)** | 4 (27%) | **3.56**  **(2.07─5.05)** |
| **Any isolated sources/processes** |  |  |  |  |  |  |  |  |
| No (intercept) | 0 (0%) | n/a | 0 (0%) | n/a | 5 (33%) | 0.16  (-2.28─2.42) | 5 (33%) | 3.13  (1.69─4.59) |
| Yes | 44 (100%) | n/a | 44 (100%) | n/a | 10 (67%) | **4.00**  **(1.39─6.78)** | 10 (67%) | 1.11  (-0.68─2.89) |
| **Any enclosed sources** |  |  |  |  |  |  |  |  |
| No (intercept) | 19 (43%) | 0.58  (0.26─0.89) | 19 (43%) | 1.25  (1.06─1.44) | 11 (73%) | 1.86  (1.06─2.62) | 11 (73%) | 2.88  (2.23─3.52) |
| Yes | 25 (57%) | **1.75**  **(1.36─2.16)** | 25 (57%) | **0.72**  **(0.46─0.97)** | 4 (27%) | **5.22**  **(3.82─6.67)** | 4 (27%) | **3.78**  **(2.55─5.03)** |
| **GEV (with mechanical supply)**† |  |  |  |  |  |  |  |  |
| Yes (intercept) | 38 (86%) | 1.45  (1.16─1.73) | 38 (86%) | 1.58  (1.43─1.73) | 13 (87%) | ¶ | 13 (87%) | ¶ |
| No | 6 (14%) | **0.77**  **(0.04─1.51)** | 6 (14%) | **0.58**  **(0.17─0.98)** | 2 (13%) | ¶ | 2 (13%) | ¶ |
| **Natural ventilation**† |  |  |  |  |  |  |  |  |
| Yes (intercept) | 15 (34%) | # | 15 (34%) | # | 3 (20%) | 100% < LOD‡ | 3 (20%) | 100% < LOD‡ |
| No* | 4 (9%) | # | 4 (9%) | # | 6 (40%) | ‡ | 6 (40%) | ‡ |
| **Accessory fans at the roasters** |  |  |  |  |  |  |  |  |
| No (intercept) | 19 (43%) | 0.58  (0.26─0.89) | 19 (43%) | 1.25  (1.06─1.44) | 6 (40%) | 5.95  (4.78─7.10) | 6 (40%) | 5.72  (4.70─6.73) |
| Yes | 25 (57%) | **1.75**  **(1.36─2.16)** | 25 (57%) | **0.72**  **(0.46─0.97)** | 9 (60%) | **-4.69**  **(-6.29─-3.15)** | 9 (60%) | **-3.07**  **(-4.39─-1.75)** |
| **Isolated roasting area** |  |  |  |  |  |  |  |  |
| No (intercept) | 44 (100%) | n/a | 44 (100%) | n/a | 9 (60%) | 3.56  (1.89─5.16) | 9 (60%) | 4.70  (3.71─5.70) |
| Yes | 0 (0%) | n/a | 0 (0%) | n/a | 6 (40%) | -1.48  (-3.96─1.08) | 6 (40%) | **-2.11**  **(-3.72─-0.53)** |
| **Flavoring Ventilation (LEV)** |  |  |  |  |  |  |  |  |
| Flavoring, no LEV | 0 (0%) | n/a | 0 (0%) | n/a | 8 (53%) | 2.50  (0.80─4.19) | 8 (53%) | 2.90  (1.83─3.95) |
| Flavoring, LEV | 0 (0%) | n/a | 0 (0%) | n/a | 7 (47%) | 1.00  (-1.59─3.49) | 7 (47%) | 2.05  (0.51─3.62) |
| No flavoring during survey | 44 (100%) | n/a | 44 (100%) | n/a | 0 (0%) | n/a | 0 (0%) | n/a |

**N_w_ indicates the number of workers sampled; N_t_ indicates the total number of samples; β is for every unit change in predictor; CI indicates 80% credible interval;**

***note small cell sizes (n<5);**

†**indicates variables with some information not recorded (missing values, accounted for in modeling);**

**‡indicates comparison group was 100% < LOD and no model run;**

**§indicates inability to run model due to small cell sizes and high censoring;**

¶**N<5 in groups and insufficient sample size to run model**

**#indicates samples collected from one facility and inability to model.**

**Note: GM=e^(intercept + β)^**

**Supplementary Figure S1. A panel of univariate analyses estimates of geometric means and 80% credible intervals for diacetyl concentrations by sample-level determinant category during roasting, grinding, packaging, and all tasks (overall).**
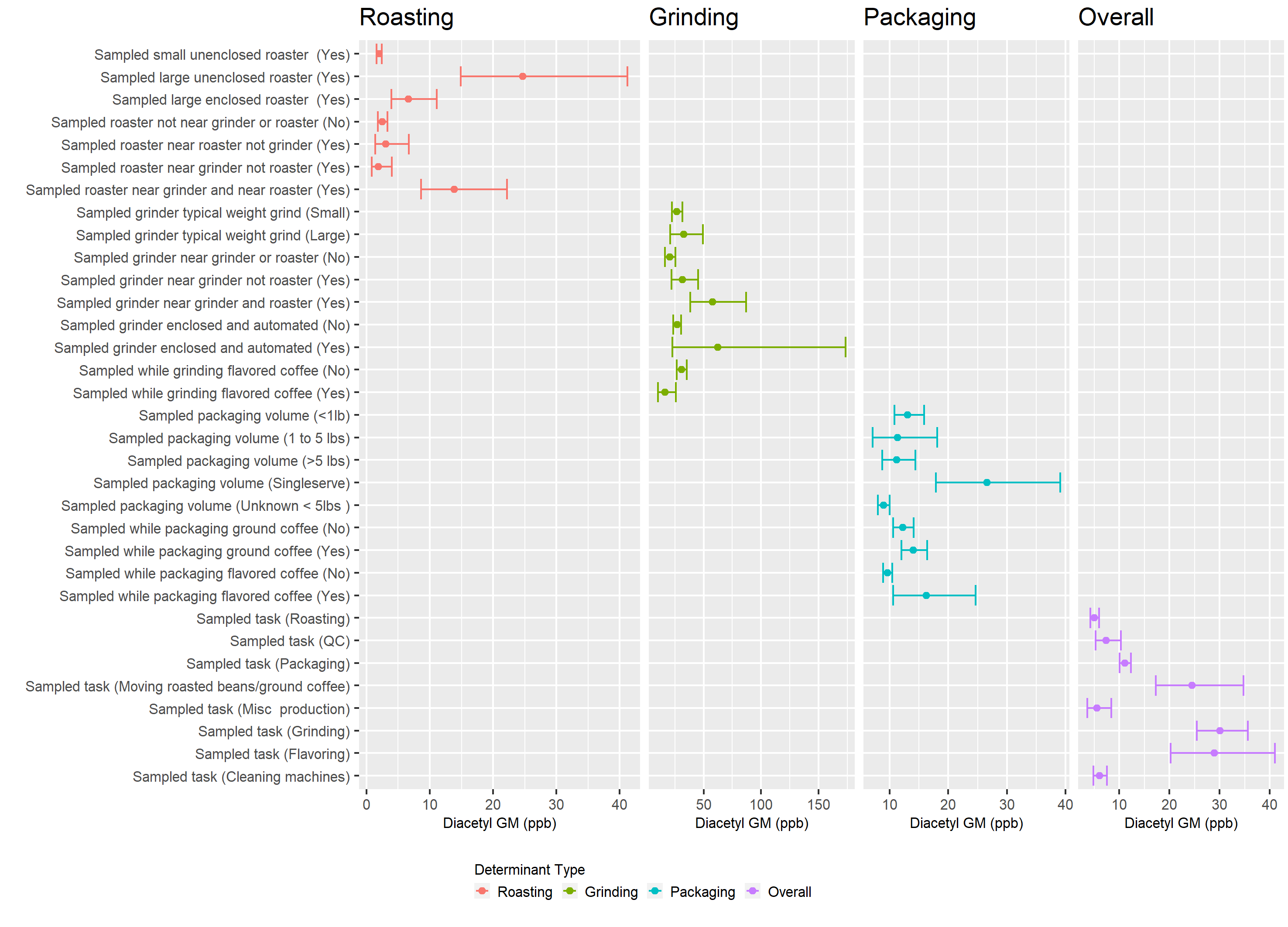


**Supplementary Figure S2. A panel of univariate analyses estimates of geometric means and 80% credible intervals for diacetyl concentrations by process-level determinant category during roasting, grinding, packaging, and all tasks (overall).**
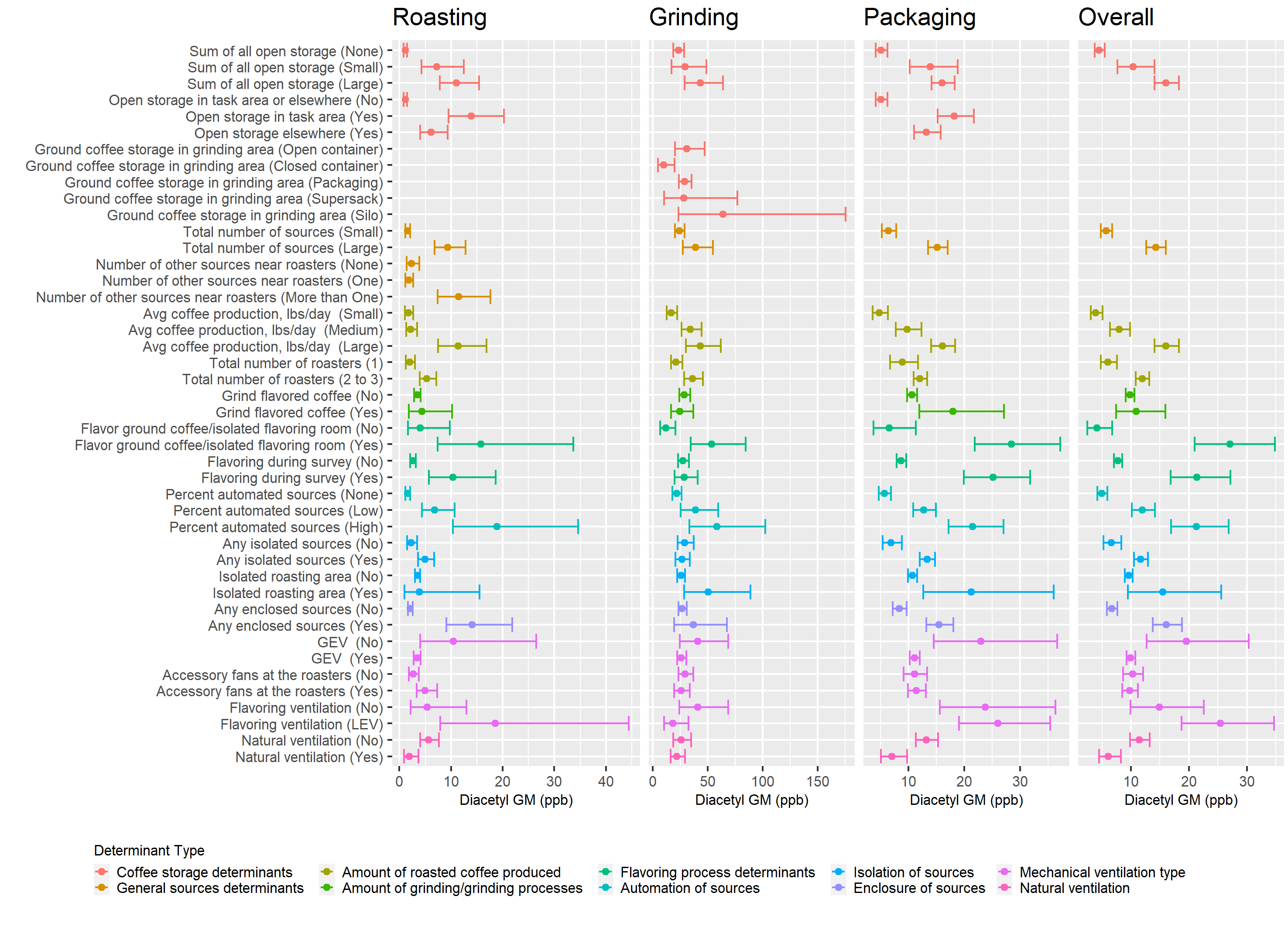


**Supplementary Figure S3. A panel of univariate analyses estimates of geometric means and 80% credible intervals for 2,3-pentanedione concentrations by sample-level determinant category during roasting, grinding, packaging, and all tasks (overall).**


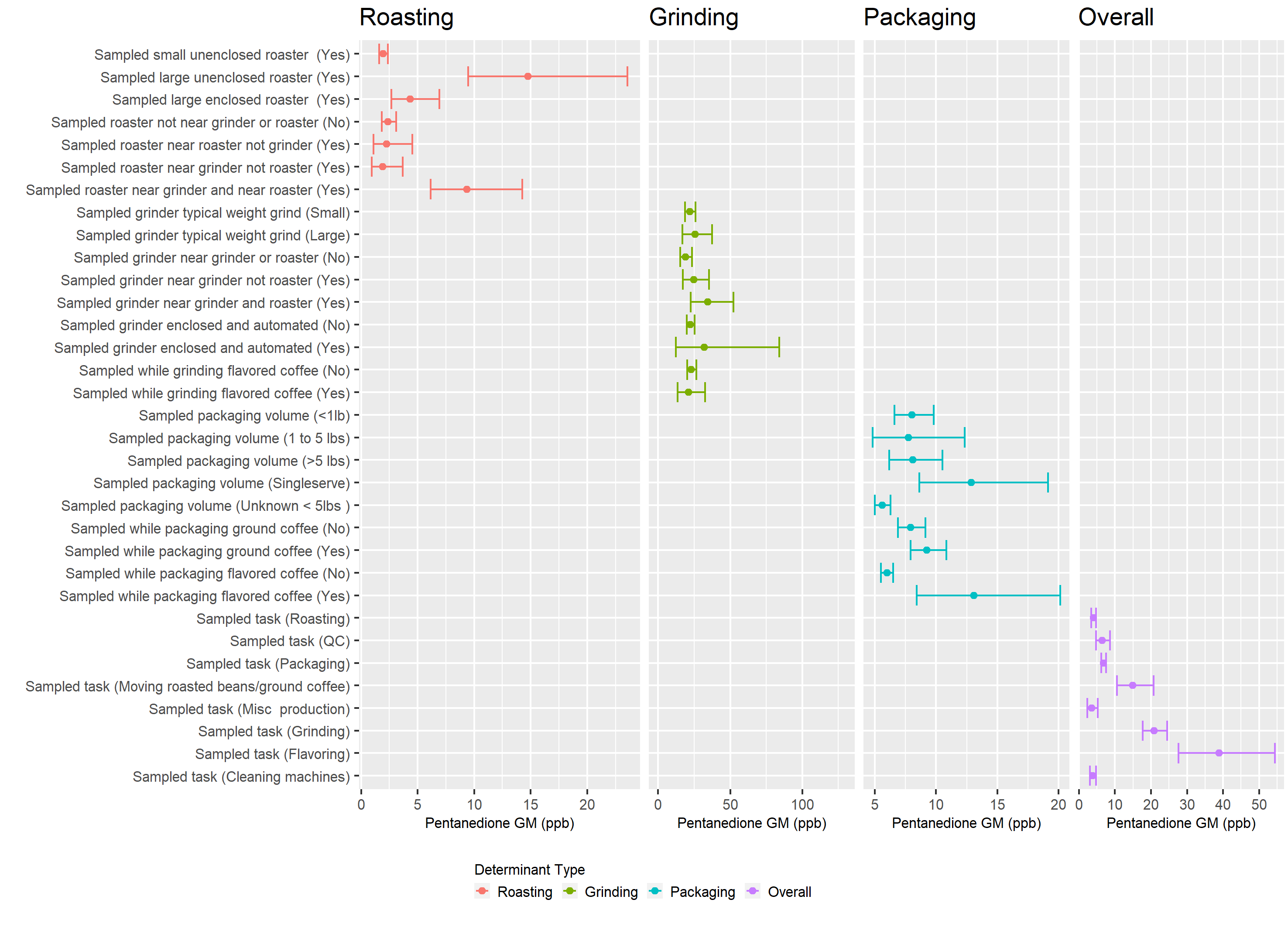


**Supplementary Figure S4. A panel of univariate analyses estimates of geometric means and 80% credible intervals for 2,3-pentanedione concentrations by process-level determinant category during roasting, grinding, packaging, and all tasks (overall).**


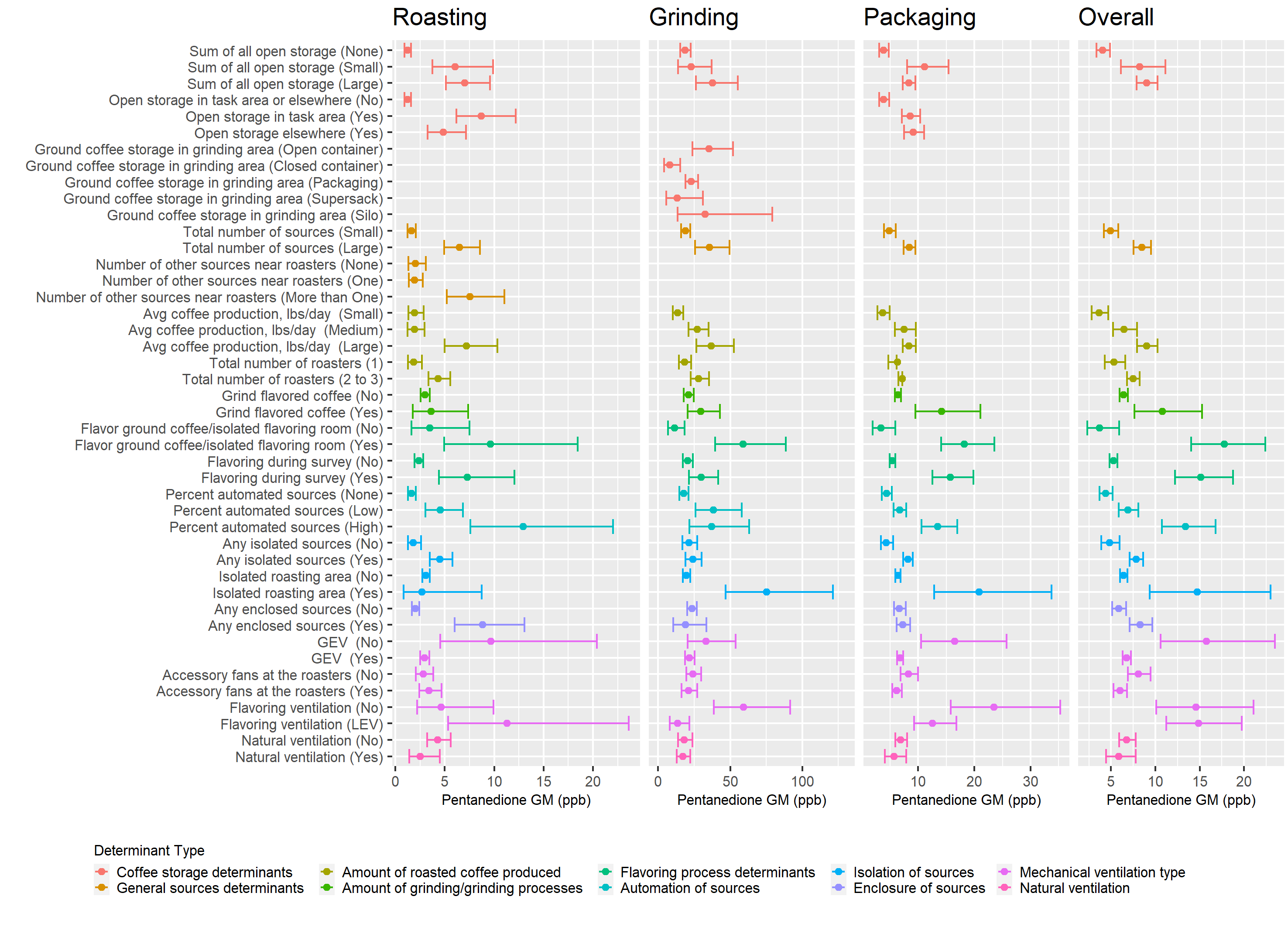


**Supplementary Figure S5: Collinearity matrix for determinants included in multiple linear regression models for diacetyl (A) and 2,3-pentanedione (B) exposures during all tasks (overall model). Grey indicates substantial collinearity present (at least one category >0.5), orange indicates nested determinants, and green indicates non-collinear determinants.**

1. **Diacetyl**


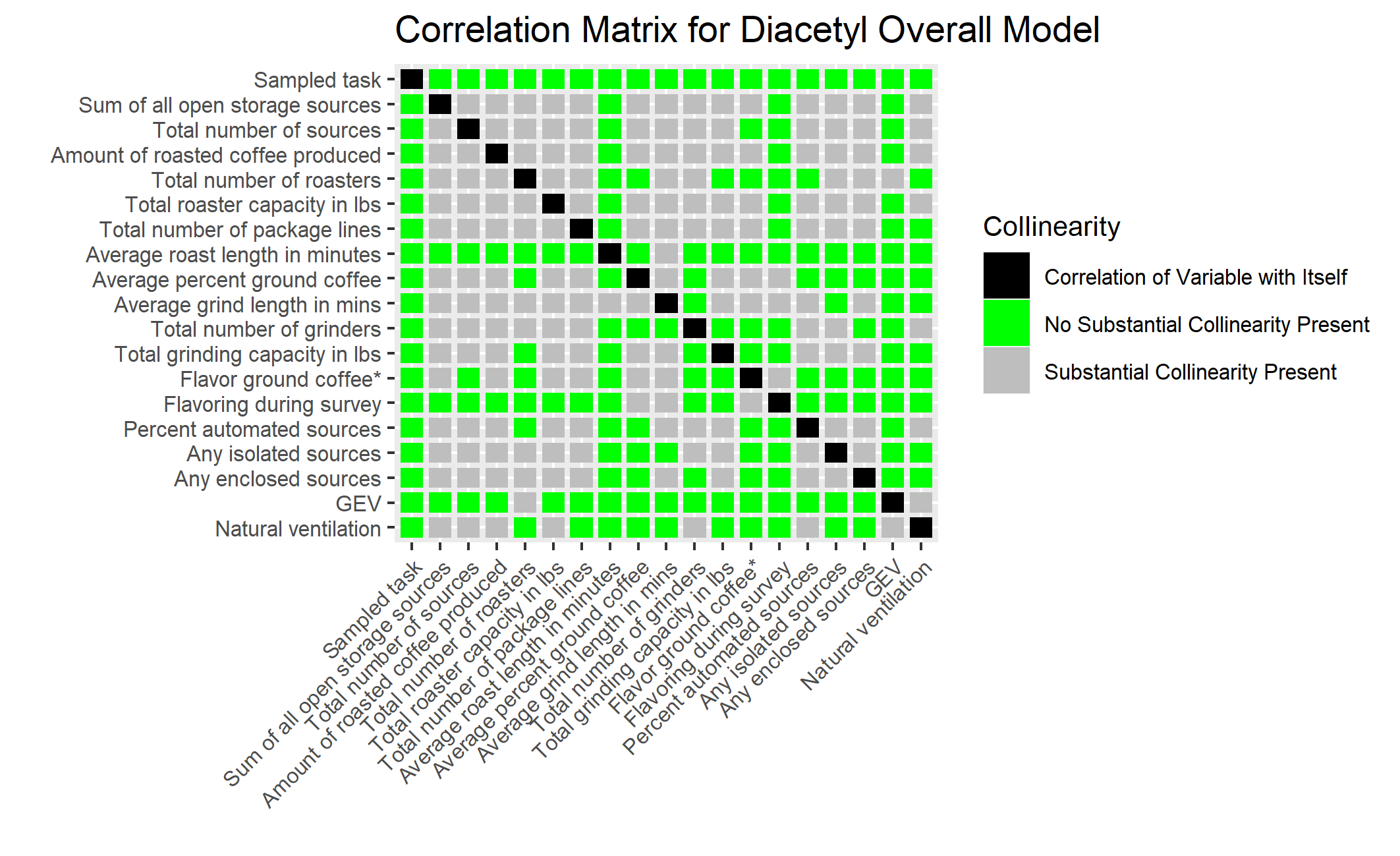


1. **2,3-pentanedione**

**
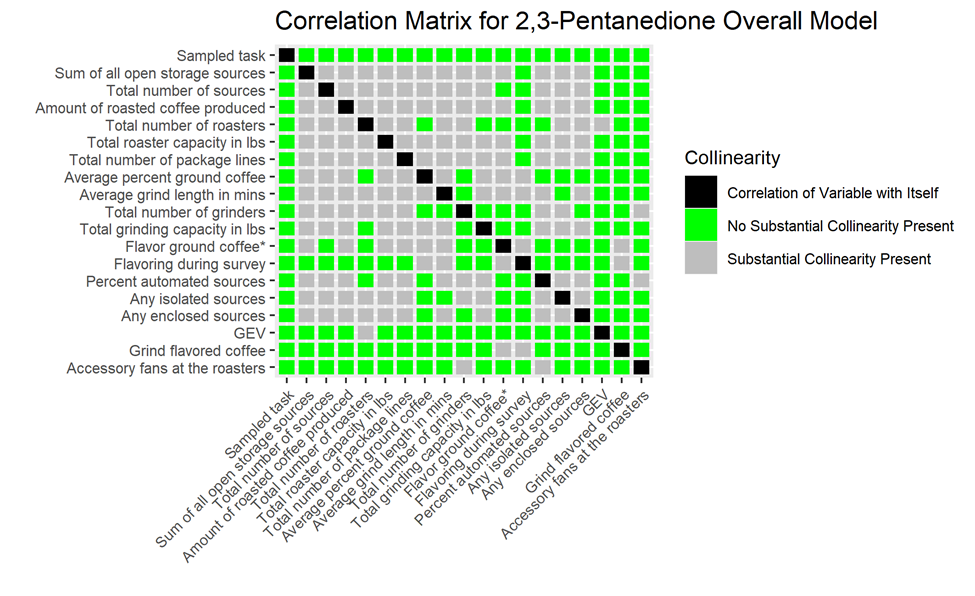
**

**Supplementary Figure S6: Collinearity matrix for determinants included in multiple linear regression models for diacetyl (A) and 2,3-pentanedione (B) exposures during roasting tasks. Grey indicates substantial collinearity present (at least one category >0.5), orange indicates nested determinants, and green indicates non-collinear determinants.**

1. **Diacetyl**

**
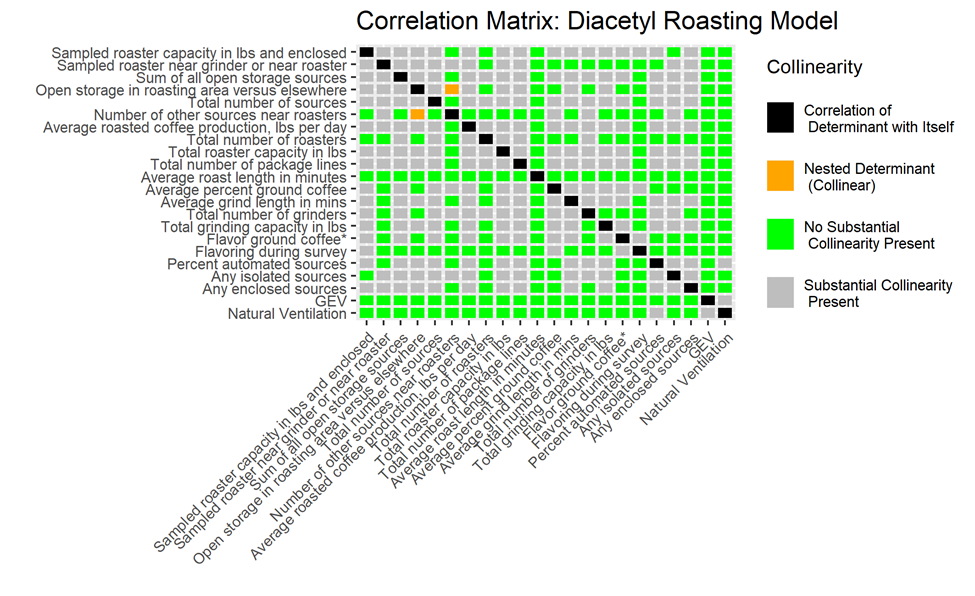
**

1. **2,3-pentanedione**

**
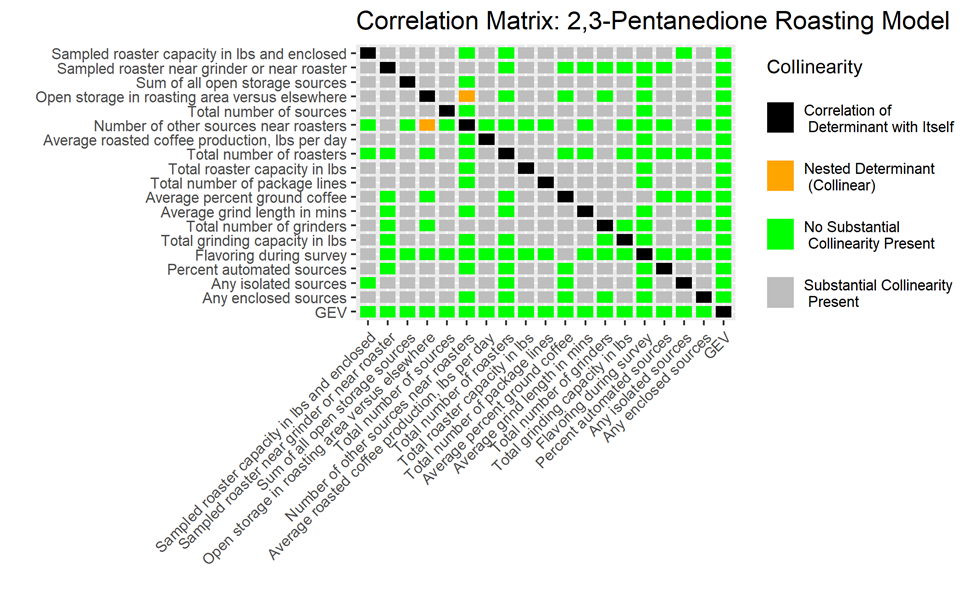
**

**Supplementary Figure S7: Collinearity matrix for determinants included in multiple linear regression models for diacetyl (A) and 2,3-pentanedione (B) exposures during grinding tasks. Grey indicates substantial collinearity present (at least one category >0.5), orange indicates nested determinants, and green indicates non-collinear determinants.**

1. **Diacetyl**


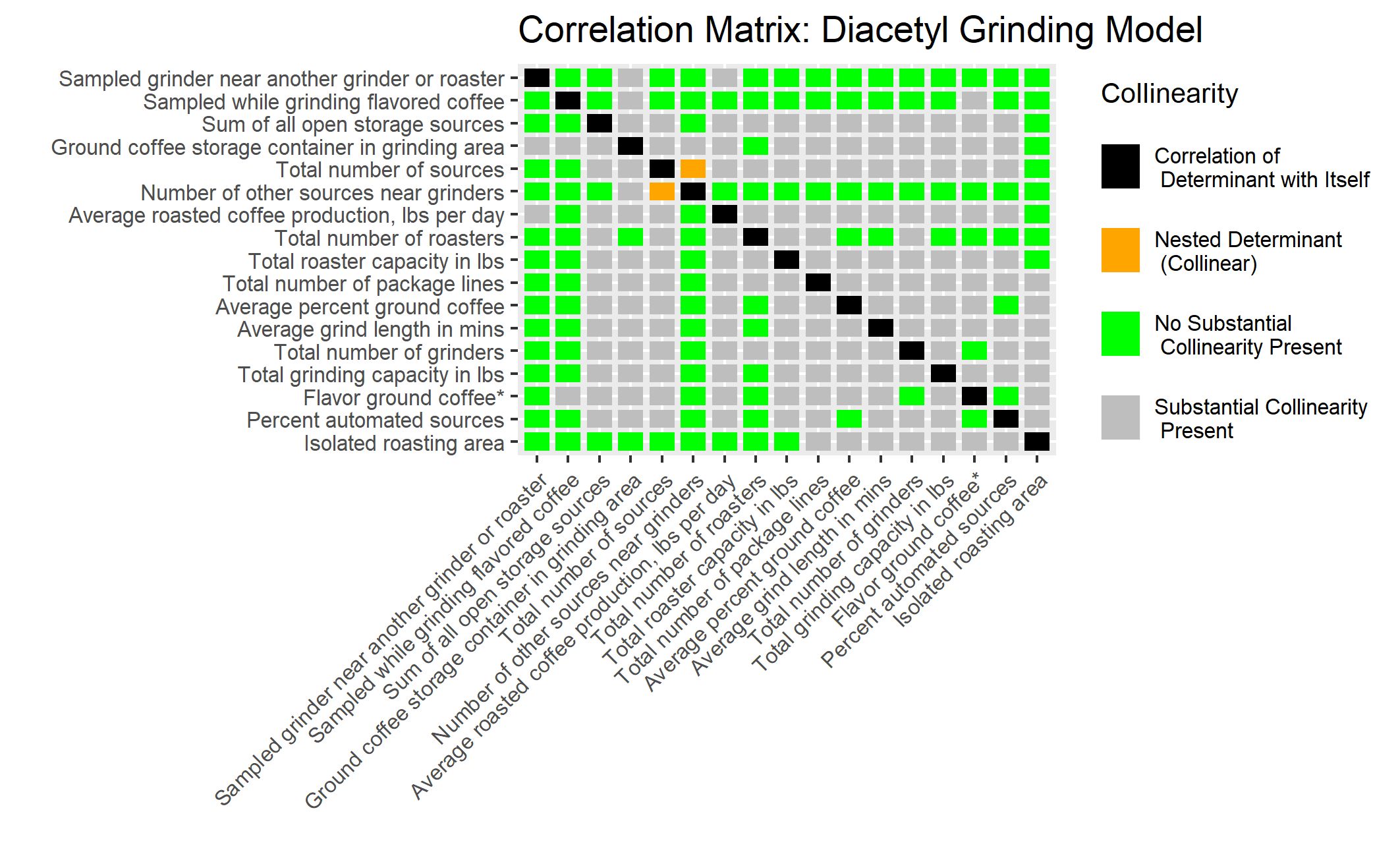


1. **2,3-pentanedione**


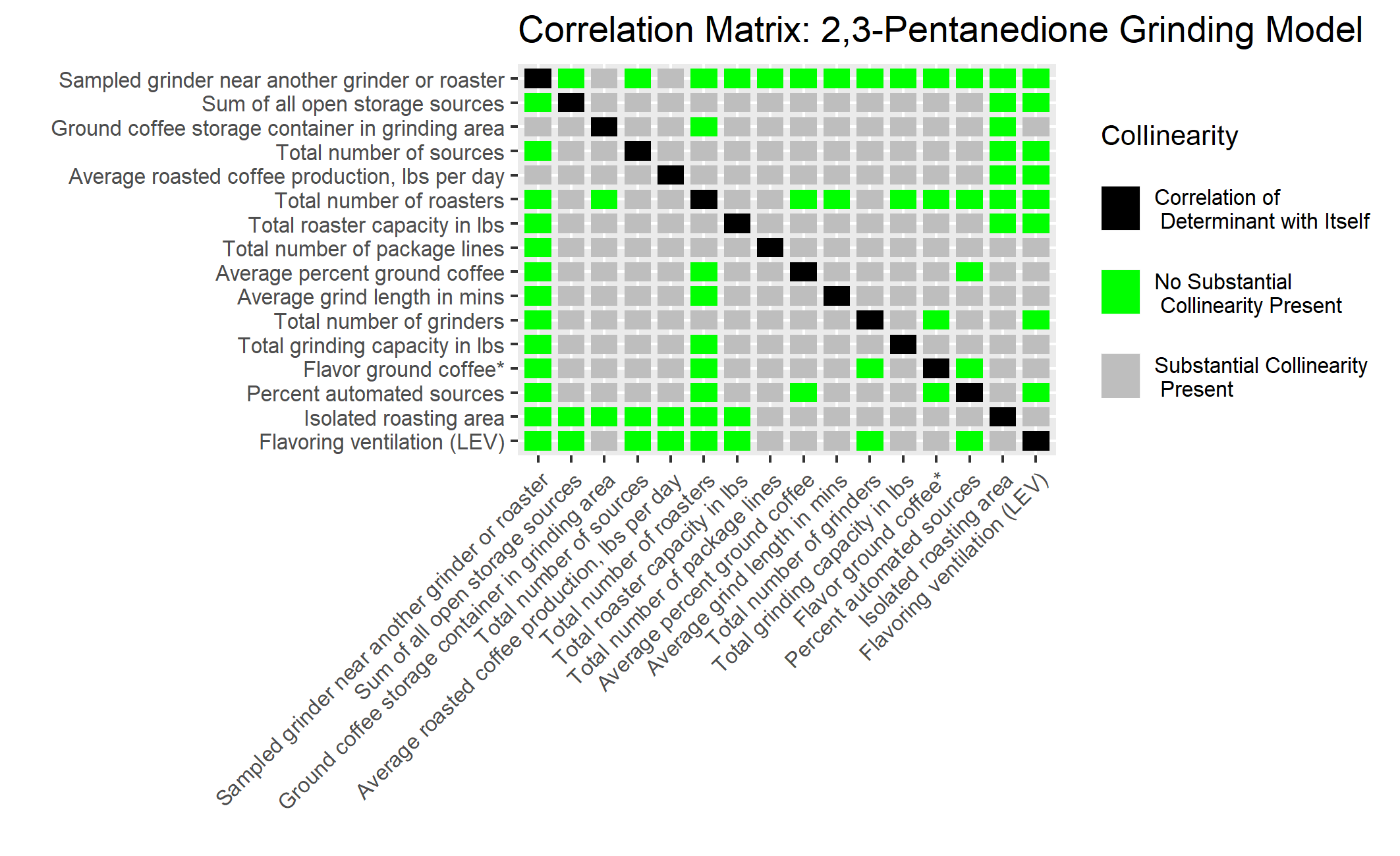


**Supplementary Figure S8: Collinearity matrix for determinants included in multiple linear regression models for diacetyl (A) and 2,3-pentanedione (B) exposures during packaging tasks. Grey indicates substantial collinearity present (at least one category >0.5), orange indicates nested determinants, and green indicates non-collinear determinants.**

1. **Diacetyl**


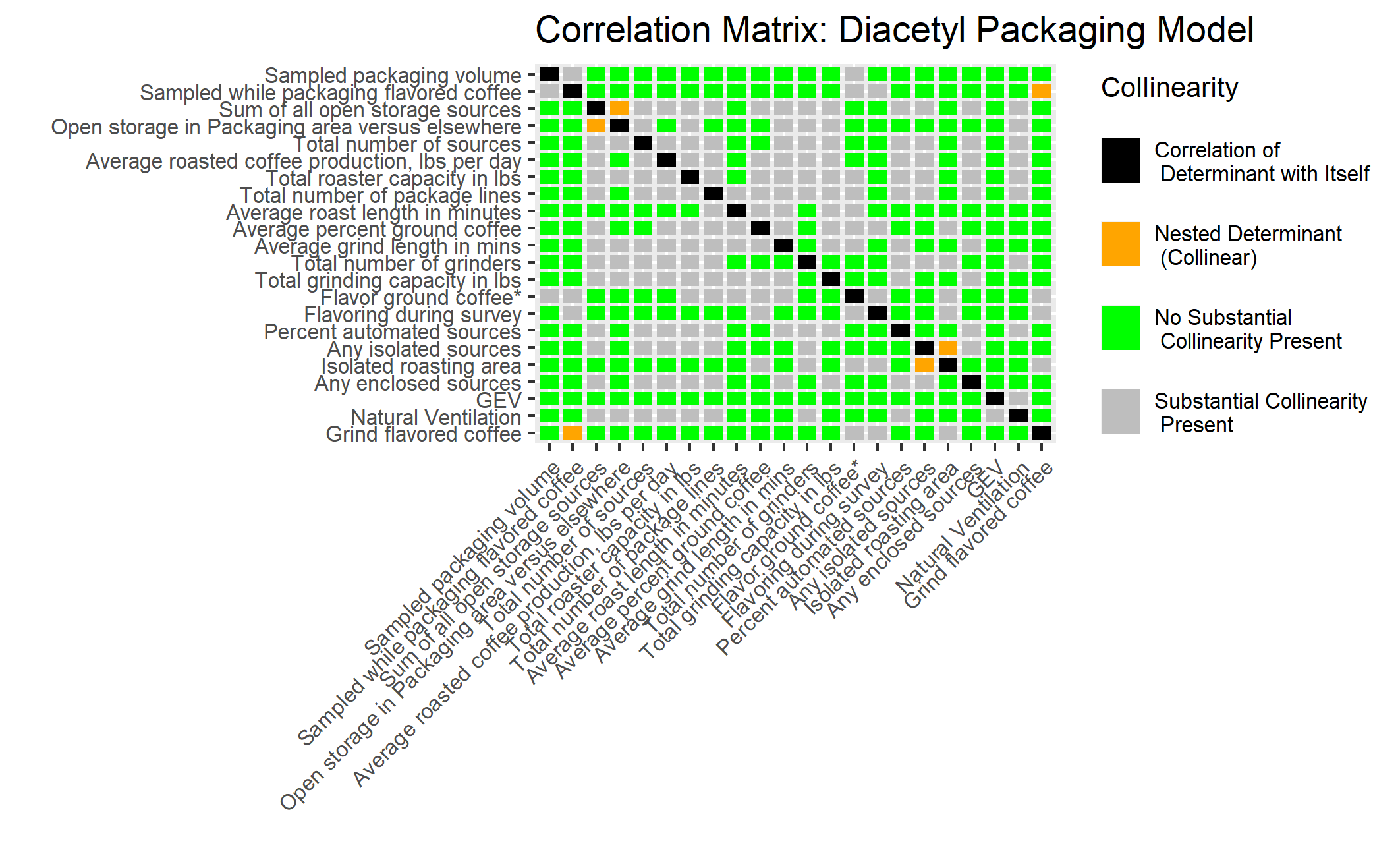


1. **2,3-Pentanedione**


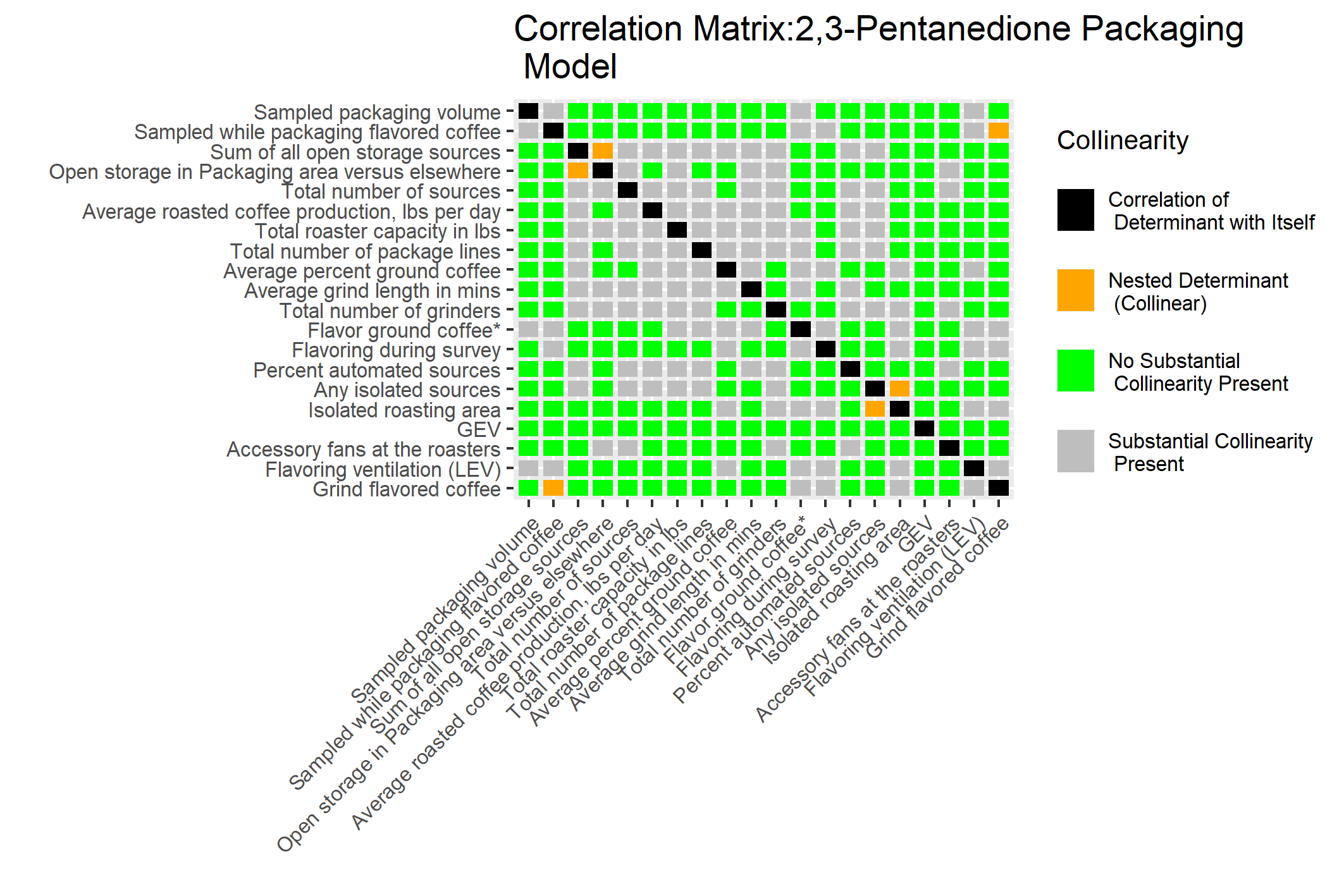

Supplement: Supplementary file 1 [file Data_Sheet_1.DOCX]
